# Mechanistic investigations of the Fe(ii) mediated synthesis of squaraines†

**DOI:** 10.1039/d4sc01286k

**Published:** 2024-05-07

**Authors:** Yu Liu, Nathan T. Coles, Nathalia Cajiao, Laurence J. Taylor, E. Stephen Davies, Alistair Barbour, Patrick J. Morgan, Kevin Butler, Ben Pointer-Gleadhill, Stephen P. Argent, Jonathan McMaster, Michael L. Neidig, David Robinson, Deborah L. Kays

**Affiliations:** a School of Chemistry, University of Nottingham, University Park Nottingham NG7 2RD UK Deborah.Kays@nottingham.ac.uk Nathan.Coles@nottingham.ac.uk; b Inorganic Chemistry Laboratory, Department of Chemistry, University of Oxford South Parks Road Oxford OX1 3QR UK; c Department of Chemistry, University of Rochester Rochester New York 14627 USA; d Department of Chemistry and Forensics, School of Science and Technology, Nottingham Trent University Nottingham NG11 8NS UK

## Abstract

The scission and homologation of CO is a fundamental process in the Fischer–Tropsch reaction. However, given the heterogeneous nature of the catalyst and forcing reaction conditions, it is difficult to determine the intermediates of this reaction. Here we report detailed mechanistic insight into the scission/homologation of CO by two-coordinate iron terphenyl complexes. Mechanistic investigations, conducted using *in situ* monitoring and reaction sampling techniques (IR, NMR, EPR and Mössbauer spectroscopy) and structural characterisation of isolable species, identify a number of proposed intermediates. Crystallographic and IR spectroscopic data reveal a series of migratory insertion reactions from 1^Mes^ to 4^Mes^. Further studies past the formation of 4^Mes^ suggest that ketene complexes are formed en route to squaraine 2^Mes^ and iron carboxylate 3^Mes^, with a number of ketene containing structures being isolated, in addition to the formation of unbound, protonated ketene (8). The synthetic and mechanistic studies are supported by DFT calculations.

## Abstract 2

*In situ* monitoring and reaction sampling (IR, NMR, EPR, Mössbauer spectroscopy) and structural characterisation of isolable species allows detailed mechanistic insight into the scission/homologation of CO by two-coordinate iron terphenyl complexes.

## Graphical abstract

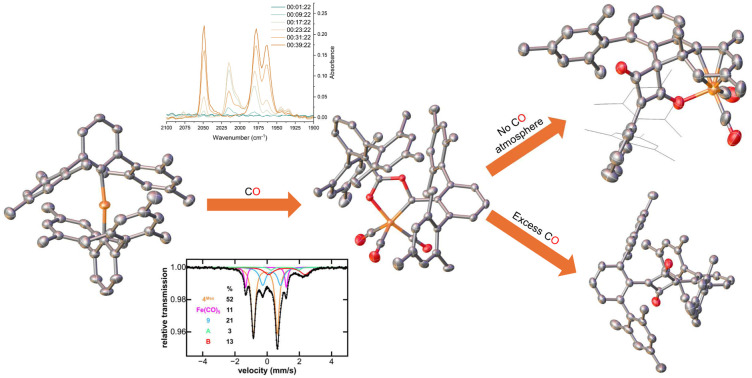

## 1. Introduction

The reduction and homologation of CO to obtain high-value organic compounds is a long-standing area of research interest. In industry, the Fischer–Tropsch process utilises heterogeneous catalysts and forcing conditions to convert CO, in the presence of H_2_, to hydrocarbon fuels.^1,2^ However, such reactions are unselective, affording products with a range of chain lengths.^3–5^ As such, the reaction of CO with homogeneous species to selectively generate specific organic products remains a tantalising prospect. Over the years, many examples of such reactions have been published from across the periodic table, utilising s-block,^6–11^ p-block,^12–16^ d-block,^17–25^ and f-block elements.^26–31^ Not only do these reactions provide interesting organic products, but the mechanisms of these reactions are also of significant interest. Often, a complex series of steps facilitate this reduction and homologation process.^32,33^ Understanding these reactions allows us greater insight into not only the chemistry of the elements involved, but also the utilisation of CO on an industrial scale. The Fischer–Tropsch process, being a heterogeneous reaction, is challenging to study mechanistically. The investigation of CO reduction by soluble transition metal complexes can, therefore, provide valuable information about the reactions that may occur in such a system.

**Scheme 1:**
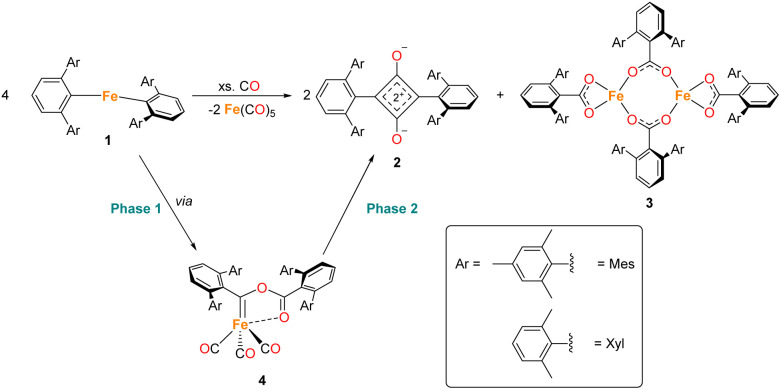
General reaction scheme for the reaction between 1 in the presence of CO, where Ar = Mes or Xyl. The two phases of the reaction are shown, phase 1 covers the reaction of 1 to 4, phase 2 covers the reaction from 4 to 2, 3 and Fe(CO)_5_.

The first example of insertion of CO into a low-coordinate Fe(ii) *m*-terphenyl complex was reported by Ni and Power.^34^ Upon exposure of the complex Fe(C_6_H_3_-2,6-Dipp_2_)_2_ (Dipp = 2,6-iPr_2_C_6_H_3_) to an atmosphere of CO, the complex Fe(CO)_2_[C(O)C_6_H_3_-2,6-Dipp_2_]_2_ (Dipp = 2,6-iPr_2_C_6_H_3_) was obtained. Subsequently, we reported the selective reduction and homologation of CO by the less bulky Fe(ii) *m*-terphenyl complexes (2,6-Ar_2_C_6_H_3_)_2_Fe (Ar = Mes (2,4,6-Me_3_C_6_H_2_), 1^Mes^; Ar = Xyl (2,6-Me_2_C_6_H_3_), 1^Xyl^).^35^ The reaction proceeded at room temperature and 1 atm CO in toluene over 10 days. The final isolated products were a highly unusual squaraine (2^Mes^, 2^Xyl^, Scheme 1) featuring broken conjugation between the C_4_ and aryl rings, as well as Fe(CO)_5_ and an iron carboxylate (3^Mes^, 3^Xyl^, Scheme 1). This reaction was particularly noteworthy for the complete scission of the strong C≡O bond (1072 kJ mol^−1^), which is unusual for a process occurring under such mild conditions. Reactions with the 1-naphthyl (Naph) substituted analogue (2,6-Naph_2_C_6_H_3_)_2_Fe (1^Naph^) afforded an isolable iron carbene complex (CO)_3_Fe[C(2,6-Ar_2_C_6_H_3_)OC(O)(2,6-Ar_2_C_6_H_3_)] (Ar = 1-Naph 4^Naph^), but this species did not show further reactivity to 2 and 3. Regardless, analogous iron carbene complexes (4^Mes^, 4^Xyl^) were proposed as intermediates in the reaction between 1^Mes^ and 1^Xyl^ with CO.

Herein, we report detailed mechanistic investigations of the homologation and scission of CO by a two-coordinate Fe(ii) complex using *in situ* monitoring techniques (IR, NMR and EPR spectroscopy), frozen-solution Mössbauer (MB) spectroscopy and structural characterisation of intermediates *via* single crystal X-ray crystallography (scXRD). These experimental results are supported by DFT studies. From this, we propose a plausible mechanistic pathway, which highlights the unusual reactivity that can be facilitated using sterically demanding ligands which enforce low coordination environments at a metal centre.

## 2. Results and discussion

### 2.1 Overview

During the course of reaction monitoring (see discussion below) it became clear that the transformation of 1^Mes^–3^Mes^, and Fe(CO)_5_ proceeds in two distinct stages (Scheme 1). In phase 1, 1^Mes^ reacts with CO, *via* several reactive intermediates, to afford an iron–carbene complex 4^Mes^ (Scheme 1), which becomes the dominant species in solution. Phase 2 of the reaction involves 4^Mes^ reacting with further equivalents of CO resulting in the formation of a number of species, and eventually the final products 2^Mes^, 3^Mes^ and Fe(CO)_5_. For simplicity, we will discuss these two phases of the reaction separately. Sections 2.2 and 2.3 will focus on phase 1, covering all intermediates and observations up to the formation of 4^Mes^. Sections 2.4–2.7 will cover phase 2, looking at potential intermediates and pathways between 4^Mes^ and the final products.

### 2.2 Spectroscopic analysis of phase 1

**Scheme 2:**
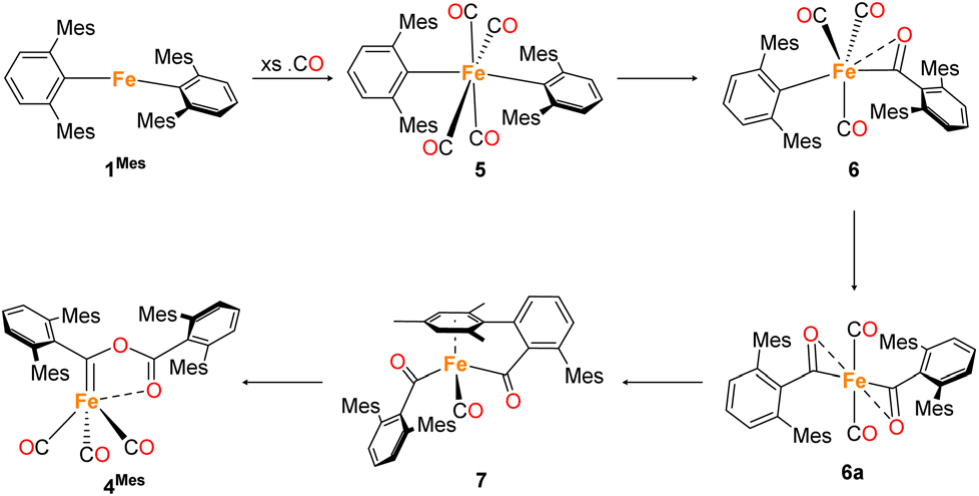
Proposed reaction pathway for formation of 4^Mes^ from 1^Mes^. Note that 6a has only been observed spectroscopically in solution, all other species have been isolated and characterised by single crystal X-ray diffraction (See Section 2.3).

**Fig. 1:**
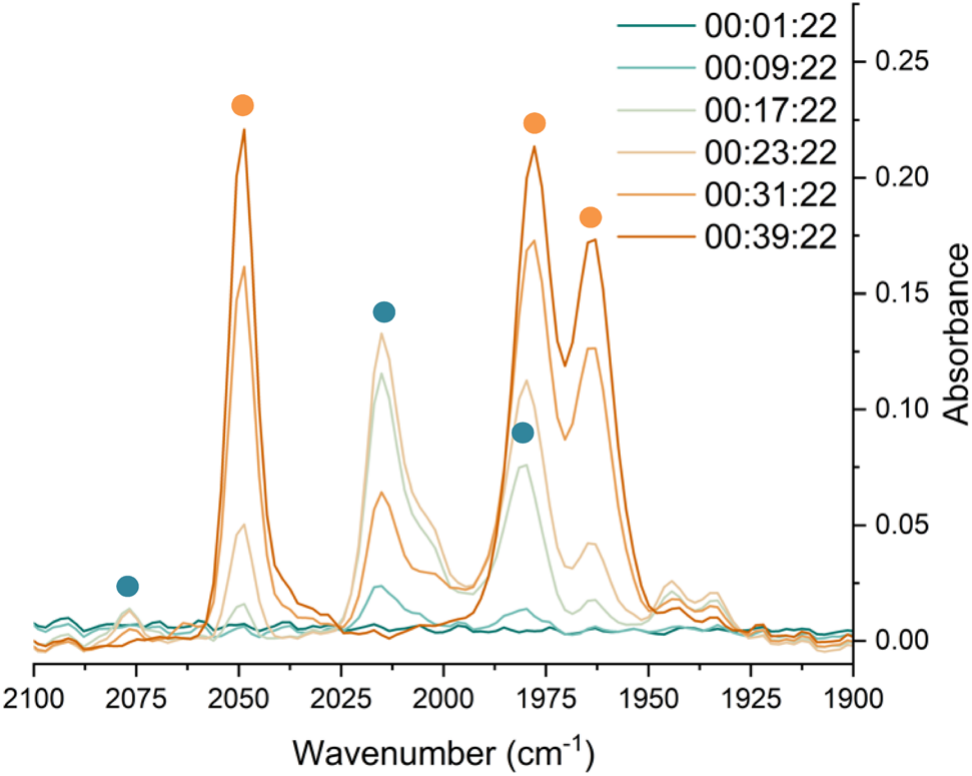
IR spectra for the first 40 minutes of the reaction between 1^Mes^ and CO in toluene. CO added to the reaction after six minutes. The colour gradient goes from dark turquoise (earliest) to dark orange (latest). Orange circles highlight the signals for 4^Mes^, turquoise circles highlight the signals attributed to 6.

*In situ* IR spectroscopy was conducted to monitor the transformation of the iron complex Fe(C_6_H_3_-2,6-Mes_2_)_2_ (1^Mes^) in the presence of CO over the course of the reaction. We postulate that the first step is the coordination of four CO molecules to 1^Mes^ affording Fe(CO)_4_(C_6_H_3_-2,6-Mes_2_)_2_ (5, Scheme 2). IR spectra of the reaction between 1^Mes^ and *ca.* 1 atm of CO in toluene were recorded at two-minute intervals. Within the first hour of the reaction, several carbonyl-containing species form which are subsequently consumed (Fig. 1). Initial intense signals at *ν*(CO) = 2014 cm^−1^, 2004 cm^−1^ and 1979 cm^−1^ were accompanied by less intense signals at *ν*(CO) = 2076 cm^−1^, 1942 cm^−1^ and 1935 cm^−1^. Following this, less intense peaks are observed at *ν*(CO) = 2063 cm^−1^ and 2035 cm^−1^ after approximately 30 minutes. The signals at *ν*(CO) = 2076 cm^−1^, 2014 cm^−1^ and 1979 cm^−1^ can be attributed to 6 which has been isolated and characterised independently in the solid state (See Section 2.3). After *ca.* 40 minutes of *in situ* monitoring the strongest IR bands occur at *ν*(CO) = 2049 cm^−1^, 1978 cm^−1^ and 1965 cm^−1^, which are assigned to 4^Mes^. These are similar to the previously reported 4^Naph^ [*ν*(CO) = 2043 cm^−1^, 1972 cm^−1^ and 1954 cm^−1^], a structural analogue of 4^Mes^.^35^ It has not been possible to assign the remaining peaks to specific species, but they are postulated to be metal carbonyl complexes.

**Fig. 2:**
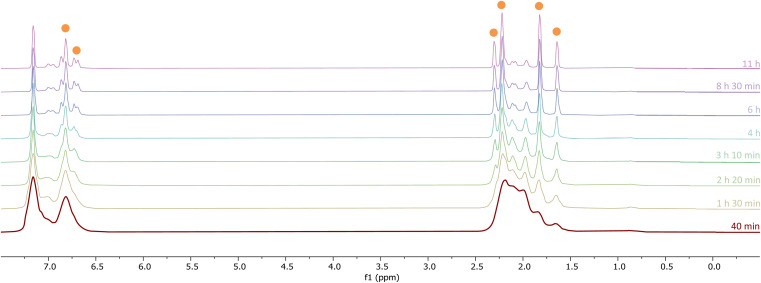
^1^H NMR spectra (−1 to 8 ppm) of the reaction between CO and 1^Mes^ (C_6_D_6_, 1 atm CO, room temperature) recorded between 40 minutes and 11 hours of reaction. Orange circles highlight the signals for 4^Mes^.

The initial stages of the reaction were also monitored *via*^1^H NMR spectroscopy. 1^Mes^ is a paramagnetic complex, and displays resonances over the range +80 to −180 ppm. However, upon introduction of CO, several new ^1^H NMR signals appear in the range 0–8 ppm. This is attributed to the formation of diamagnetic 18e^−^ iron complexes. Forty minutes after addition of CO, several species form, resulting in a set of overlapping signals that cannot be resolved from one another (Fig. 2). As the reaction proceeds four signals become dominant at *δ*_H_ = 2.31 ppm, 2.23 ppm, 1.83 ppm and 1.65 ppm, consistent with a species featuring terphenyl moieties in two separate environments. The IR and ^1^H NMR spectroscopic data indicate one major species is formed, 4^Mes^ (see Section 2.3). Full consumption of 1^Mes^ takes *ca.* 36 h, with concomitant formation of a paramagnetic species (See Fig. S45–S47, ESI†).

The use of ^13^CO allows the observation of intermediates by ^13^C{^1^H} NMR spectroscopy, showing the simultaneous formation of several compounds which contain CO in the form of acyl/carbene functional groups (250–270 ppm), metal-bound CO (220–200 ppm) and esters (176 ppm). Ni and Power have previously reported Fe(CO)_2_[C(O)C_6_H_3_-2,6-Dipp_2_]_2_ (Dipp = 2,6-iPr_2_C_6_H_3_) which showed carbonyl and acyl signals at *δ*_C_ = 214.8 ppm and 258.5 ppm, respectively. In our work, integration of the ^13^C{^1^H} NMR of spectra after ∼30 minutes of being placed under an atmosphere of ^13^CO shows that the signals at *δ*_C_ = 259.5 ppm and 206.0 ppm occur in a 1 : 3 ratio, while the signals at *δ*_C_ = 257.6 ppm and 214.7 ppm occur in a 1 : 1 ratio (Fig. S48, ESI†). This suggests the formation of structures of the type 6 and 6a, respectively, en route to 4^Mes^.

We note that, when monitoring the reaction using a ReactIR spectrometer, conversion to the carbene (4^Mes^) is complete within 40 minutes, but this takes *ca*. 36 hours when monitoring by *in situ*^1^H NMR spectroscopy. This is presumably due to a smaller headspace of CO, smaller interfacial surface area and, less efficient mixing. Paramagnetic species are also formed after 4^Mes^, which hinders further *in situ* monitoring by NMR spectroscopy.

### 2.3 Synthesis and structural characterisation of iron complexes observed during phase 1

Through careful control of the reaction conditions *via* solvent choice and reaction monitoring, it is possible to isolate the iron–carbene (CO)_3_Fe[C(2,6-Mes_2_C_6_H_3_)OC(O)(2,6-Mes_2_C_6_H_3_)] 4^Mes^, (Scheme 2), in preparative quantities. The reaction between 1^Mes^ and an atmosphere of CO in hexane affords an orange solution from which 6 precipitates (Scheme 2, see discussion below), after *ca*. 10 minutes. The reaction mixture is then stirred until complete redissolution of 6 occurs, followed by an additional 10 minutes of stirring. Filtration of the dark orange-red solution followed by removal of the volatiles allows the isolation of 4^Mes^ in 95% yield. 4^Mes^ has been characterised by ^1^H and ^13^C{^1^H} NMR spectroscopy (Fig. S1 and S2, ESI†), IR spectroscopy, mass spectrometry and elemental analysis. The NMR spectra confirm that the signals observed at *δ*_H_ = 2.31 ppm, 2.23 ppm, 1.83 ppm and 1.65 ppm in the ^1^H NMR spectroscopic monitoring of the reaction between CO and 1^Mes^ (Fig. 2) are due to 4^Mes^. The ATR-FTIR spectrum of 4^Mes^ (Fig. S18 and S19, ESI†) displays three strong stretches which are observed at *ν*(CO) = 2046 cm^−1^, 1974 cm^−1^ and 1959 cm^−1^ corresponding to the metal-bound CO groups, with a less intense stretch at *ν*(CO) = 1612 cm^−1^ for the carboxyl group bound to the carbene. Again, this is consistent with 4^Mes^ being the major species present after 40 minutes during *in situ* IR reaction monitoring (Fig. 1, Section 2.2).

**Fig. 3:**
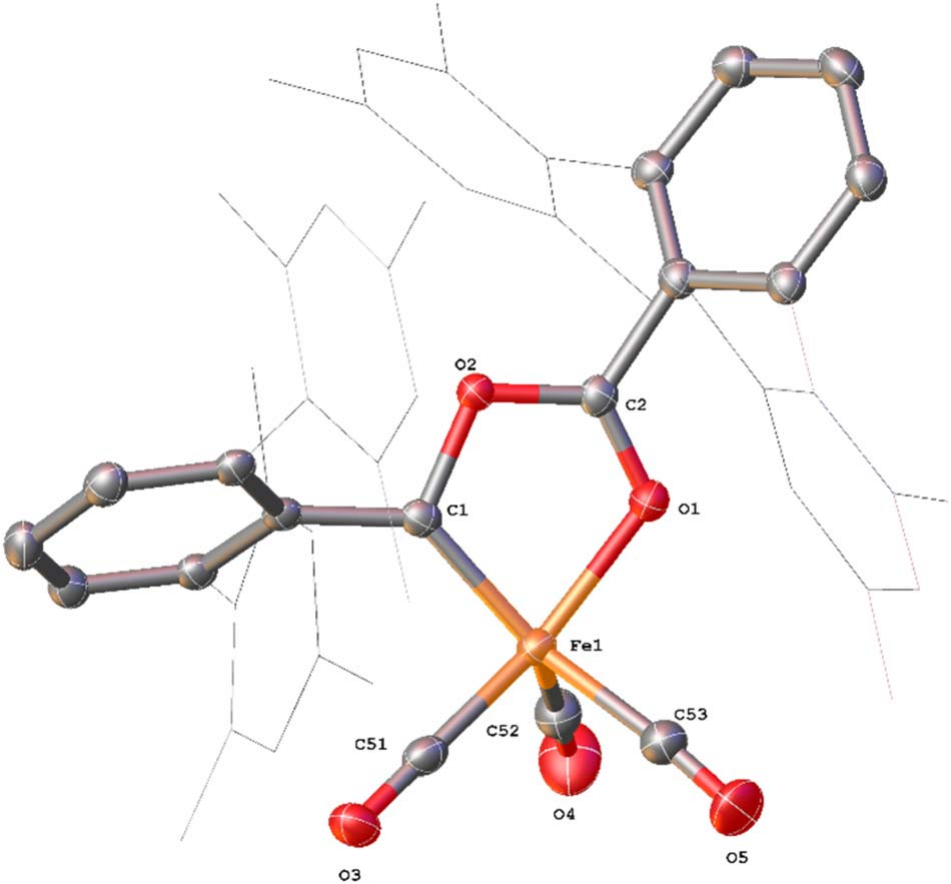
View of the metal complex from the crystal structure of 4^Mes^ with anisotropic displacement ellipsoids set at 50% probability. Mesityl groups shown as wireframe, hydrogen atoms, two co-crystallised diethyl ether and a second equivalent of 4^Mes^ have been omitted for clarity. Selected bond distances (Å) and angles (°) for 4^Mes^ shown: Fe1–C1 1.8395(14), Fe1–O1 1.9470(11), Fe1–C51 1.7549(16), Fe1–C52 1.828(2), Fe1–C53 1.8382(19), Fe1–C1–O1 80.81(5), O1–C2–O2 118.77(13).

**Fig. 4:**
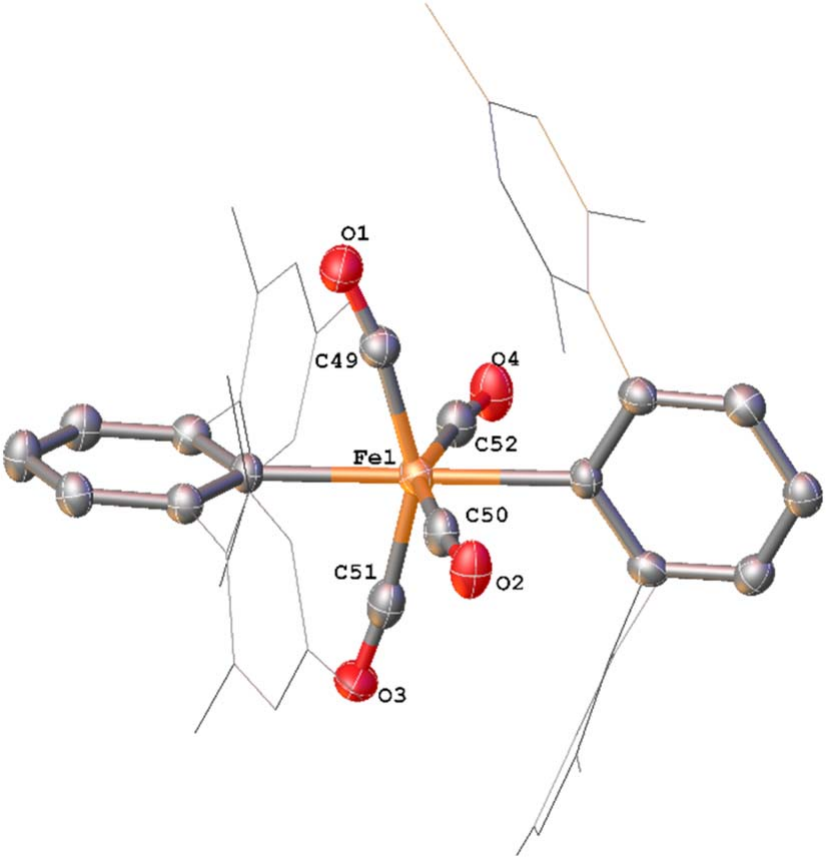
View of the metal complex from the crystal structure of 5 with anisotropic displacement ellipsoids set at 50% probability. Mesityl groups shown as wireframe and hydrogen atoms have been omitted for clarity. Selected bond distances (Å) and angles (°) for 5 shown: Fe1–C1 2.156(3), Fe1–C25 2.171(3), Fe1–C49 1.808(3), Fe1–C50 1.821(3), Fe1–C51 1.812(4), Fe1–C52 1.817(4), C1–Fe1–C25 174.58(11), C49–Fe1–C51 152.64(15), C50–Fe1–C52 153.63(15).

4^Mes^ has also been characterised by single crystal X-ray diffraction. Two solvatomorphs have been isolated; 4^Mes^ (Fig. 3) and 4^Mes^·Et_2_O (see ESI, Fig. S36 and Table S2†), both of which have been grown from concentrated Et_2_O solutions at low temperature. The Fe1–C1 bond length 4^Mes^ is near identical to the analogous distance for the previously published 4^Naph^ [1.8395(14) Å and 1.840(3) Å, respectively] suggesting the presence of an Fe=C bond.^35^

**Fig. 5:**
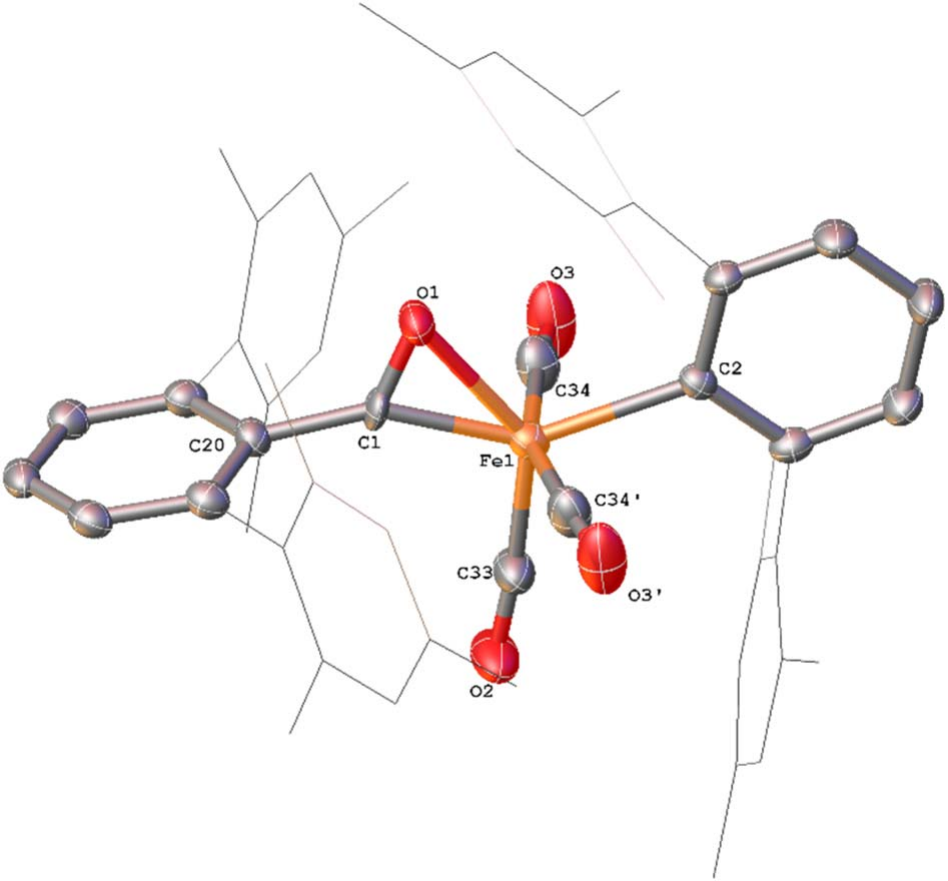
View of the metal complex from the crystal structure of 6 with anisotropic displacement ellipsoids set at 50% probability. Mesityl groups shown as wireframe and hydrogen atoms have been omitted for clarity. The following symmetry operations were used to generate the marked atoms: +*x*,3/2 −*y*,+*z* Selected bond distances (Å) and angles (°) for 6 shown: C1–O1 1.221(4), C33–O2 1.148(5), C34–O3 1.130(4), Fe1–C1 1.872(3), Fe1–C2 2.104(4), Fe1–O1 2.110(3) C20–C1–O1 124.6(3) Fe1–C1–O1 83.2(2).

**Fig. 6:**
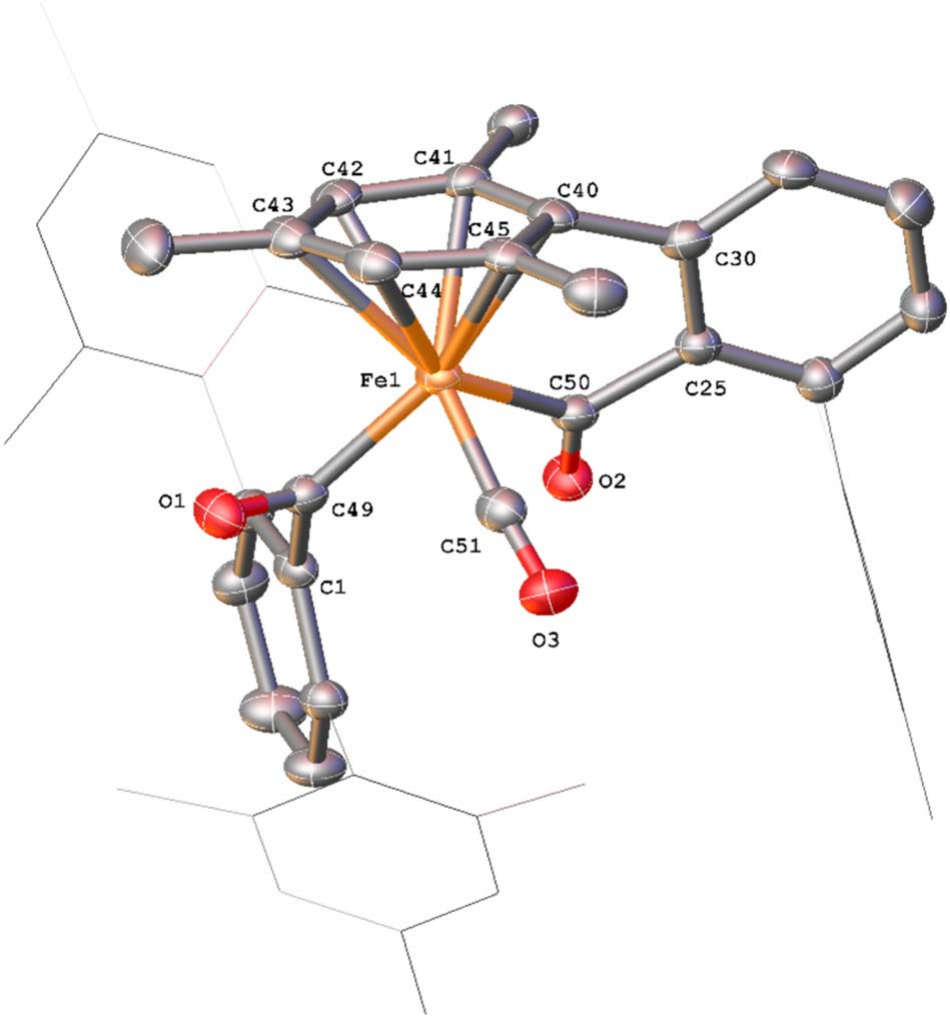
View of the metal complex from the crystal structure of 7 with anisotropic displacement ellipsoids set at 50% probability. Mesityl groups shown as wireframe and hydrogen atoms have been omitted for clarity. Selected bond distances (Å) and angles (°) for 7 shown: C49–O1 1.221(3), C50–O2 1.204(3), Fe1–C49 1.997(2), Fe1–C50_0_ 1.986(2), C1–C49–O1 116.24(19), C25–C50–O2 120.7(2), Fe1–C49–C1 129.13(15), Fe1–C50–C25 111.44(15), Fe1–C49–O1 114.59(17), Fe1–C50_0_–O2 127.87(18).

Whilst intermediate species between 1^Mes^ and 4^Mes^ are highly reactive, it has been possible to crystallise proposed intermediates of this transformation (Scheme 2). The reaction between 1^Mes^ and 1 atm of CO in benzene resulted in a colour change from yellow to orange. Five minutes after the change in colour, the reaction mixture was flash frozen in liquid N_2_. The solvent was then sublimed off under vacuum, resulting in the formation of a red solid. This solid was then extracted three times with iso-hexane, keeping the extractions separate and these solutions were cooled to 8 °C for 48 hours. From these solutions, crystals of Fe(CO)_4_(C_6_H_3_-2,6-Mes_2_)_2_ (5), Fe(CO)_3_[C(O)C_6_H_3_-2,6-Mes_2_](C_6_H_3_-2,6-Mes_2_) (6) and Fe(CO)[C(O)C_6_H_3_-2,6-Mes_2_]_2_ (7) suitable for X-ray crystallography were obtained (Fig. 4–6). These represent the coordination of four CO molecules to 1^Mes^, followed by sequential migratory insertion reactions. Crystals of these complexes were isolated from mixtures containing several metal-containing species, therefore, it has not been possible to isolate 5, 6 or 7 in sufficient purity or quantities for full analysis.

The solid-state structure of 5 (Fig. 4), features a rare *trans*-arrangement of the terphenyl ligands,^36–40^ which is closer to linearity than 1^Mes^ [C1–Fe1–C25 angle of 174.57(10)°].^41^ Complex 5 shows a near-octahedral geometry at the iron(ii) centre with bent CO ligands due to the steric hindrance of the terphenyl groups.

Complex 6 (Fig. 5) features one *m*-terphenyl ligand, three carbonyls and an acyl ligand formed through the migratory insertion of one CO ligand into the Fe–C bond of the second *m*-terphenyl ligand. The acyl ligand coordinates in an η^2^-bonding mode, affording an 18e^−^ complex. These bond lengths and angles are similar to those observed for the diacyl complex Fe(CO)_2_[C(O)C_6_H_3_-2,6-Dipp_2_]_2_ which features a longer Fe–C bond than in 6 (1.8964(18) Å *vs.* 1.872(3) Å) but a decreased Fe–O distance (2.0229(14) Å *vs.* 2.110(3) Å) for the acyl group.^34^ Reaction of 1^Mes^ (200 mg) in iso-hexane (20 mL) under an atmosphere of CO yielded an orange precipitate (15% yield) corresponding to intermediate 6. ATR-FTIR analysis of 6 revealed CO stretches at 2077 cm^−1^, 2013 cm^−1^ and 1975 cm^−1^ (Fig. S22 and 23, ESI†) which correspond with those observed during the *in situ* measurements in toluene (Fig. 1; *ν*(CO) = 2076 cm^−1^, 2014 cm^−1^ and 1979 cm^−1^). The carbonyl stretch corresponding to the acyl ligand in this complex is observed at 1615 cm^−1^. Mass spectrometric analysis of 6 using MALDI-TOF allowed the observation of the [M−2(CO)]^+^ ion (see ESI†). When dissolving 6 to obtain an NMR spectrum, 4^Mes^ was detected within 10 minutes, even though there was no 4^Mes^ present in the ATR-IR spectrum of the solid, demonstrating their intrinsic high reactivity. *In situ*^13^C{^1^H} NMR studies of the reaction between 1^Mes^ and ^13^CO signals give signals at *δ*_C_ = 259.5 ppm and 206.0 ppm (integral ratio of 1 : 3, Fig. S48, ESI†), which have been tentatively assigned to 6.

The solid state structure of 7 (Fig. 6) features one CO and two η^1^-acyl ligands, one of which also binds to the Fe *via* an η^6^-mesityl group, affording an 18e^−^ metal centre. This differs substantially to the Dipp-substituted species Fe(CO)_2_[C(O)C_6_H_3_-2,6-Dipp_2_]_2_, in which the complex bears two η^2^-acyl moieties and two CO ligands.^34^ This difference is attributed to the lower steric demands of the mesityl substituents in 7 relative to the bulky 2,6-diisopropylphenyl moieties. As a result, the acyl ligands of 7 show significantly different bond lengths and angles relative to 6, consistent with η^1^-coordination, most notably increased Fe–C_(acyl)_ bond lengths [1.997(2) Å (Fe1–C49) and 1.986(2) Å (Fe1–C50)]. We propose that 7 is likely only observed in the solid state as, unlike 6, there are no signals in the ^13^CO-labelled ^13^C{^1^H} NMR spectrum that can be assigned to 7. In a solution saturated with CO we propose 7 exists as 6a (Scheme 2) with two bound CO ligands, akin to Fe(CO)_2_(C(O)C_6_H_3_-2,6-Dipp_2_)_2_. This is based on similarities within the ^13^CO-labelled ^13^C{^1^H} NMR spectra where signals were observed at *δ*_C_ = 257.6 ppm and 214.7 ppm (1 : 1 ratio) for 6a, *cf.* acyl: *δ*_C_ = 258.5 ppm, carbonyl: 214.8 ppm for Fe(CO)_2_(C(O)C_6_H_3_-2,6-Dipp_2_)_2._^34^

To further probe these structural insights, DFT calculations were employed. The calculations show a low barrier to the formation of 6 from 5 (21.9 kJ mol^−1^), while a further migratory insertion reaction occurs to form 6a (Fig. S86, ESI†) before rapid formation of 7, with a barrier height relative to 5 of only 24.1 kJ mol^−1^. The reaction then proceeds further to, followed by rapid reaction to further intermediates. DFT calculations suggest the barrier to formation of 6a from 6 is *ca*. 70 kJ mol^−1^ without solvent, confirming the relative stability of 6.

### 2.4 Spectroscopic analysis of phase 2

**Scheme 3:**
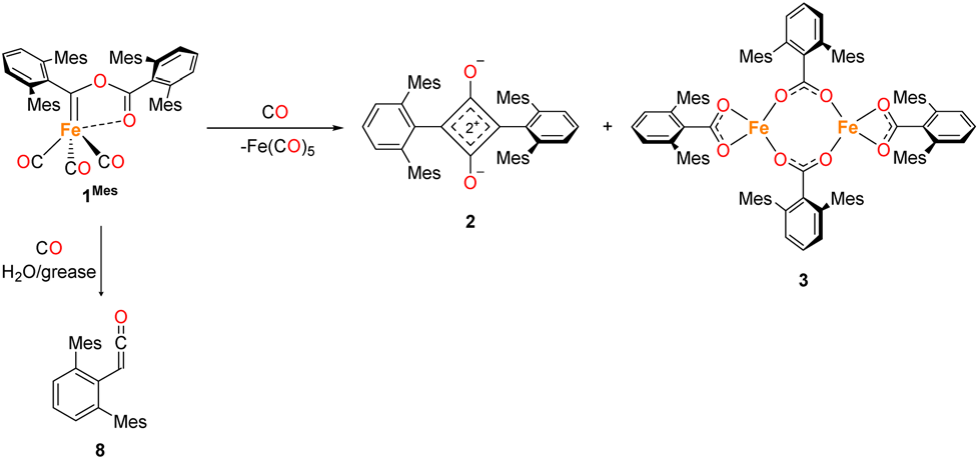
General reaction scheme post the formation of 4^Mes^ and the observed degradation product upon reaction with moisture/grease.

Reaction monitoring of phase 2 towards the final products (Scheme 3) squaraine (2^Mes^), Fe(CO)_5_ and Fe_2_[O_2_C(2,6-MesC_6_H_3_)]_4_ (3), which takes up to an additional 8 days post formation of 4^Mes^, has proven more challenging. Through ReactIR, it was impossible to avoid ingress of small amounts of water and/or oxygen into the flask, even when a continuous positive atmosphere of CO was employed, leading to side reactions of the highly reactive intermediates. Additionally, 3 is an insoluble, paramagnetic solid that precipitates during the course of the reaction, which interferes with *in situ* NMR spectroscopic monitoring.

To obtain an endpoint IR spectrum upon completion of the reaction, 1^Mes^ was reacted in toluene under *ca.* 1 atm of CO for 7 days. The reaction was filtered to remove insoluble 3 and an IR spectrum of the resulting solution was obtained (Fig. S45, ESI†). The observed peaks were assigned as 2 (1674 cm^−1^), Fe(CO)_5_ (1996 cm^−1^, 2024 cm^−1^) and, at 2097 cm^−1^, a ketene (O=C=C(H)C_6_H_3_-2,6-Mes_2_, 8) which we propose results from reaction with moisture and/or silicone grease. Ketene 8 has been characterised in reaction mixtures by ^1^H NMR, ^13^C{^1^H} NMR and IR spectroscopy, and mass spectrometry (see ESI, Fig. S6, S7, S31, S32 and S50†), although it has not been possible to isolate this as the sole product.

**Fig. 7:**
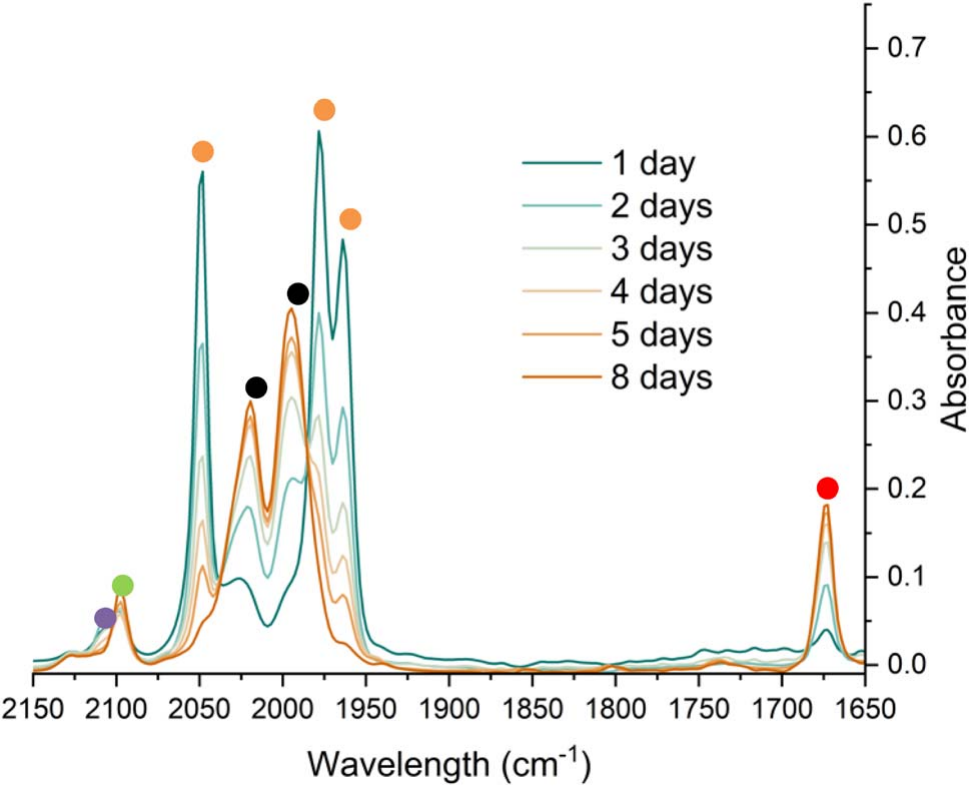
IR spectroscopic monitoring using a Harrick cell of the reaction of 4^Mes^ under an atmosphere of CO in toluene over 8 days. Orange circles highlight the signals for 4^Mes^, red circles highlight the signals for 2^Mes^. Black circles highlight the signals for Fe(CO)_5_. The green circle highlights the signal for 8. The purple circle highlights a signal attributed to a proposed Fe–ketenyl complex, 9 (see Fig. 8 for proposed structure). See ESI† for a zoom in of the region between 2150–2075 cm^−1^.

To monitor phase 2, by IR spectroscopy, a toluene solution of 4^Mes^ was reacted with CO in a J. Young reaction flask. Periodically, an aliquot of the reaction was filtered to remove insoluble 3 and transferred to a sealable IR cell. Over the course of 8 days, signals corresponding to 4^Mes^ (*ν*(CO) = 2049 cm^−1^, 1978 cm^−1^ and 1965 cm^−1^) are consumed (Fig. 7) and are replaced by signals for 2 (*ν*(CO) = 1674 cm^−1^) and ketene 8 (*ν*(CO) = 2097 cm^−1^). After 24 hours, an additional small signal is observed at 2107 cm^−1^ (Fig. S51, ESI†), proposed to be a ketenyl–iron complex. This signal reaches a maximum intensity after *ca*. 48 hours, then decays. Signals relating to Fe(CO)_5_ and 2^Mes^ are observed from the first spectra obtained after 24 hours.

**Fig. 8:**
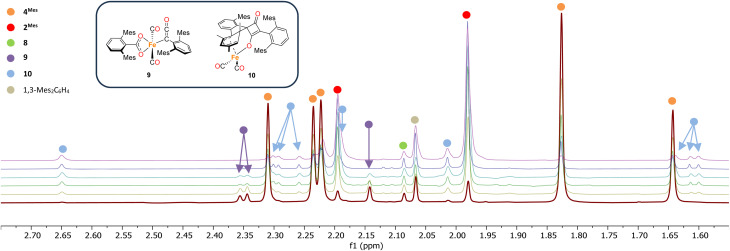
^1^H NMR spectra of the reaction of 4^Mes^ with CO in C_6_H_6_. Spectra collected on after reacting for 1 (red), 2 (yellow), 3 (green), 4 (light blue), 5 (dark blue) and 8 (purple) days. Time increases from bottom to top. Corresponding *para* methyl signals for 8 and 1,3-Mes_2_C_6_H_4_ overlap with other signals and are not shown See the ESI for full spectrum (Fig. S56)† and further details on how the data was collected. Inset, structures of proposed Fe–ketenyl intermediate 9 and cyclobutenone byproduct 10.

^1^H NMR spectroscopy was also used to investigate phase 2 of the reaction. As previously mentioned, 3^Mes^ precipitates during the reaction, hindering NMR measurements. Additionally, *in situ* NMR spectroscopic monitoring in a J. Young NMR tube leads to a different distribution of products after 4^Mes^. This is attributed to the small headspace (see Section 2.2) leading to lower CO concentrations and side reactions. To circumvent this, aliquots of a larger reaction were filtered from a J. Young reaction flask into NMR tubes at different time points and the ^1^H NMR spectrum collected (Fig. 8). Signals for 2^Mes^ are observed at *δ*_H_ = 1.98 ppm and 2.20 ppm within 24 hours of the start of the reaction. 8 was also observed (*δ*_H_ = 2.07 ppm, 2.22 ppm), which is consistent with IR spectroscopic measurements. Low intensity signals at *δ*_H_ = 2.36 ppm, 2.34 ppm and 2.14 ppm can be seen on day 2 and are consumed by day 6 (purple circles, Fig. 8). This is, again, in line with the IR spectroscopic monitoring where a ketene-type signal reaches a maximum concentration on day 3 and is subsequently consumed. Given the resolution of the peaks in the ^1^H NMR spectra, we conclude that this is likely an 18e^−^, diamagnetic complex. Complex 9 shown in Fig. 8 is the type of structure we propose that we are observing, however, this is only a tentative assignment based on the limited data and ketene containing complexes isolated (see Section 2.5). Additionally, a second diamagnetic complex, 10 (blue circles, Fig. 8) is consistently formed in small quantities. Full characterisation of 10 will be discussed further in Section 2.5.

As Fe(CO)_5_ is a product, the reaction may proceed *via* disproportionation of an Fe(i) complex. To probe the two potential pathways, EPR and Mössbauer (MB) spectroscopies were employed to gain further insight into any intermediary complexes. During the course of the EPR monitoring we observe two signals at room temperature centred at *ca*. *g*_iso_ = 2.038 and *g*_iso_ = 2.003 (Fig. S57, ESI†). The signal at *g*_iso_ = 2.003 is consistent with the previously synthesised radical anion [2]˙^−^ (Fig. S58, ESI†).^35^ The signal at *g*_iso_ = 2.038 appears as a singlet. Freezing the solution did not result in anisotropic splitting of this signal, indicating it is not due to an Fe(i) complex (Fig. S59, ESI†).

**Fig. 9:**
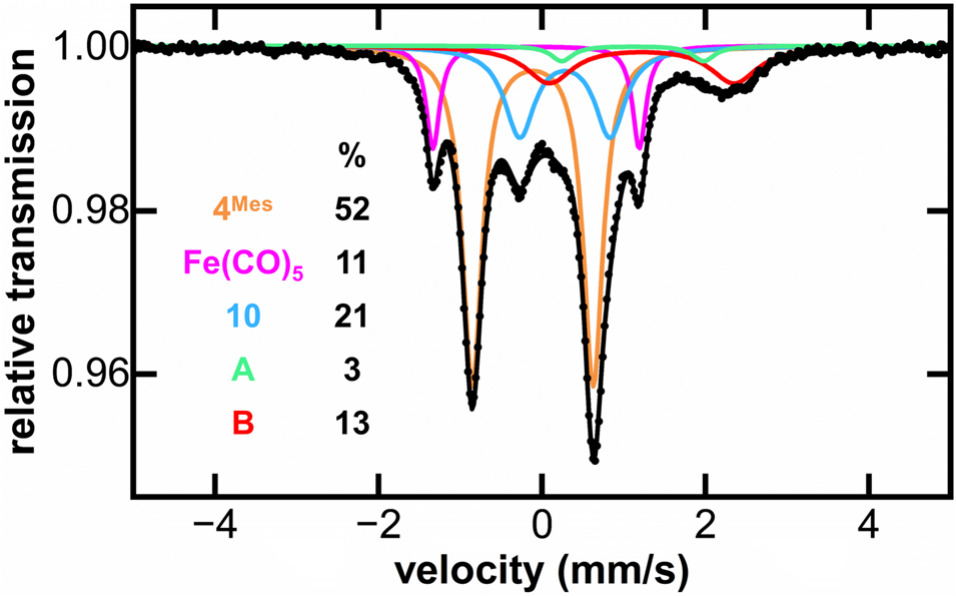
Freeze-trapped 80 K Mössbauer spectrum of the *in situ* formed iron species upon reaction of 4^Mes^ with CO at 24 hours of reaction. The individual Mössbauer components are identified as 4^Mes^ (orange. *δ* = −0.10 mm s^−1^, |Δ*E*_Q_| = 1.56 mm s^−1^) Fe(CO)_5_ (pink. *δ* = −0.08 mm s^−1^, |Δ*E*_Q_| = 2.54 mm s^−1^), 10 (light blue. *δ* = 0.30 mm s^−1^, |Δ*E*_Q_| = 1.10 mm s^−1^), A (light green. *δ* = 1.10 mm s^−1^, |Δ*E*_Q_| = 1.79 mm s^−1^) and B (red. *δ* = 1.21 mm s^−1^, |Δ*E*_Q_| = 2.33 mm s^−1^). Raw data are shown as black dots, total fit as a black line, and individual components as colored lines. Further reaction time points at 2, 3, 4 and 5 days are given in the ESI (Fig. S61).†

MB spectroscopy required a higher concentration reaction solution relative to the IR and NMR spectroscopic experiments (8-fold increase, 0.23 mol L^−1^) to enable freeze-quenched solution monitoring with natural abundance iron. Freeze-quenched MB samples of the reaction of 4^Mes^ with CO were collected over the course of the 5 days reaction. Within the first 24 h of reaction, the 80 K MB spectroscopy (Fig. 9) indicated the consumption of nearly 50% of 4^Mes^ (*δ* = −0.10 mm s^−1^, |Δ*E*_Q_| = 1.56 mm s^−1^) together with the formation of the Fe(CO)_5_ product (*δ* = −0.08 mm s^−1^, |Δ*E*_Q_| = 2.54 mm s^−1^).^42^ Note that 3 is not observed in these spectra as it is filtered away prior to collection. Over the course of the next 4 days, further generation of Fe(CO)_5_ is observed as 4^Mes^ is consumed (Fig. S59, ESI†). Three additional iron species are also observed (10, A and B) that increase over the course of the reaction and are attributed to decomposition products, one of which is 10 that is observed by ^1^H NMR spectroscopy (Fig. 8). Signals for 10, A and B are also observed when reacting 4^Mes^ in the absence of an atmosphere of CO (Fig. S85, ESI†). While the identities of A and B remain unknown, the MB parameters of 10 are consistent with a diamagnetic, low-spin iron(ii) species.^43,44^

### 2.5 Synthesis and structural characterisation of iron complexes in phase 2

**Fig. 10:**
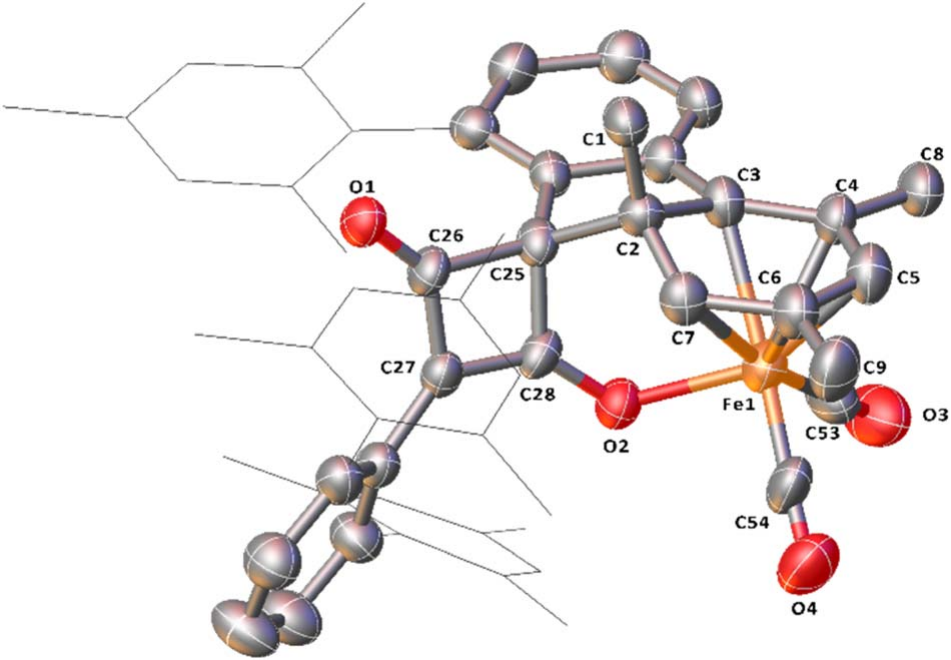
View of the metal complex from the crystal structure of 10 with anisotropic displacement ellipsoids set at 50% probability. Mesityl groups shown as wireframes and hydrogen atoms have been omitted for clarity. Selected bond distances (Å) for 10 shown: C2–C3 1.535(8), C2–C7 1.496(8), C3–C4 1.399(8), C4–C5 1.414(9), C5–C6 1.404(9), C6–C7 1.394(9), C25–C26 1.548(9), C25–C28 1.538(9), C26–C27 1.450(9), C27–C28 1.375(9), C26–O1 1.213(7), C28–O2 1.270(7), Fe1–C53 1.759(9), Fe1–C54 1.738(9), Fe1–O1 2.011(4).

Complex 10, which is commonly observed as a minor product, has been isolated as a mixture with 2^Mes^. Through *in situ* reaction monitoring, 10 was observed in larger quantities (relative to 2^Mes^) when the reaction was performed in an NMR tube. This was proposed to be due to less available CO in the reaction solution. Performing the reaction without an atmosphere of CO, greatly increases the proportion of 10. Both 10 and 2^Mes^ precipitate readily from *n*-hexane, both forming intensely orange/red crystals. Therefore, 10 has not been isolated pure for IR and NMR spectroscopic analysis. However, structural characterisation of 10 was possible by scXRD (Fig. 10). 10 contains a dearomatized mesityl ring, which is bound to an [Fe(CO)_2_]^2+^ core as a cyclohexadienyl group with the anionic O of the cyclobutenone core also bound to the Fe. The dearomatization is the result of the remaining C of the formerly aromatic cyclohexadienyl ring forming a spirocycle with the cyclobutenone core. The cyclohexadienyl bond lengths are within error of other similar complexes,^45–48^ with the delocalised C–C bonds shorter than the single C–C bonds. The metal-bound cyclobutenone contains comparable bond lengths to a free cyclobutenone synthesised by Heimgartner *et al.*^49^ The structure fits well with the ^1^H NMR signals obtained during reaction monitoring. Nine signals are obtained for the methyl groups in 10 (*δ*_H_ = 0.70 ppm, 1.60 ppm (two overlapping signals), 1.62 ppm, 2.01 ppm, 2.18 ppm 2.25 ppm, 2.30 ppm and 2.65 ppm) due to steric hindrance preventing free rotation of one of the mesityl rings. The cyclohexadienyl group also has two distinct signals at *δ*_H_ = 3.54 ppm and 4.09 ppm for the protons of the cyclohexadienyl group, which is in agreement with other similar complexes.^48,50,51^ The ^13^C{^1^H} NMR spectrum for 10 also contains a number of indicative signals between *δ*_C_ = 50–115 ppm, clearly showing the signals for the cyclohexadienyl moiety, as well as two signals for the cyclobutenone at *δ*_C_ = 89.3 ppm and 123.2 ppm. Two M–CO signals (*δ*_C_ = 204.6 ppm and 207.4 ppm) and a further two distinct C–O signals for the cyclobutenone core (*δ*_C_ = 181.7 ppm and 191.1 ppm) are also observed. These signals compare well to those reported by Heimgartner *et al.*^49^ Further confirmation for 10 comes from ^13^CO-labelling (see Section 2.6) where the signals at *δ*_C_ = 204.6 ppm and 207.4 ppm are observed as a doublet (^2^*J*_C–C_ = 15 Hz) and the four carbons of the cyclobutenone are observed as doublet-of-doublet-of-doublets (ddd, Fig. S12 and 13, ESI†). ATR-IR spectroscopic analysis of a mixture of 2 and 10 in Fomblin® gave the characteristic signal for 2 at *ν*(CO) = 1674 cm^−1^ and three remaining strong stretches. The stretches at *ν*(CO) = 2027 cm^−1^ and 1980 cm^−1^ are attributed to the metal C≡O groups and the signal at *ν*(CO) = 1732 cm^−1^ to the ketone moiety of the cyclobutenone. The cyclobutenone C=O stretch is again comparable to others in the literature.^49,52,53^

**Fig. 11:**
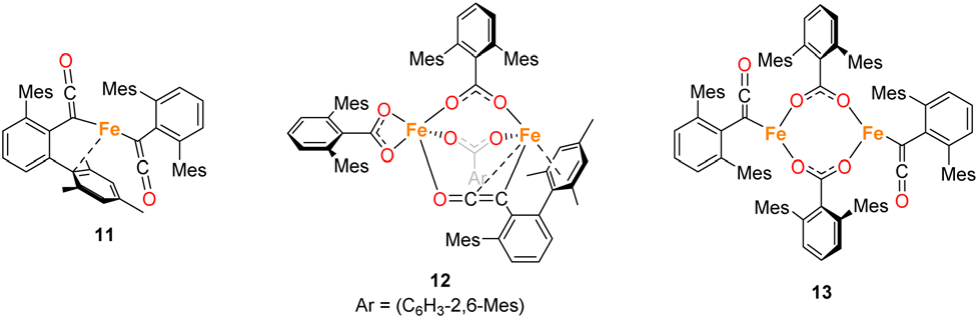
Ketene containing structures 11–13 isolated during the course of these studies.

**Fig. 12:**
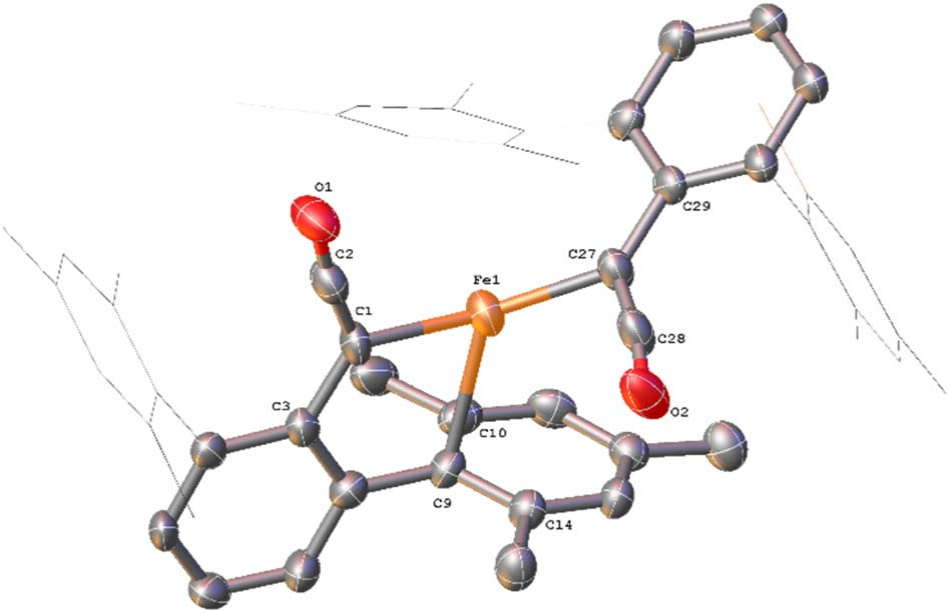
View of the metal complex from the crystal structure of 11 with anisotropic displacement ellipsoids set at 50% probability. Mesityl groups shown as wireframe, apart from the one bonding to the Fe, and hydrogen atoms have been omitted for clarity. Selected bond distances (Å) and angles (°) for 11 shown: C1–C2 1.297(10), C2–O1 1.182(9), C27–C28 1.296(10), C28–O2 1.179(9), Fe1–C1 2.052(6), Fe1–C9 2.451(7), Fe1–C10 2.843(7), Fe1–C14 2.645(7), Fe1–C27 2.039(5), C1–C2–O1 171.6(7), C1–Fe1–C27 136.0(3), C27–C28–O2 172.2(6), Fe1–C1–C2 118.9(5), Fe1–C27–C29 127.9(5).

While *in situ* IR and NMR data from phase 2 of the reaction show some evidence for iron–ketene intermediates, this is further supported by the isolation and structural characterisation of three complexes containing ketenyl moieties (Fig. 11). Complex 11 (Fig. 12) was isolated as extremely air sensitive orange crystals from a concentrated Et_2_O solution of 4^Mes^ in the absence of a CO atmosphere. Complex 11 features two ketenyl moieties bound to an iron centre, with one of the mesityl groups providing a stabilising η^3^ interaction, which shows a comparable η^3^-distance to [Zn(μ-Cl)(C_6_F_5_)(η^3^-C_6_Me_6_)]_2_ reported by Bochmann *et al.*^54^ The C=C (C1–C2, 1.297(10) Å) and [C102–C103, 1.296(10) Å] and C=O (C2–O1, 1.182(9) Å and C102–O8, 1.179(9) Å) bond distances in 11 are in accordance with the handful of iron–ketene complexes in the literature.^14,55–72^ So far, 11 has only been isolated once, and the crystallisation is challenging to replicate, so further characterisation of this species has not been possible.

**Fig. 13:**
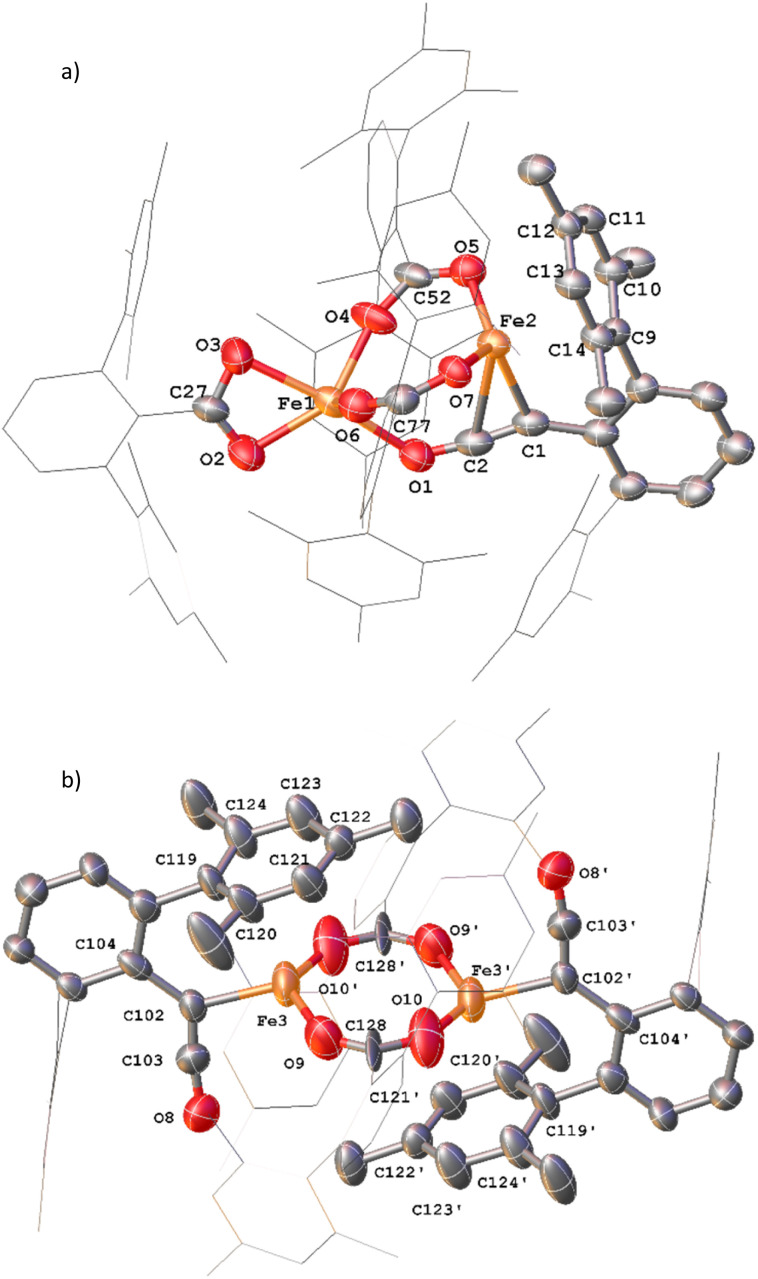
(a) View of the metal complex from the crystal structure of 12 with anisotropic displacement ellipsoids set at 50% probability. 2,6-Mes_2_C_6_H_3_ groups and non-bonding Mes groups shown as wireframe and hydrogen atoms have been omitted for clarity. Selected bond distances (Å) and angles (°) for 12 shown: C1–C2 1.254(9), C2–O1 1.233(8), Fe2–C1 2.133(6), Fe2–C2 2.475(7), Fe1–O1 2.169(5), Fe1–O2 2.104(5), Fe1–O3 2.224(5), Fe1–O4 2.016(4), Fe1–O6 2.024(5), Fe2–O5 1.987(4), Fe2–O7 1.984(4), C1–C2–O1 173.4(7), O2–C27–O3 118.1(6), O4–C52–O5 121.8(6), O6–C77–O7 124.9(6). (b) View of the metal complex from the crystal structure of 13 with anisotropic displacement ellipsoids set at 30% probability. Atoms marked with ‘were generated using the following symmetry operator: 2 − *X*, −*Y*, 1 − *Z*. 2,6-Mes_2_C_6_H_3_ groups and non-bonding Mes groups shown as wireframe and hydrogen atoms have been omitted for clarity. Selected bond distances (Å) and angles (°) for 13 shown: C102–C103 1.213(15), C103–O8 1.250(14), Fe3–C102 2.026(8), Fe3–O9 2.09(1), Fe3–O10′ 2.018(11), C102–C103–O8 172.5(12), O9–C128–O10 115.6(11), Fe3–C102–C104 126.8(7).

Complexes 12 and 13 were isolated when performing the reaction in iso-hexane in the absence of an atmosphere of CO. 12 and 13 co-crystalised in the space group *P*1̄ with one equivalent of 12 and half an equivalent of 13 per asymmetric unit (Fig. 13a). 12 is composed of one ketenyl and three carboxylate groups, with two of the carboxylates and the ketenyl moiety bridging two iron centres. The ketenyl binds to Fe2 through an η^2^-interaction with the C=C bond, and the remaining carboxylate group is bound in a terminal coordination. The ketenyl C=C (C1–C2, 1.254(9) Å) and C=O (C2–O1, 1.233(8) Å) bond lengths are similar to those in 11.

Complex 13 is situated across a special position in the asymmetric unit and contains two terminal ketenyl moieties and two bridging carboxylates (Fig. 13b). 13 shows comparable angles for the bridging carboxylates to 3 and 12, but a slight elongation of one of the Fe–O bonds [Fe3–O10, 2.09(1) Å]. For the ketene moiety in 13, the C=C bonds are shorter than the C=O, opposite to what is observed for 11 and 12. The Fe–C bond for the ketenyl group [2.026(5) Å] is also within error for a similar bond in 11.

**Fig. 14:**
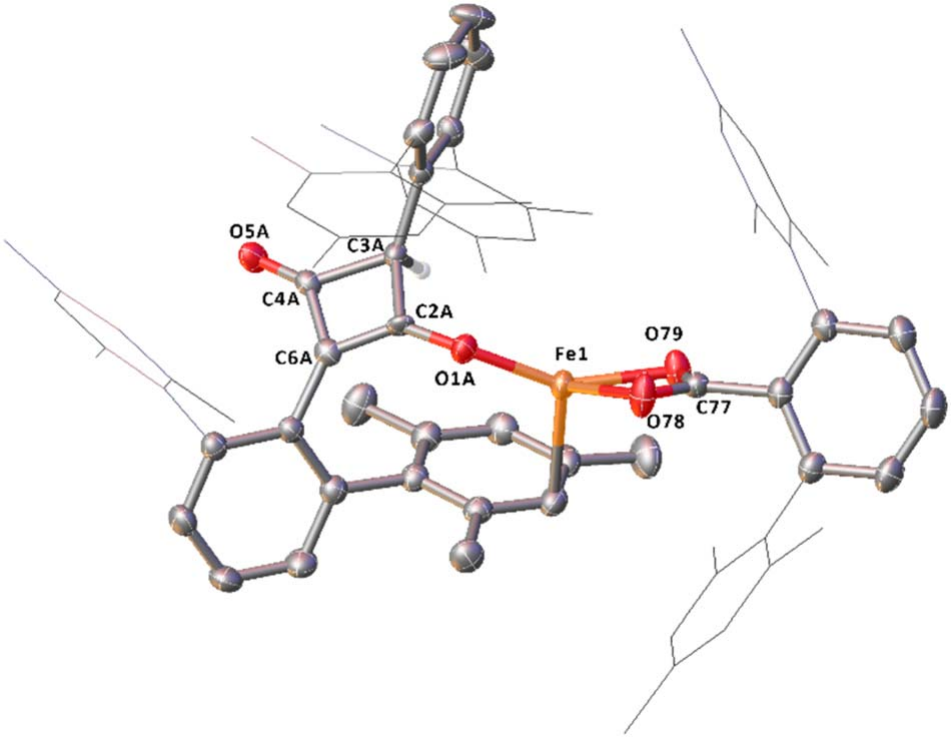
Molecular structure of 14 with anisotropic displacement ellipsoids set at 50% probability, major component of disordered C_4_ core shown. Mesityl groups shown as wireframe, except those interacting with the Fe and hydrogen atoms, except H_4_, have been omitted for clarity. Selected bond distances (Å) and angles (°) for 13 shown: Fe1–O1A 1.8702(9), Fe1–O78 2.1076(14), Fe1–O79 2.0442(10), C2A–O1A 1.306(17), C2A–C3A 1.560(17), C2A–C6A 1.362(18), C3A–C4A 1.576(13), C4A–C6A 1.456(17), C4A–O5A 1.20(2), O78–C77–O79 118.42(14).

Complex 14, which features a squaraine-like moiety bound to an Fe centre alongside a carboxylate ligand has also been identified by X-ray crystallography (Fig. 14). One of the mesityl groups of the squaraine-like moiety interacts in an η^3^ fashion to the Fe with bond lengths comparable to 11. The C_4_ moiety in 14 shows significant asymmetry as demonstrated by the differing C–C and C–O distances and is disordered by inversion of the C_4_ group. The C_4_ cycle can be best described as a cyclobutenone and is comparable to both Heimgartner's free cyclobutenone and complex 10.^49^ The carboxylate moiety is comparable to that previously reported for the terminal carboxylate of 3.

### 2.6 ^13^C labelling experiments for phase 2

**Fig. 15:**
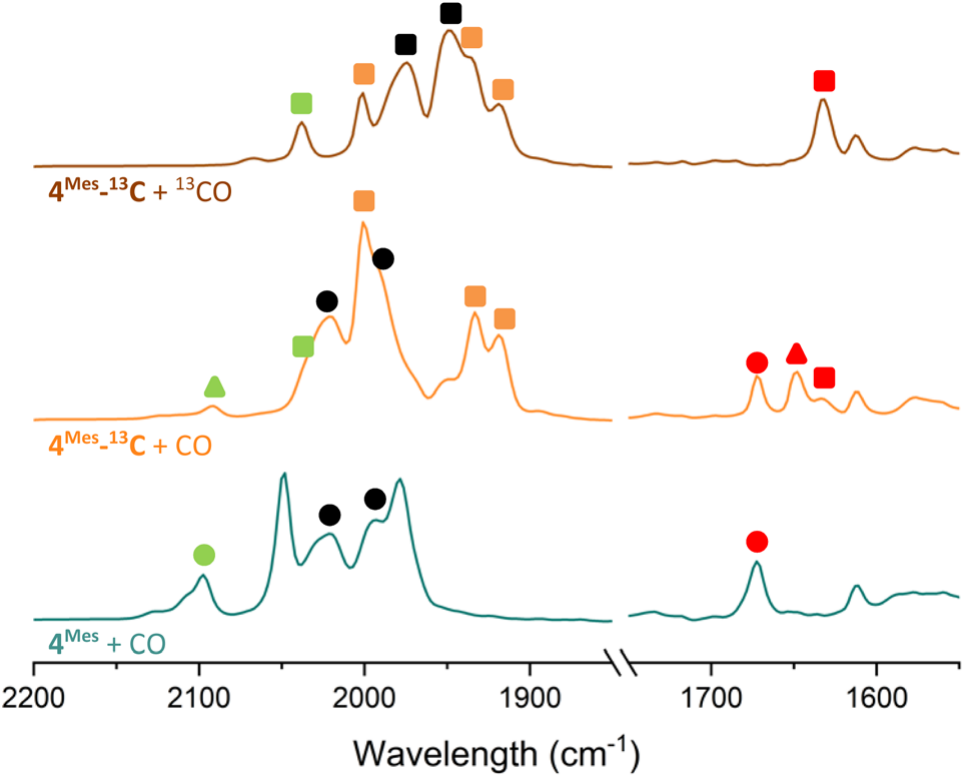
Comparison of the IR spectra of reactions after 2 days of 4^Mes^ and 4^Mes-13^C under an atmosphere of CO or ^13^CO. All reactions performed in C_6_H_6_. Left, metal carbonyl and ketene region. Right, region for squaraine. Circles signify ^12^C products, triangles signify mixed ^12^C/^13^C and square signifies ^13^C products. Signals in green are for ketene (8). Signals in red are squaraine (2^Mes^). Signals in black are Fe(CO)_5_.

To further probe the mechanism, experiments with ^13^CO were conducted. ^13^CO labelled 4^Mes^ (4^Mes-13^C) was obtained from the reaction between 1^Mes^ and ^13^CO *via* the methodology described in Section 2.3. This was then reacted further under an atmosphere of either natural abundance CO (henceforth referred to as CO) or ^13^CO. When reacting 4^Mes-13^C with ^13^CO in C_6_H_6_ (see Section 4.2.10 of the ESI for details), to allow simultaneous IR and NMR spectroscopic analysis, the IR signals are red-shifted relative to the peaks for 4^Mes^. When 4^Mes-13^C was reacted under an atmosphere of CO, the IR spectrum recorded after 48 hours showed no evidence of CO exchange for 4^Mes-13^C as the signals for this species matched those observed for 4^Mes-13^C under an atmosphere of ^13^CO (Fig. 15). However, the signals for 8 (from degradation processes) and Fe(CO)_5_ are blue-shifted relative to the spectra obtained using 4^Mes-13^C and ^13^CO, suggesting de-enrichment. The signal for 8 is marginally red-shifted for 4^Mes-13^C + CO relative to 4^Mes^ + CO (*ν*(CO) = 2091 cm^−1^*vs.* 2097 cm^−1^, 8. *Cf.*8-^13^C*ν*(CO) = 2037 cm^−1^), indicative of an Ar–^13^C = ^12^C=O (Ar = 2,6-MesC_6_H_3_) isotope pattern in 8 formed from 4^Mes-13^C + CO. Three IR signals are present for 2^Mes^ when reacting 4^Mes-13^CO under an atmosphere of natural abundance CO. This arises as the squaraine moiety has two C–O groups which can contain either ^13^C or ^12^C, and the three signals relate to squaraine C–O moieties containing ^13^C/^13^C, ^13^C/^12^C or ^12^C/^12^C. This suggests that uptake of CO is essential for the transformation of 4^Mes^ into a ketenyl-containing species, as well as for the release of Fe(CO)_5_.

**Fig. 16:**
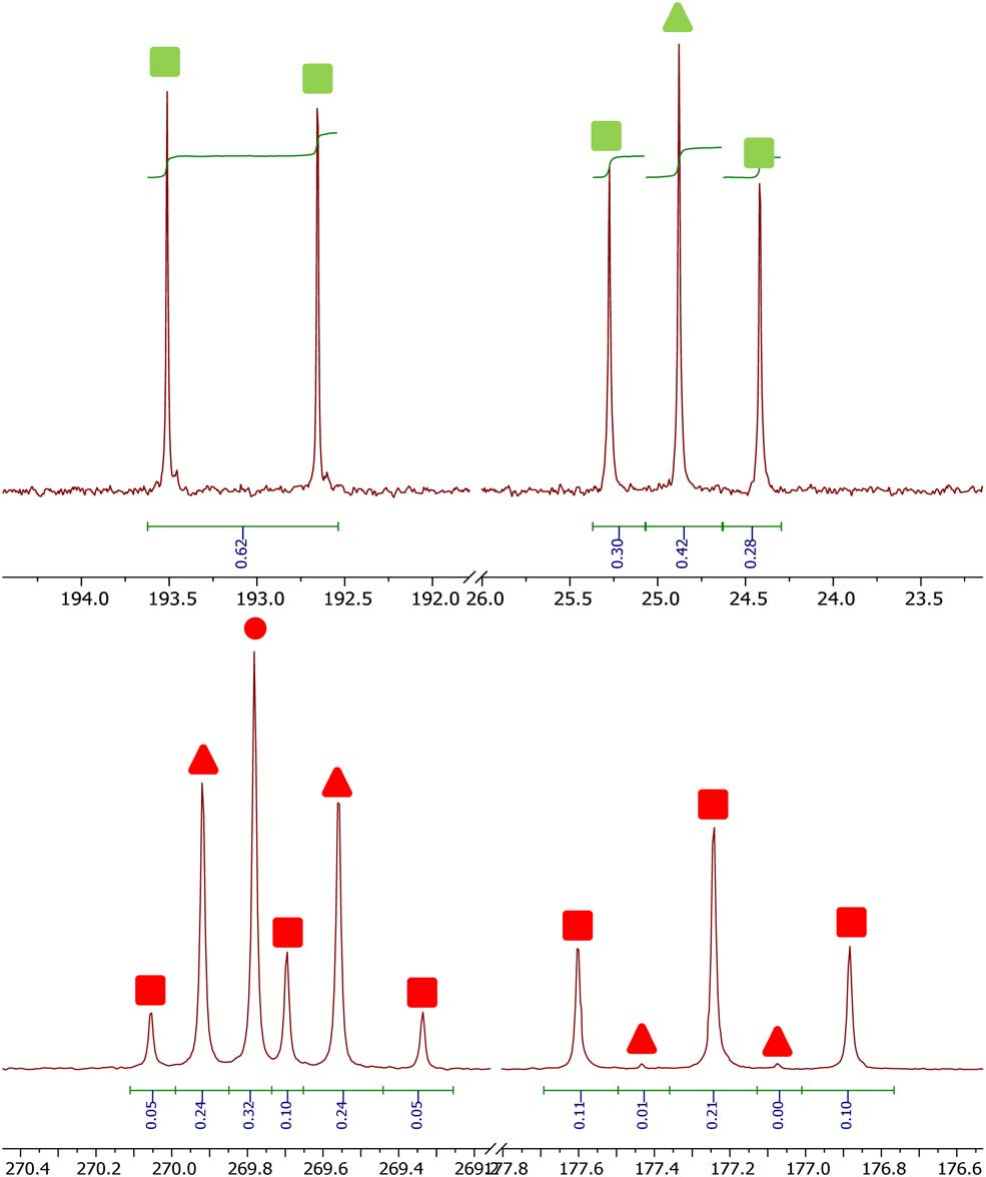
^13^C containing products from the reaction between 4^Mes-13^C and CO. Top, signals for 8. Bottom, signals for 2^Mes^. Circles signify ^12^C products, triangles signify mixed ^12^C/^13^C and square signifies ^13^C products. Markers in green are for ketene (8) and markers in red are squaraine (2^Mes^).

The ^13^C{^1^H} NMR spectra of reactions between 4^Mes-13^C and CO gives additional insight into the reaction pathway. Both compounds 8, from decomposition, and 2^Mes^ show uptake of carbon from the atmosphere, decreasing the degree of enrichment of the products (Fig. 16). For 8, the signal at 193.1 ppm is observed solely as a doublet with an integral of 0.62 while the signal at 24.8 ppm splits into a doublet and a singlet with, a total integral of 1. Taking both the coupling pattern and integrations into account, this means that the C=O of the ketene (*δ*_C_ = 193.1 ppm) is partially incorporated from the atmosphere. Conversely, the C=C=O (*δ*_C_ = 24.8 ppm) is exclusively retained, presumably the carbene carbon in 4^Mes-13^C. This highlights the importance of an atmosphere of CO in the formation of the ketenyl complex. Furthermore, performing the same analysis for 2^Mes^ indicates that there is a 56% depletion for the C=O (*δ*_C_ = 177.2 ppm, integral = 0.44) relative to the (2,6-Mes_2_C_6_H_3_)–C (*δ*_C_ = 269.8 ppm, integral = 1). This is confirmed through analysis of the coupling patterns where the (2,6-Mes_2_C_6_H_3_)–C shows a ratio of 20 (t) : 48 (d) : 32 (s), consistent with 44% of the neighbouring carbons being ^13^C enriched. For the C=O of 2^Mes^ a ratio of 98 (t) : 2 (d), consistent with 98% carbons at the neighbouring positions being enriched, which is expected as the ^13^CO used is 99% enriched. This again shows that an atmosphere of CO is essential for the reaction to progress cleanly. It also suggests that there may be more than one step where the CO is incorporated due to the difference between the enrichment of 8 and 2^Mes^.

The EPR studies (Section 2.4) indicate that radicals are present in the reactions. While one of the signals observed correlates to [2]˙^−^, the signal at *g*_iso_ = 2.038 has still not been assigned. When enriching the sample with ^13^CO, simulations of the experimental spectrum as a^13^C_4_-core using two pairs of ^13^C couplings, as is the case for [2]˙^−^, were unconvincing. A better simulation was obtained by considering lower symmetry in a ^13^C_4_-core with one larger coupling on a single ^13^C environment and a smaller coupling across three ^13^C atoms (Fig. S68, 69 and Table S3, ESI†). This splitting is consistent with that expected for a radical centered on a species similar to a squaraine where the C_4_ core bears both a C=O and C–O group with delocalisation of the radical over three carbon atoms. For reactions between CO and 4^Mes-13^C, the EPR signal obtained for [2]˙^−^ shows depletion of the ^13^C for the C=O, and the signal was modelled with a 43% ^13^C enrichment. This is consistent that observed in the ^13^C{^1^H} NMR spectra. The EPR studies show that the environment in which the reaction proceeds is highly reducing, with [2]˙^−^ forming in the absence of an external reductant. Thus, single electron reactions are highly plausible.

### 2.7 Proposed mechanism for phase 2

**Scheme 4:**
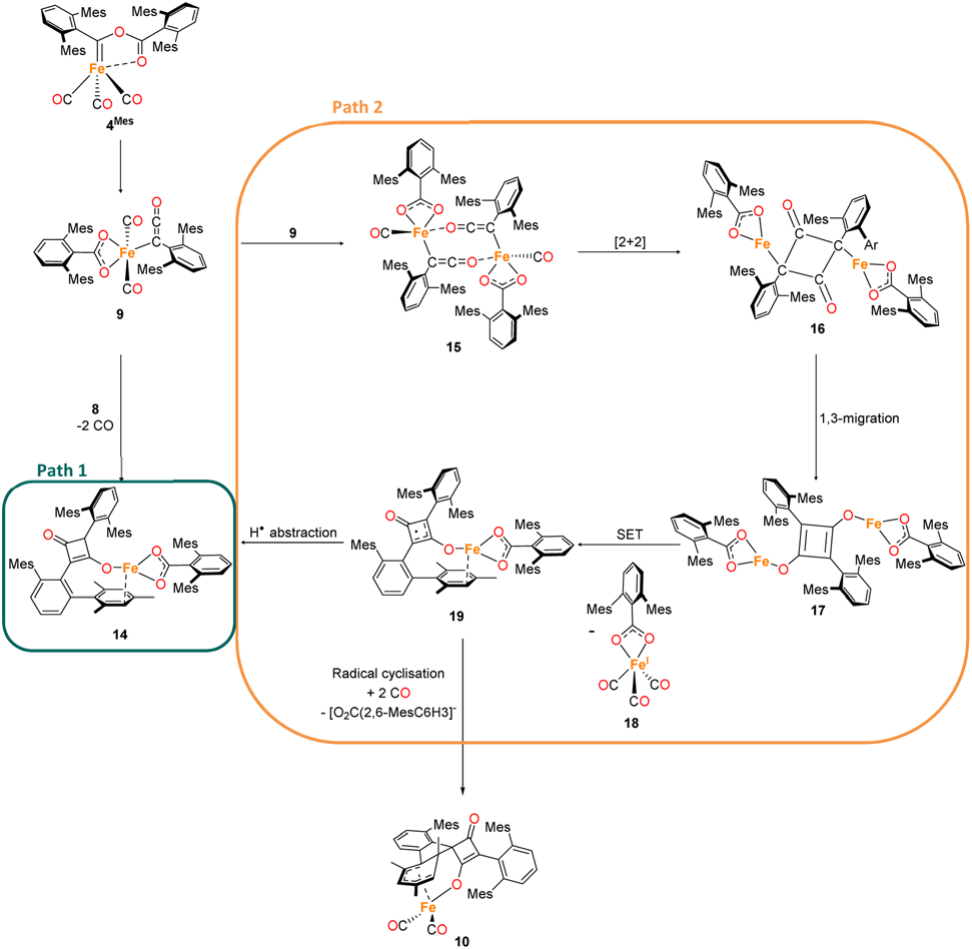
Two potential routes to 14*via* proposed complex 9. Path 1 forms 14*via* a [2 + 2] cycloaddition of 8 and 9. Path 2 forms 14 through dimerization of 9 then a [2 + 2] cyclisation, followed by 1,3 migration and a single electron transfer, giving 18 and 19. Path 2 also has the potential to form decomposition complex 10*via* radical cyclisation of 19.

The mechanism for phase 2 of the reaction (4^Mes^ to 2^Mes^, 3^Mes^, Fe(CO)_5_) is more tentative than phase 1, but we propose a plausible route based on the data available. Some key observations are that ketene 8 and an iron–ketene complex (9) are observed spectroscopically (*in situ* IR and NMR) and that three iron–ketene containing byproducts (11–13) were isolated from the reaction. Thus, an iron–ketene complex is likely a key intermediate. We must also account for the formation of cyclobutenones 10 and 14 (Fig. 10 and 14), which are structurally related to squaraine 2^Mes^. We therefore propose that 4^Mes^ initially rearranges to an iron ketene/carboxylate 9 (Scheme 4). While 9 has not been definitively characterised, its formation is consistent with the observed data, and we tentatively assign the iron–ketene signals (Fig. 7 and 8) to 9.

From 9, two possible paths to the isolated cyclobutanone complex 14 can be envisaged (Scheme 4). Path 1 involves a [2 + 2] cycloaddition between 9 and 8, followed by a 1,3-migration of the Fe centre, leading directly to 14. However, this route would not explain the presence of radical species detected by EPR spectroscopy. We therefore suggest that Path 2, which involves Single Electron Transfer (SET) processes, is more plausible (Scheme 4). In path 2, two molecules of 9 dimerise to form 15. 15 then undergoes a intramolecular [2 + 2] cycloaddition to form 16 followed by two 1,3-migrations to form 17, reducing steric crowding. From 17, an Fe(i) complex (18) and an Fe complex with a ligand centred radical (19) are formed by SET. Cyclobutenone complexes 10 and 14 can both be formed from the proposed 19, either by radical cyclisation (10) or H-atom abstraction (14).

**Scheme 5:**
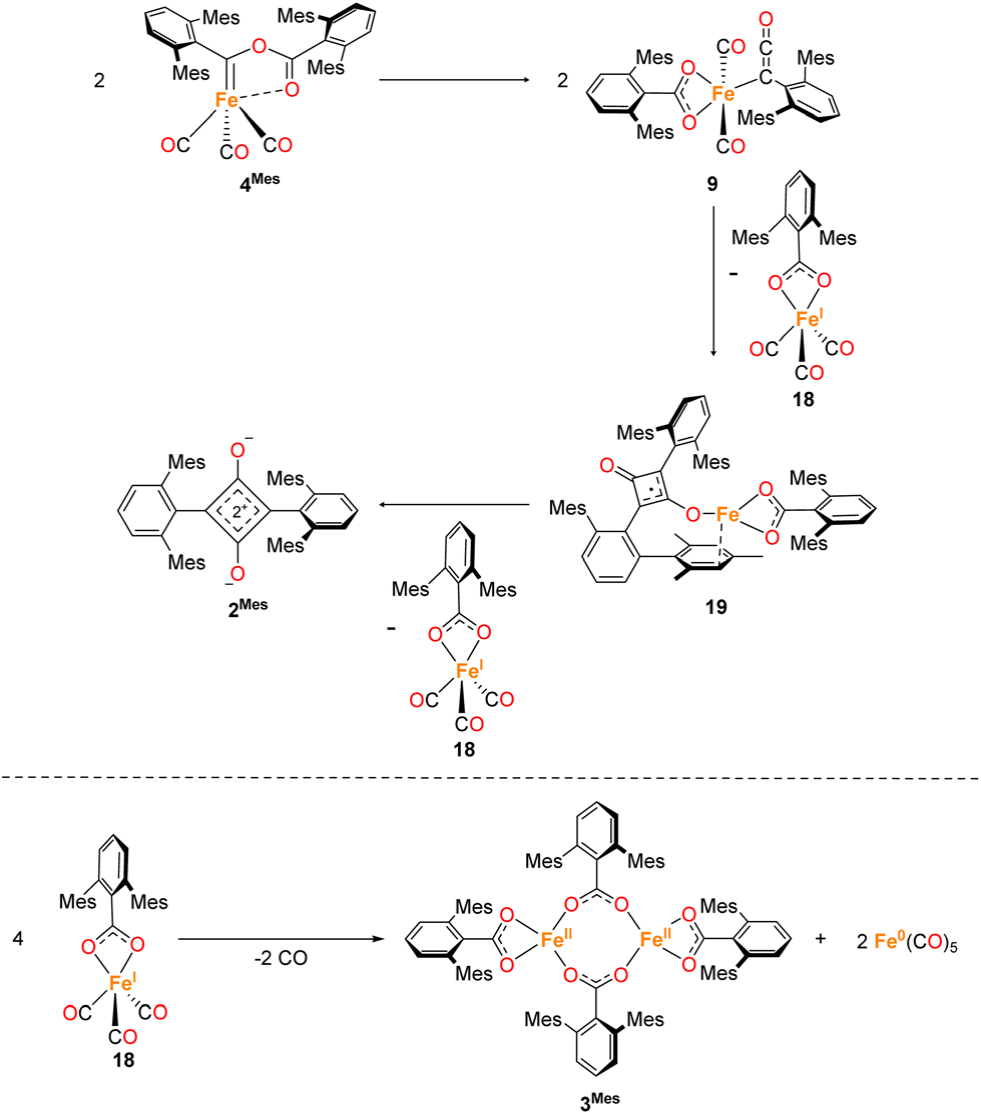
Proposed formation of 2^Mes^ and 13*via* a ketenyl complex (9) by sequential single electron transfers, forming Fe(i) complex 18. 18 then undergoes disproportionation resulting in 3^Mes^ and Fe(CO)_5_.

From this, we propose the overall mechanism shown in Scheme 5. Here, the reaction proceeds as in Scheme 4 up to the formation of the ligand-centred radical 19. This undergoes an additional SET to form the squaraine 2^Mes^, along with another equivalent of Fe(i) species 18. We then propose that 18 undergoes disproportionation to form the other major products, iron carboxylate 3^Mes^ and Fe(CO)_5_. Since it was not possible to observe Fe(i) signals by EPR or MB spectroscopy, we suggest the disproportionation of 18 is rapid. This is consistent with the formation of Fe(CO)_5_ in solution, and precipitation of 3^Mes^ within 24 hours. Excess CO is required for clean formation of Fe(CO)_5_, otherwise the reactive intermediates undergo alternative reactions, resulting in cyclobutenone 10 and the unknown Fe complexes detected by MB spectroscopy (A and B, Fig. 9). It is worth noting that, A and B are always observed by MB spectroscopy due to the high concentrations required for this technique, meaning that it is not possible for excess CO in solution to react for 4^Mes^ to react cleanly.

Further DFT studies for phase 2 of the reaction proved difficult due to the size of the dimer like molecules. After the formation of 4^Mes^, high-spin Fe is strongly preferred according to the calculations. This is consistent with the observed formation of paramagnetic species in the ^1^H NMR spectra (see Fig. S61†).

## 3. Conclusions

Through combined spectroscopic and structural studies we have developed a plausible mechanism for the formation of a squaraine (2^Mes^) from two-coordinate 1^Mes^, *via* reaction with CO. The first part of reaction clearly shows the formation of diamagnetic complexes by ^1^H NMR spectroscopy and a number of carbonyl containing complexes with complexes 5–7 isolated en route to the stable complex 4^Mes^, which are envisaged to form though sequential migratory insertion reactions. The reactivity post formation of 4^Mes^ is significantly more difficult to follow due to the high sensitivity and reactivity of the intermediate species. Our initial studies proposed that Fe–ketenyl complexes were responsible for the formation of 2 due to a characteristic signal at 2097 cm^−1^ in the IR spectrum, however, this signal was found to relate to a protonated ketene (8) which forms in the presence of silicon grease and/or moisture. Other signals which are proposed to relate to Fe–ketenyl complexes are fleetingly observed by IR spectroscopy, but support them being key intermediates to the formation of 2. Pleasingly, other ketene complexes were isolated over the course of these studies (11–13) which gives strong evidence that ketenyl complexes are present en route to 2. However, these are proposed to be decomposition products and not active in the formation of 2. Complexes 10 and 14 both contain ligands bearing cyclobutenone moieties, providing further clues on potential intermediates in this reaction, all of which are derived from ketenes/ketenyl groups. Further spectroscopic studies using labelled materials and performing the reaction in the absence of an atmosphere of CO highlight the importance of excess CO in the reaction. Reactions of labelled 4^Mes-13^C under natural abundance showed selective loss of ^13^CO from the C–O of the squaraine product indicating that key steps post 4^Mes^ forming ketenes requires uptake of CO from solution. The enriched carbene C in 4^Mes-13^C is retained adjacent to the terphenyl moiety in 2^Mes^. EPR spectra at room temperature showed evidence for the formation of radicals which are consumed over the course of the reaction, however, they could not be unequivocally identified. Mössbauer spectroscopy showed that the iron intermediates post 4^Mes^ react rapidly, forming Fe(CO)_5_ within 24 hours. While there is no direct evidence for an Fe(i) complex by EPR and MB spectroscopy, it is possible to propose a series of SET reactions which form a fleeting Fe(i) complex (18) that undergoes rapid disproportionation to Fe(CO)_5_ and 3^Mes^. This work demonstrates how a multi-spectroscopic and structural approach is needed to truly understand highly complex reactions, showcasing the benefits of both *in situ* measurements and reaction sampling methodology.

## Author contributions

NTC: drafted the manuscript. DLK, JM, NTC and LJT: conceptualization and supervision of the project. NTC, LJT, NC, YL, DR, MLN and DLK: writing, reviewing and editing of the manuscript. NTC, DR, NC: compiled the ESI. DR, AB, LJT: performed DFT analysis. YL, NTC, SPA: collected XRD data in house, solved and refined the crystal structures. NTC and SPA: performed XRD data collections at Diamond Light Source and validated the refined XRD data. ESD: collection and processing of EPR data. PJM: assistance with ReactIR methodology and setup. KB: implemented bespoke and high resolution NMR data collection. BP-G: performed MALDI mass spectrometry. MLN, NC: performed Mössbauer measurements and analysis. NTC, YL, NC: contributed to the experimental work, formal analysis and data curation.

## Conflicts of interest

There are no conflicts to declare.

## Supplementary Material

SC-015-D4SC01286K-s001

SC-015-D4SC01286K-s002

SC-015-D4SC01286K-s003

SC-015-D4SC01286K-s004

SC-015-D4SC01286K-s005

SC-015-D4SC01286K-s006

SC-015-D4SC01286K-s007

SC-015-D4SC01286K-s008

SC-015-D4SC01286K-s009

SC-015-D4SC01286K-s010

SC-015-D4SC01286K-s011

SC-015-D4SC01286K-s012

SC-015-D4SC01286K-s013

SC-015-D4SC01286K-s014

SC-015-D4SC01286K-s015

## Data Availability

A data repository containing spectroscopic data in their raw (IR and NMR) and processed forms (mass spectrometry, CHN, EPR and Mössbauer spectroscopy) can be found *via* the following https://doi.org/10.17639/nott.7407.
