# Supplementary material for: Mechanistic investigations of the Fe(ii) mediated synthesis of squaraines
Source: Chem Sci. 2024 May 7;15(25):9599–611. doi: 10.1039/d4sc01286k (PMC11206310; doi:10.1039/d4sc01286k)
Supplement: SC-015-D4SC01286K-s006 [file SC-015-D4SC01286K-s006.pdf]

Electronic Supplementary Material (ESI) for Chemical Science.
This journal is © The Royal Society of Chemistry 2024

Supporting Information for:

Mechanistic investigations of the Fe(II) mediated synthesis of squaraines

Yu Liu, Nathan T. Coles, Nathalia Cajiao, Laurence J. Taylor, E. Stephen Davies, Alistair Barbour, Patrick J. Morgan, Kevin Butler, Ben Pointer-Gleadhill, Stephen P. Argent, Jonathan McMaster, Michael L. Neidig, David Robinson and Deborah L. Kays

Contents

1. General Procedures 4

2. Synthesis of compounds and characterisation 5

2.1 General synthesis 5

2.1.1 Synthesis of intermediate complexes 5

2.1.2 Characterisation of decomposition compounds 8

2.2 NMR spectra 10

2.3 IR spectra 16

2.4 Mass spectra 22

3. Crystallographic information 27

3.1 Additional refinement details 28

3.1.1 Further refinement details for 6 28

3.1.2 Further refinement details for 7 29

3.1.3 Further refinement details for 10 29

3.1.4 Further refinement details for 11 29

3.1.5 Further refinement details for 12/13 29

3.1.6 Further refinement details for 14 30

3.1.7 Further refinement details for $3^{Mes}\cdot$*i*Hex 30

3.1.8 Further refinement details for $3^{Mes}\cdot(H_2O)_4$ 30

3.2 $4^{Mes}\cdot Et_2O$ 31

3.3 Squaraine (2^{Mes}) solvatomorphs 32

3.3.1 No solvent (from *i*-hexane) 32

3.3.2 No solvent (from Et_2O) 32

3.3.3 C_6H_6 solvate 33

3.4 Carboxylate (3^{Mes}) solvatomorphs/derivatives 33

3.4.1 *iso*-Hexane solvatomorph 33

3.4.2 Cyclohexane solvatomorph 34

3.4.3 Water complex of 3^{Mes} 34

4. Reaction monitoring details 35

4.1 Pre carbene formation 35

4.1.1 ReactIR monitoring 35

4.1.2 *In situ* ^1H NMR monitoring 36

4.1.3 *In situ* $^{13}C\{^1H\}$ NMR monitoring 37

4.2 Post carbene formation 39

4.2.1 End of reaction starting from 1^{Mes} 39

4.2.2 End of reaction starting from 4^{Mes} 39

4.2.3 IR spectra by reaction sampling, toluene 40
4.2.4 IR spectra by reaction sampling, cyclohexane 41
4.2.5 NMR spectra by reaction sampling 42
4.2.6 *In situ* monitoring by EPR spectra 44
4.2.6 Frozen EPR spectra 44
4.2.7 Mössbauer spectroscopy characterisation 46
4.2.8 Reaction monitoring *via* Mössbauer spectroscopy 46
4.2.9 Simultaneous EPR, IR and NMR sampling 48
4.2.10 ^{1}H and ^{13}C{^{1}H} NMR, EPR and IR spectra of 4^{Mes}-^{13}C under ^{13}CO 50
4.2.11 ^{1}H and ^{13}C{^{1}H} NMR, EPR and IR spectra of 4^{Mes}-^{13}C under natural abundance CO 54
4.2.12 ^{1}H, ^{13}C{^{1}H} NMR and Mössbauer spectrum of 4^{Mes} after one month in the absence of CO 57
4.3 EPR fitting parameters 59
5. Computational Details 60
6. References 60

1. General Procedures

All products described were synthesised with rigorous exclusion of air and water using standard air-sensitive-handling techniques which included bench-top operations (Schlenk line) and glovebox techniques, under argon and nitrogen, respectively. Solvents (*iso*-hexane, diethyl ether, toluene, THF) were collected from in-house dry-solvent towers, degassed and stored over activated molecular sieves. C_6D_6 was dried over activated molecular sieves for 1 week, filtered, degassed and placed onto a second batch of activated molecular sieves and stored in the glovebox prior to use. $Fe(C_6H_3\text{-}2,6\text{-}Mes_2)_2$ (**1**^{Mes}) was synthesised according to the literature procedure.[1] **2**^{Mes} and **3**^{Mes} were previously isolated and the data obtained matches the literature.[2]

Air and moisture sensitive samples for NMR spectroscopy were prepared using glovebox techniques and sealed in J Young´s tap modified borosilicate glass NMR tubes. NMR spectra were collected on either a Bruker AV400, AV(III)400, AV(III)400HD or AV(III)500 spectrometer at 298 K, unless otherwise stated. Chemical shifts are quoted in ppm relative to residual protic solvent (^1H, $^{13}C\{^1H\}$).

Elemental microanalyses were performed on a Exeter Analytical CE-440 Elemental Analyzer with samples combusted at temperatures of 975 °C before being measured.

Carbonyl complexes **4**^{Mes}, **4**^{Mes}**-**^{13}**CO** and **6** were analysed by matrix-assisted laser desorption ionisation (MALDI) mass spectrometry using a Bruker Autoflex Max. Samples were prepared by addition of 100 µL of acetonitrile to 1 mg of dry material, the resulting suspension combined at a ratio of 1:2 with a 20 mg/mL solution of trans-2-[3-(4-*tert*-butylphenyl)-2-methyl-2-propenylidene]malononitrile (DCTB)[3] in acetonitrile. 0.5 µL of the resulting mixture was spotted onto the sample plate for analysis. The analysis was calibrated by collection of a spectrum of polyethylene glycol alongside the sample spectra.

In-situ IR measurements were performed using a Mettler Toledo ReactIR 15 fitted with a silicon probe. Spectra were collected using the high-resolution regime (4 cm^{-1} resolution) collecting a spectral average of 256 scans, with spectra collected at no more than every 2 minutes. Experiment control and data analysis performed with Mettler Toledo iCIR 7.1 software. ATR-IR of solid samples were collected using a Bruker Alpha using a resolution of 2 cm^{-1}, a frequency range of 500-4000 cm^{-1} and a spectral average of 32 scans. These spectra were collected in air unless otherwise stated. A background of the atmosphere was taken before each data collection. IR of solutions by reaction sampling were performed using a Harrick cell fitted with CaF_2 windows and 100 µm Teflon spacers. The solutions were transferred in the glovebox to the cell and sealed under an inert atmosphere. The spectra were collected on a Bruker Alpha at a resolution of 4 cm^{-1} and a frequency range of 500-4000 cm^{-1} across an average of 32 scans. A background of air was taken before each data collection. To obtain the sample spectra, a spectrum of CaF_2 and toluene was collected which was then subtracted from the sample spectra to give the peaks corresponding to the iron CO containing species. High-resolution solution IR spectra were collected on a Bruker Vertex 70 at a resolution of 0.5 cm^{-1} and a frequency range of 6000-400 cm^{-1}. The sample chamber was purged with dinitrogen for a minimum of 15 minutes prior to use. A single channel spectrum of the solvent/CaF_2 windows with 100 µm Teflon spacers was collected in advance of the experiments and used as the background. To background, a single channel spectrum of the sample was collected and the sample spectrum was ratioed against the solvent/CaF_2 spectrum using the spectrum calculator function within the OPUS software. This was to have a consistent background across all samples.

Continuous wave X-band EPR spectra were performed in a quartz EPR tube with a Schlenk adapter or a standard EPR tube sealed in a glovebox and further sealed with electrical tape on a Bruker EMX spectrometer as a solution at room temperature or a frozen toluene solution at 77K. The simulations of CW EPR spectra were performed using the Bruker WINEPR SimFonia package.

Low-temperature ^{57}Fe Mössbauer measurements were performed using a See Co. MS4 Mössbauer spectrometer integrated with a Janis SVT-400T N_2 cryostat for measurements at 80 K. Isomer shifts were determined relative to α-Fe at 298 K. All Mössbauer spectra were fit using the program WMoss (SeeCo). Errors of the fit analyses were the following: $\delta \pm 0.02$ mm/s and $\Delta EQ \pm 3\%$. For multicomponent fits, the quantitation errors of individual components were ±3%.

Suitable single crystals of **4^{Mes}**, **$4^{Mes}\cdot Et_2O$**, **5**, **6**, **7**, **10**, **11**, **12/13**, **14**, **2·nosolv1**, **2·nosolv2**, **$2\cdot C_6H_6$**, **3·*i*Hex**, **3·CyH** and **$3\cdot(H_2O)_4$**, were selected under the protection of Fomblin® (YR-1800 perfluoropolyether oil) then mounted on a polymer-tipped MicroMount™ and rapidly cooled with a stream of N_2 at 120 K (in house devices) or 100 K at Diamond Light Source. The data were collected on an Oxford Diffraction GV1000 (TitanS2 CCD area detector, mirror-monochromated Cu-Kα radiation source; λ = 1.54184 Å, ω scans, [**4^{Mes}**, **6, 7**, **3·CyH**]), an Oxford Diffraction GV1000 (AtlasS2 CCD area detector, mirror-monochromated Cu-Kα radiation source; λ = 1.54184 Å, ω scans, [**13**]), an Oxford Diffraction SuperNova Duo (Atlas CCD area detector, mirror-monochromated Mo-Kα radiation source; λ = 0.71073 Å; ω scans, [**5**]) a Rigaku XtaLAB PROMM007 (PILATUS3 R 200K Hybrid Pixel Array detector, mirror-monochromated Cu-Kα radiation source; λ = 1.54184 Å, ω scans, [**11**, **12/13**, **2·nosolv2**, **$2\cdot C_6H_6$**, **3·*i*Hex**, **$3\cdot(H_2O)_4$**]) or in Experimental Hutch 1 (EH1) on beamline I19 at Diamond Light Source[4] on a Fluid Film Devices 3-circle fixed-chi diffractometer (Dectris PILATUS 2M Detector, wavelength 0.6889 Å, [**$4^{Mes}\cdot Et_2O$**, **10**, **2·nosolv1**]). Data collection was handled by either CrysAlisPro[5] (Rigaku XtaLAB) or GDA (Diamond Light Source). The collected frames were integrated using either CrysAlisPro (Rigaku XtaLAB) or XIA26 software[6] (Diamond Light Source) and the data were corrected for absorption effects using a Gaussian numerical method with beam profile correction (CrysAlisPro) or AIMLESS[7] an empirical method (Diamond Light Source). Structures were solved within Olex2[8] by dual space iterative methods (SHELXT)[9] and all non-hydrogen atoms refined by full-matrix least-squares on all unique F2 values with anisotropic displacement parameters (SHELXL).[10] Hydrogen atoms were refined with constrained geometries and riding thermal parameters. Structures were checked with checkCIF.[11]

2. Synthesis of compounds and characterisation

2.1 General synthesis

2.1.1 Synthesis of intermediate complexes

4^{Mes} - $Fe(CO)_3[C(2,6\text{-}Mes_2C_6H_3)OC(O)(2,6\text{-}Mes_2C_6H_3)]$

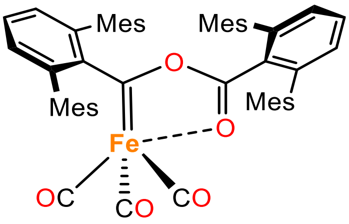

1^{Mes} (1.2 g, 1.8 mmol) was suspended in *i*-hexane (70 mL). The flask was sealed securely and placed under a partial vacuum to remove the argon from the headspace. The flask was backfilled with *ca*. 1

atm of CO, resulting in a colour change of the solution from yellow to orange. The reaction initially formed a pale orange precipitate which redissolved over the course of the reaction. Periodically (every 5 minutes) the reaction vessel was topped up with CO as it was consumed. The reaction was deemed to be complete once the orange precipitate has disappeared and a further 10 minutes had passed (taking ~45 mins overall). Once finished, the reaction was filtered through a cannula fitted with a glass fibre filter. The solvent was removed *in vacuo* resulting in a flocculant orange solid. Yield = 1.14 g (1.4 mmol, 77 %).

To obtain high purity **4^{Mes}** and crystals suitable for X-ray diffractometry, the above solid was dissolved in the minimum quantity of Et_2O and placed in a freezer at −30 °C. Yield after recrystallisation = 0.92 g (1.1 mmol, 61 %). **Note:** Two solvatomorphs of **4^{Mes}** have been isolated from Et_2O (see crystallography).

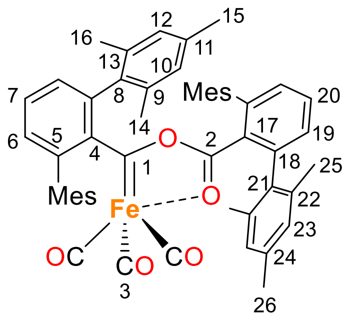

^{1}H NMR (500 MHz, C_6D_6): δ 7.00 (t, $^3J_{H-H}$ = 7.6 Hz, 1H, *H*7), 6.95 (t, $^3J_{H-H}$ = 7.6 Hz, 1H, *H*20), 6.87 (s, 2H, *H*12), 6.82 (d, $^3J_{H-H}$ = 7.6 Hz, 2H, *H*6), 6.81 (s, 4H, *H*23), 6.73 (s, 2H, *H*10), 6.69 (d, $^3J_{H-H}$ = 7.6 Hz, 2H, *H*19), 2.30 (s, 6H, *H*16), 2.23 (s, 6H, *H*15), 2.22 (s, 6H, *H*26), 1.82 (s, 12H, *H*25), 1.64 (s, 6H, *H*14) ppm.
^{13}C{^{1}H} NMR (126 MHz, C_6D_6): δ 261.0 (*C*1), 211.3 (*C*3), 175.7 (*C*2), 148.1 (*C*4), 143.0 (*C*17), 139.6 (*C*5), 139.2 (*C*8), 137.5 (*C*18/21), 137.2 (*C*9), 136.4 (*C*11), 136.1 (*C*13), 135.9 (*C*22), 131.6 (*C*20), 130.7 (*C*19), 130.5 (*C*6), 129.0 (*C*12/23), 128.13 (*C*10), 128 .04 (*C*7), 21.8 (*C*14), 21.3-21.1 (br., *C*15/16/26), 20.9 (*C*25)). C7 and C10 are obscured by the signal for C_6D_6 and were determined from the HSQC/HMBC spectra.
ReactIR (C_6H_6, Silicon probe), $\nu_{(CO)}$ (cm^{-1}): 2048 (CO), 1978 (CO), 1963 (CO), 1612 (carboxyl).
ReactIR (Toluene, Silicon probe), $\nu_{(CO)}$ (cm^{-1}): 2049 (CO), 1978 (CO), 1963 (CO), 1612 (carboxyl).
Harrick cell (Toluene, CaF_2): $\nu_{(CO)}$ (cm^{-1}): 2050, 1978, 1962 (CO).
Harrick cell (Cyclohexane, CaF_2): $\nu_{(CO)}$ (cm^{-1}): 2051, 1981, 1967 (CO).
ATR-IR: $\nu_{(CO)}$ (cm^{-1}): 2046 (CO), 1974 (CO), 1959 (CO), 1612 (carboxyl).
HRMS (MALDI-TOF) *m/z*: [M-2(CO)]$^+$, calculated 766.3104, found 766.3121, err. = −2.1 ppm. [M-3(CO)]$^+$, calculated 738.3155, found 738.3193, err. = −5.0 ppm.
Elemental analysis: Calculated: C, 77.37; H, 6.13. Found: C, 77.09; H, 5.96.

4^{Mes}-^{13}C – Fe(^{13}CO)$_3$[^{13}C(2,6-$Mes_2C_6H_3$)O^{13}C(O)(2,6-$Mes_2C_6H_3$)]

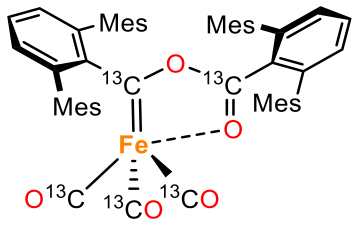

1^{Mes} (530 mg, 0.78 mmol) was suspended in *i*-hexane (10 mL). The flask was sealed securely and placed under a partial vacuum to remove the argon from the headspace. The flask was backfilled with *ca.* 1 atm of ^{13}CO, resulting in a colour change of the solution from yellow to orange. The reaction initially formed a pale orange precipitate which redissolved over the course of the reaction. The flask was refilled with ^{13}CO every 5 minutes to maintain a pressure of ~1 atm. The reaction was deemed to be complete once the orange precipitate had disappeared and a further 10 minutes had passed (taking 45 mins overall). Once finished, the reaction was filtered through a Teflon cannula fitted with a glass fibre filter. The solvent was removed *in vacuo* resulting in a flocculant orange solid. The obtained solid was recrystallised from a concentrated Et_2O solution at −30 °C. 190 mg (0.23 mmol, 29 %). A further 240 mg (0.29 mmol, 37%) was isolated which contained traces of 2,6-$Mes_2C_6H_4$ (~10 % relative to **4**^{Mes}**-**^{13}**C**).

^{1}H NMR (500 MHz, C_6D_6): δ 7.01 (t, $^3J_{H\text{-}H}$ = 7.5 Hz, 1H, *H*7), 6.96 (t, $^3J_{H\text{-}H}$ = 7.6 Hz, 1H, *H*20), 6.87 (s, 2H, *H*12), 6.82 (overlapping d, *H*6), 6.82 (s, 4H, *H*23), 6.73 (s, 2H, *H*10), 6.70 (d, $^3J_{H\text{-}H}$ = 7.6 Hz, 2H, *H*19), 2.31 (s, 6H, *H*16), 2.24 (s, 6H, *H*15), 2.22 (s, 6H *H*26), 1.83 (s, 12H, *H*25), 1.64 (s, 6H, *H*14) ppm.
^{13}C{^{1}H} NMR (101 MHz, C_6D_6): δ 261.0 (*C*1), 211.2 (*C*3), 175.7 (*C*2), 148.1 (dd, *J* = 47 Hz, 5 Hz, *C*4), 143.0 (d, *J* = 2 Hz, *C*17), 139.6 (*C*5), 139.2 (*C*8), 137.5 (*C*18/21), 137.2 (*C*9), 136.4 (*C*11), 136.1 (*C*13), 135.9 (*C*22), 131.6 (*C*20), 130.7 (d, *J* = 4Hz, *C19*), 130.5 (d, *J* = 3 Hz, *C*6), 129.0 (*C*12), 128.9 (*C*23), 128.13 (*C*10), 128 .04 (*C*7), 21.8 (*C*14), 21.3 (*C*15), 21.2 (*C*16), 21.1, (*C*26) 20.9 (*C*25). C7 and C10 are obscured by the signal for C_6D_6 and were determined from the HSQC/HMBC spectra.
Harrick cell (C_6H_6, CaF_2): $\nu_{(CO)}$ (cm^{-1}): 2001, 1937, 1919 (CO).
ATR-IR: $\nu_{(CO)}$ (cm^{-1}): 1998 (CO), 1930 (CO), 1912 (CO), 1611 (carboxyl).
HRMS (MALDI-TOF) *m/z*: [M–3(^{13}CO)]$^+$, calculated 740.3223, found 740.3259, err. = –4.9 ppm.

6 – Fe(2,6-$Mes_2C_6H_3$)(C(O)(2,6-$Mes_2C_6H_3$))(CO)$_3$

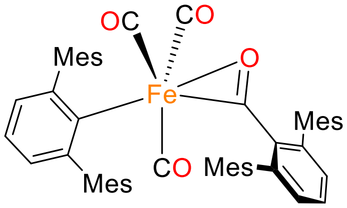

A solution of **1**^{Mes} (200 mg, 0.3 mmol) in *i*-hexane (20 mL) was placed under a partial vacuum to remove the argon from the headspace. The flask was backfilled with *ca.* 1 atm of CO, resulting in a colour change of the solution from yellow to orange. The reaction was left stirring until an orange precipitate formed, at which point the reaction was cooled in an ice bath (0 °C) and the stirring was stopped. The solution was filtered away from the precipitate through a Teflon cannula fitted with a glass fibre filter resulting in the isolation of **6** as a pale orange solid. Yield = 35 mg (0.04 mmol, 15 %).

Note: it has not been possible to characterise **6** cleanly by NMR spectroscopy as it readily reacts upon dissolution to form **4**^{Mes}, especially in the presence of an atmosphere of CO. In the absence of a CO atmosphere the reaction is slow and some peaks are observed but they are very broad and cannot be confidently assigned to one species (See **Figure S5**).

ATR-IR: $\nu_{(CO)}$ (cm^{-1}): 2077 (CO),2013 (CO), 1975 (CO), 1615 (Acyl).
ReactIR (C_6H_6, Silicon probe): 2076 cm^{-1}, 2015 cm^{-1}, 1980 cm^{-1}.
ReactIR (Toluene, Silicon probe): 2076 cm^{-1}, 2014 cm^{-1}, 1979 cm^{-1}.
HRMS (MALDI-TOF) *m/z*: [M–2(CO)]$^+$ calculated 738.31557, found 738.31732, err. = -2.36 ppm.

Isolation of complexes 5, 6, 7 and 14 for scXRD

A stirred solution of **1**^{Mes} (100 mg, 0.15 mmol) in C_6H_6 (30 mL) was exposed to an atmosphere of dry CO at room temperature whereupon an immediate colour change from yellow to orange occurred. The solution was stirred for 30 seconds (for crystals of **7** the solution was stirred for 2 minutes) until it turned from yellow to orange. The orange solution was then flash frozen in liquid nitrogen. The C_6H_6 was sublimed from the reaction mixture *in vacuo*, the resulting residue was then extracted into *i*-hexane (10 mL) and stored at 8 °C for 48 hours to obtain orange crystals of **5**, **6** and **7** and yellow crystals of **13** suitable for X-ray diffraction. Due to fast reactivity in solution, it has not been possible to isolate **5**, **7** and **13** independently for further analysis.

5

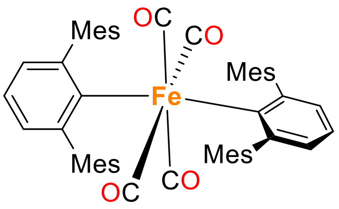

7

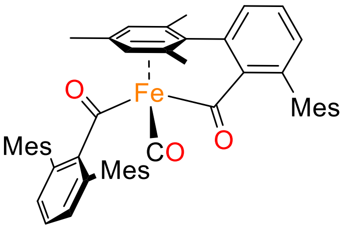

14

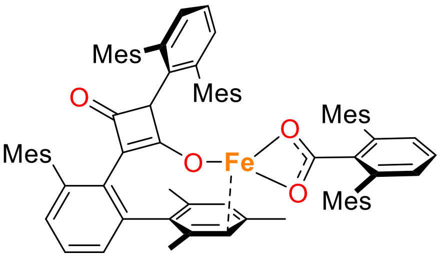

2.1.2 Characterisation of decomposition compounds

8, 2,6-$Mes_2C_6H_3$(H)C=C=O

Note: This has not been cleanly isolated but has been formed as the major product when decomposition has occurred. A loosely-sealed J Young NMR tube was left for 6 days resulting in a pale brown solution and no evident precipitation of **3**. The major product by NMR spectroscopy was found to be **8.** Attempts to isolate **8** led to decomposition and so assignment has been determined from crude samples.

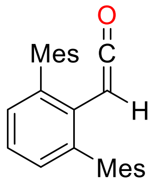

^{1}H NMR (500 MHz, C_6D_6): δ 7.04 (t, $^3J_{H\text{-}H}$ = 7.6 Hz, 1H, *p*-C_6H_3 Mes_2) 6.92 (d, $^3J_{H\text{-}H}$ = 7.6 Hz, 2H, *m*-C_6H_3 Mes_2), 6.88 (s, 4H, *m*-Mes), 3.60 (s, 1H, OCC*H*), 2.21 (s, 6H, *p*-C*H*$_3$), 2.07 (s, 12H, *o*-C*H*$_3$) ppm.
^{13}C{^{1}H} NMR (126 MHz, C_6D_6): δ 193.08 (*C*O), 138.25, 137.95, 137.64 (*i*-$C_6H_3Mes_2$), 136.83, 130.55 (*o*-C_6H_3 Mes_2), 128.84 (*m*-Mes), 125.03 (*p*-$C_6H_3Mes_2$), 24.85 (*C*CO), 21.24 (*p*-CH_3), 20.23 (*o*-CH_3). *m*-$C_6H_3Mes_2$ peak obscured by the signal for C_6D_6 as determined by HSQC.
ReactIR (C_6H_6, Silicon probe): 2097 cm^{-1}.
ReactIR (Toluene, Silicon probe): 2097 cm^{-1}.
Harrick cell (Toluene, CaF_2): 2099 cm^{-1}.
Harrick cell (Cyclohexane, CaF_2): 2100 cm^{-1}.
HRMS (EI) *m/z*: $[M]^+$ calculated 354.19782, found 354.19570, err. = -5.98 ppm.

8-^{13}C, 2,6-$Mes_2C_6H_3(H)^{13}C{=}^{13}C{=}O$
8-^{13}C forms while performing *in situ* reaction monitoring (^{1}H NMR and IR spectroscopy) due to degradation and has not been isolated cleanly. Spectroscopic data given below.

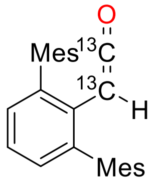

^{1}H NMR (500 MHz, C_6D_6): δ 7.04 (m, 1H, *p*-C_6H_3) 6.93 (m, 2H, *m*-C_6H_3), 6.88 (s, 4H, *m*-Mes), 3.60 (d, $^1J_{H\text{-}C}$ = 162 Hz, 1H, C*H*), 2.22 (s, 6H, *p*-C*H*$_3$), 2.07 (s, 12H, *o*-C*H*$_3$) ppm. It is not possible to accurately integrate and calculate coupling constants for the aryl groups due to multiple overlapping signals.
^{13}C{^{1}H} NMR (101 MHz, C_6D_6): δ 192.7 (d, $^1J_{C\text{-}C}$ = 108 Hz, C*C*O), 24.5 (d, $^1J_{C\text{-}C}$ = 108 Hz, *C*CO). Only the enriched carbons could be located.
Harrick cell (C_6H_6, CaF_2): 2038 cm^{-1}.

10

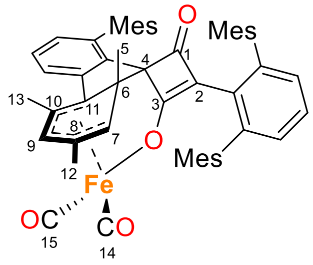

10 NMR tubes were filled with **4^{Mes}** (25 mg [0.03 mmol] × 10) and C_6H_6 (0.6 mL × 10) and thoroughly mixed. The tubes were left in a glovebox for 1 month after this the solutions were combined and filtered through a pipette fitted with a glass fibre filter, washing the precipitate with C_6H_6 until the washings were colourless. The solvent was removed *in vacuo* yielding a red solid. The solid was dissolved in Et_2O (2 mL) and *n*-hexane was added dropwise until a precipitate began to form. The solution was placed under a vacuum to remove some of the Et_2O, forcing more solid to precipitate. The dark precipitate was placed in the freezer for 5 days to ensure as much precipitate as possible had

formed. The solution was filtered resulting in a dark red precipitate with some crystals suitable for XRD. ^{1}H NMR spectroscopy indicated the sample consists of a 50:50 mixture of **9** and **2**^{Mes}.

Note on NMR assignment: not all peaks have been definitively assigned. Positions marked in the above figure have been assigned.

^{1}H NMR (500 MHz, C_6D_6): δ 7.16-7.12 (m), 6.99-6.97 (m), 6.91-6.83 (m), 4.09 (s, 1H, *H*-7), 3.54 (s, 1H, *H*-9), 2.65 (s, 6H, Ar-C*H*$_3$), 2.30 (s, 3H, Ar-C*H*$_3$), 2.25 (s, 3H, Ar-C*H*$_3$), 2.18 (s, 6H, Ar-C*H*$_3$), 2.01 (s, 6H, Ar-C*H*$_3$), , 1.62 (s, 3H, *H-12*), 1.60 (s, 3H, *H-13*), 1.60 (s, 3H, Ar-C*H*$_3$), 0.70 (s, 3H, *H-5*). There is substantial overlap of the aryl signals, making accurate integration difficult.
^{13}C{^{1}H} NMR (101 MHz, C_6D_6): δ 207.4 (*C*-15), 204.6 (*C*-14), 191.1 (*C*-1), 181.7 (*C*-3), 146.1, 142.6, 141.8, 139.8, 137.27, 136.9, 136.1, 135.4, 134.0, 133.7, 134.0, 131.2, 128.6, 128.4 (located by HSQC), 128.3 (located by HSQC), 128.0 (located by HSQC), 127.7, 123.26, 123.22 (*C*-2), 114.4 (*C*-8), 103.5 (*C*-10), 103.4 (*C*-11), 89.3 (*C*-4), 82.9 (*C*-7), 69.9 (*C*-9), 55.6 (*C*-6), 28.4 (*C*-5), 22.6-22.4, 22.1, 21.4, 21.3, 21.0 (*C*-12), 19.9 (*C*-13).
ATR-IR: $\nu_{(CO)}$ (cm^{-1}): 2027 cm^{-1}, 1980 cm^{-1}, 1732 cm^{-1}

10-^{13}C

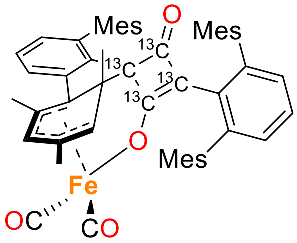

Only signals showing coupling due to ^{13}C enrichment given below.
^{1}H NMR (500 MHz, C_6D_6): δ 0.70 (d, $^3J_{H\text{-}C}$ = 3 Hz, 3H, *H*-5).
^{13}C{^{1}H} NMR (101 MHz, C_6D_6): δ 207.4 (d, $^2J_{C15\text{-}C14}$ = 14 Hz, *C*-15), 204.6 (d, $^2J_{C14\text{-}C15}$ = 14 Hz, *C*-14), 191.1 (ddd, $^1J_{C1\text{-}C2}$ = 66 Hz, $^1J_{C1\text{-}C4}$ = 33 Hz, $^2J_{C1\text{-}C3}$ = 4 Hz, *C*-1), 181.7 (ddd, $^1J_{C3\text{-}C2}$ = 54 Hz, $^1J_{C3\text{-}C4}$ = 37 Hz, $^2J_{C3\text{-}C1}$ = 4 Hz, *C*-3), 146.1 (d, $J_{C\text{-}C}$ = 45 Hz, C_{Ar}), 141.8 (d, $J_{C\text{-}C}$ = 2 Hz, C_{Ar}), 139.8 (t, $J_{C\text{-}C}$ = 3 Hz, C_{Ar}), 134.0 (d, $J_{C\text{-}C}$ = 3 Hz, C_{Ar}), 131.2 (dd, $J_{C\text{-}C}$ = 59 Hz, $J_{C\text{-}C}$ = 9 Hz, C_{Ar}), 127.7 (d, $J_{C\text{-}C}$ = 8 Hz, C_{Ar}), 123.22 (ddd, $^1J_{C2\text{-}C1}$ = 66 Hz, $^1J_{C2\text{-}C3}$ = 54 Hz, $^2J_{C2\text{-}C4}$ = 31 Hz, *C*-2), 114.4 ($^3J_{C\text{-}C}$ = 3 Hz, *C*-8), 103.4 (m, *C*-11), 89.3 (ddd, $^1J_{C4\text{-}C3}$ = 37 Hz, $^1J_{C4\text{-}C1}$ = 33 Hz, $^2J_{C4\text{-}C2}$ = 31 Hz, *C*-4), 55.6 (d, $^1J_{C\text{-}C}$ = 34 Hz, *C*-6).

2.2 NMR spectra

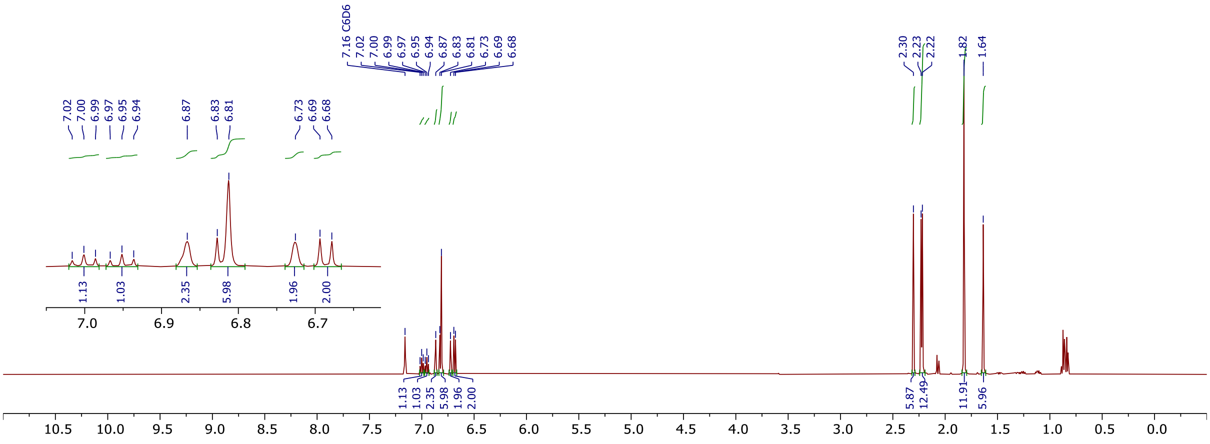

Figure S1 - ^{1}H NMR spectrum of **4^{Mes}** in C_6D_6 at 25 °C. Inset is a close-up of the aryl region.

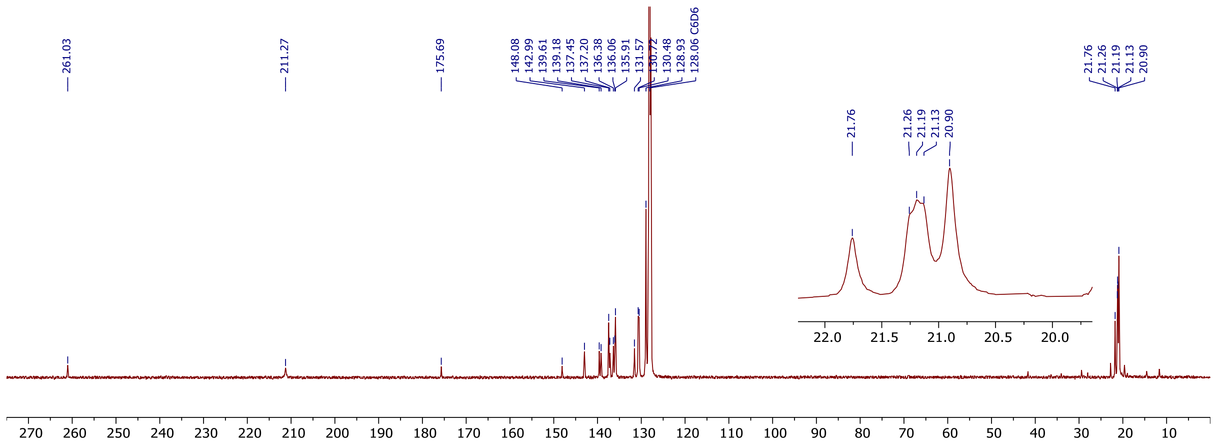

Figure S2 – $^{13}C\{^1H\}$ NMR spectrum of $\mathbf{4^{Mes}}$ in C_6D_6 at 25 °C. Inset is a close-up of the methyl peaks.

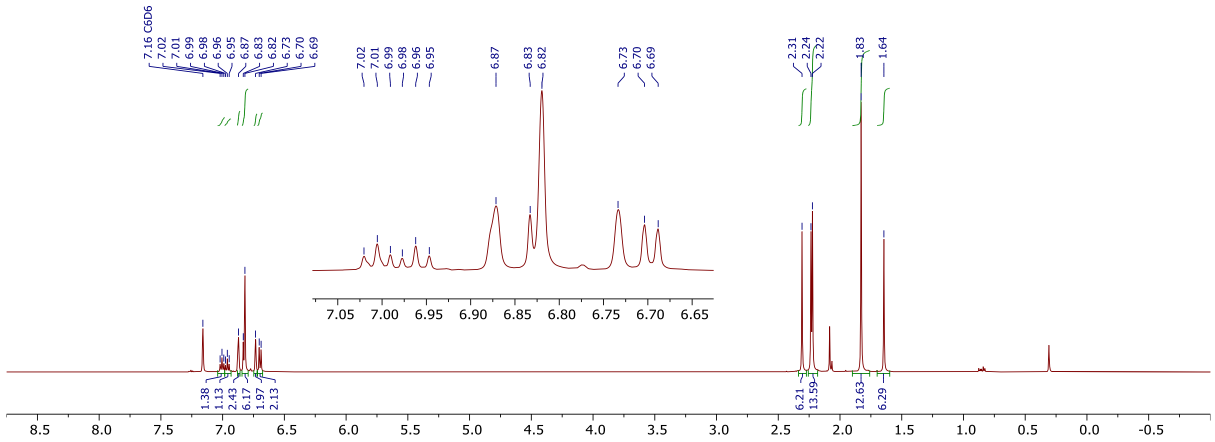

Figure S3 - ^1H NMR spectrum of $\mathbf{4^{Mes\text{-}13C}}$ in C_6D_6 at 25 °C. Inset is a close-up of the aryl region.

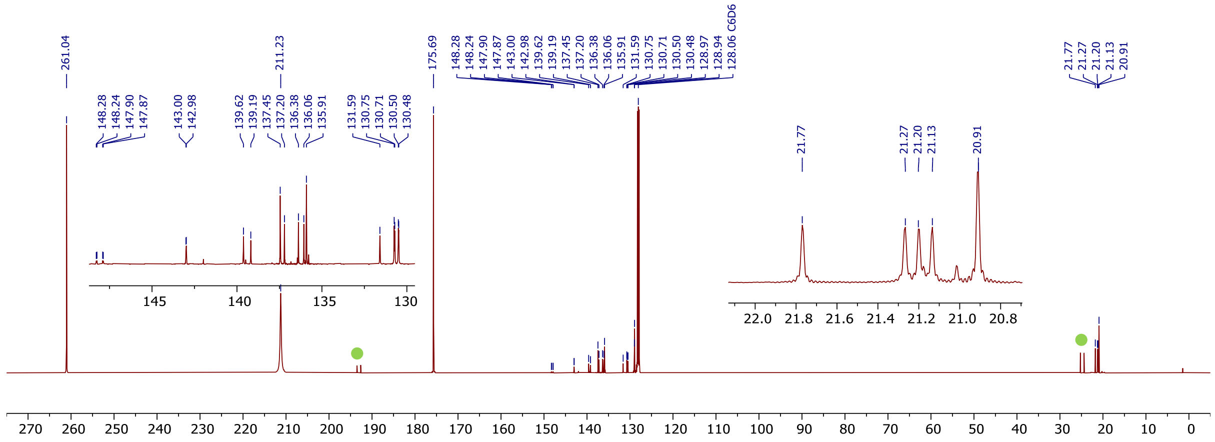

Figure S4 – $^{13}C\{^1H\}$ NMR spectrum of $\mathbf{4^{Mes\text{-}13C}}$ in C_6D_6 at 25 °C. Inset are a close-up of the aryl peaks showing ^{13}C-^{13}C coupling and the methyl peaks. $\mathbf{8\text{-}^{13}C}$ highlighted in green, formed by partial decomposition during collection.

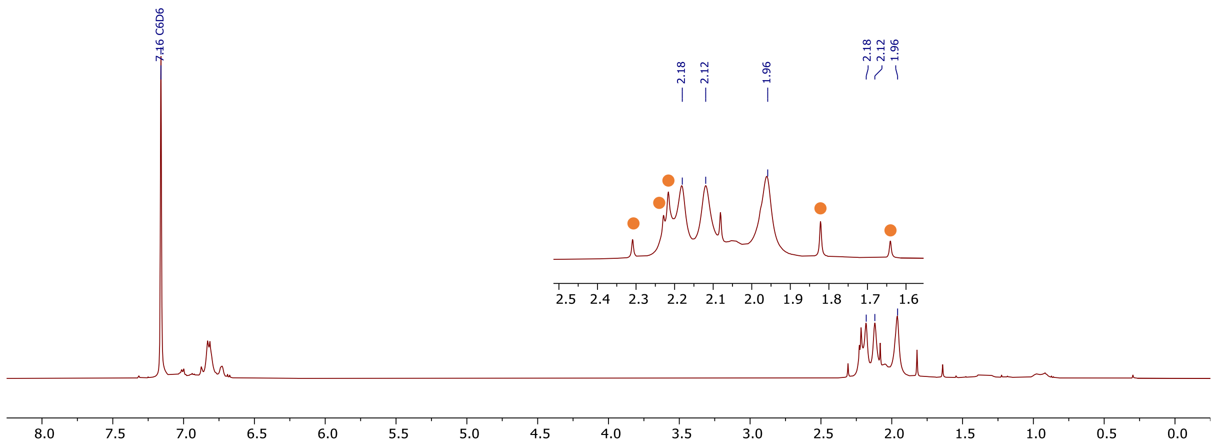

Figure S5 – ^{1}H NMR spectrum of **6** in C_6D_6 at 25 °C. Signals for $\mathbf{4}^{\mathbf{Mes}}$ (highlighted with orange) are seen within 10 minutes of dissolution of **6**. Inset is a close-up of alkyl region.

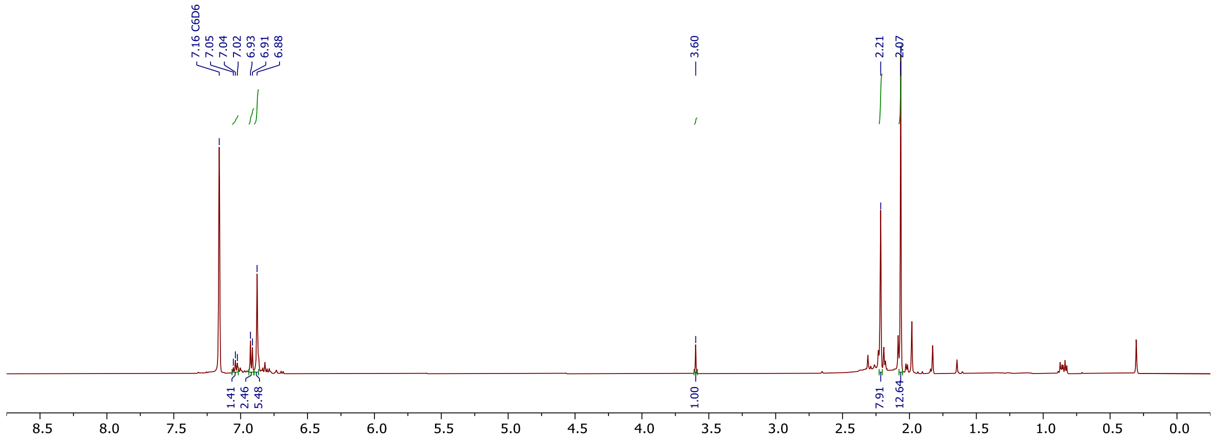

Figure S6 - ^{1}H NMR spectrum of a crude mix of **8** in C_6D_6 at 25 °C.

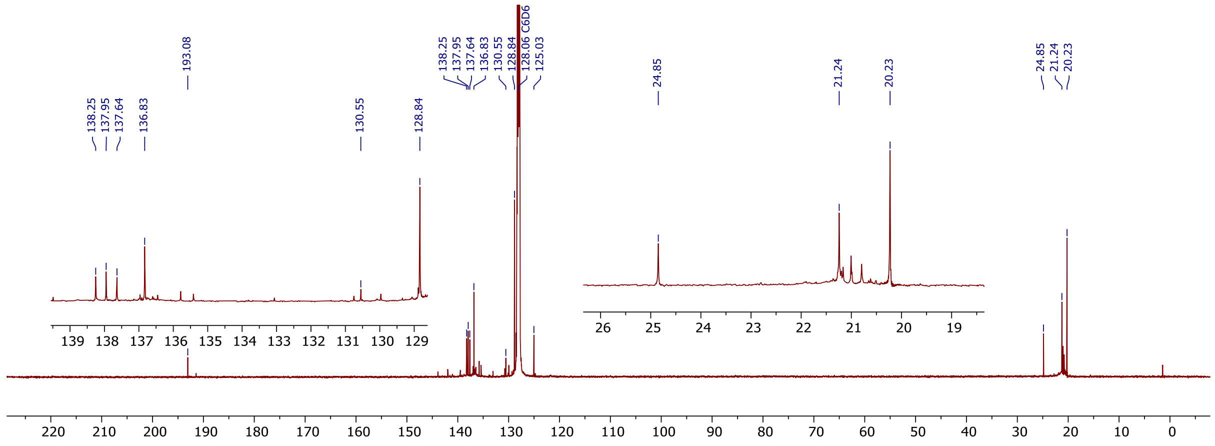

Figure S7 – $^{13}C\{^{1}H\}$ NMR spectrum of a crude mix of **8** in C_6D_6 at 25 °C.

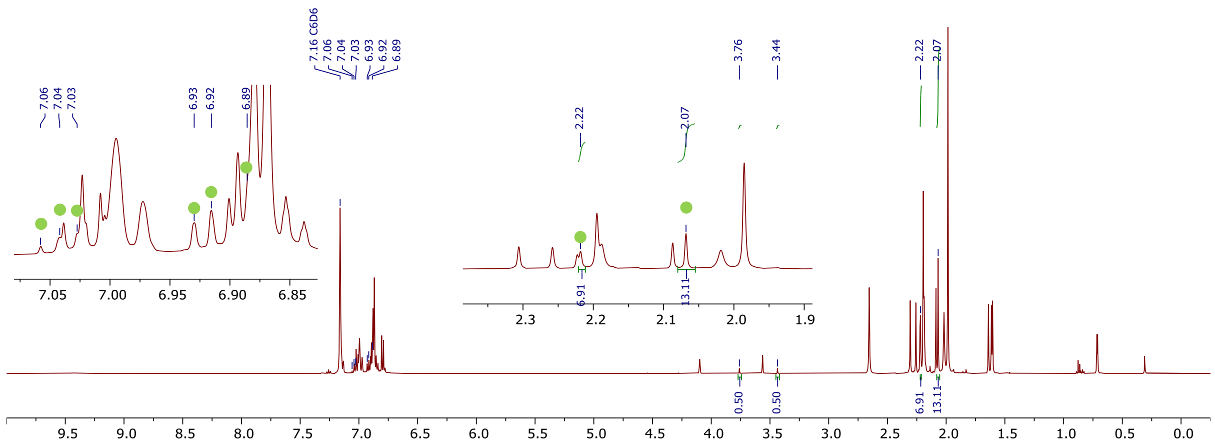

Figure S8 - ^{1}H NMR spectrum of **8-^{13}C** in C_6D_6 at 25 °C. **8-^{13}C** marked in green. Spectrum collected after filtration to remove paramagnetic species. **10-^{13}C** and squaraine (**2^{Mes}**) also present.

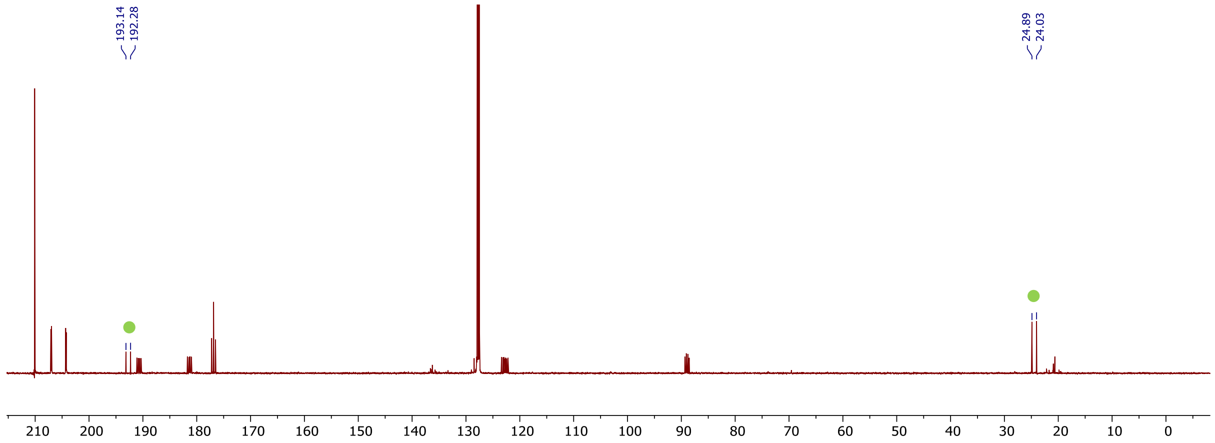

Figure S9 – ^{13}C{^{1}H} NMR spectrum of **8-^{13}C** in C_6D_6 at 25 °C. **8-^{13}C** marked in green. Spectrum collected after filtration to remove paramagnetic species. **10-^{13}C** and squaraine (**2^{Mes}**) also present.

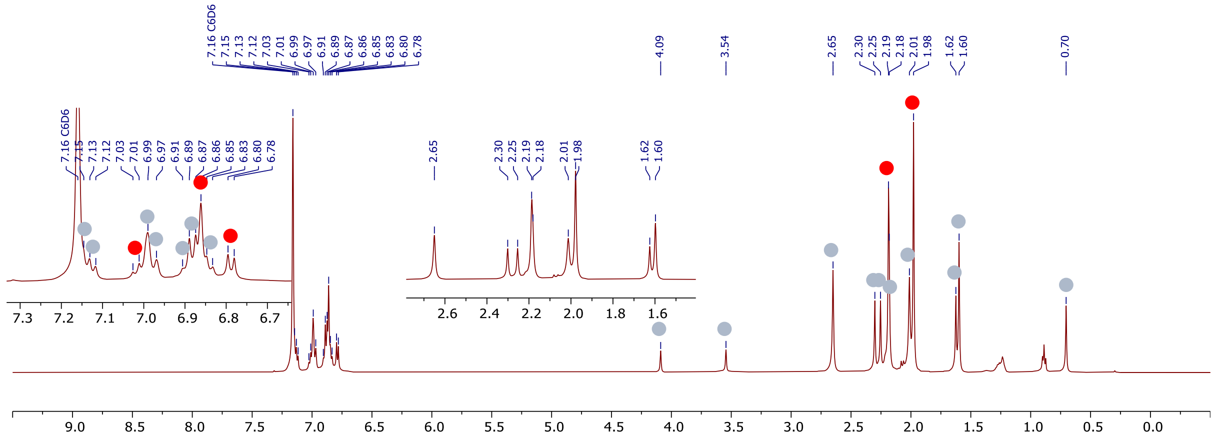

Figure S10 - ^{1}H NMR spectrum of a mixture of **10** and **2^{Mes}** in C_6D_6 at 25 °C. **2^{Mes}** marked with red circle, **10** marked with blue circles.

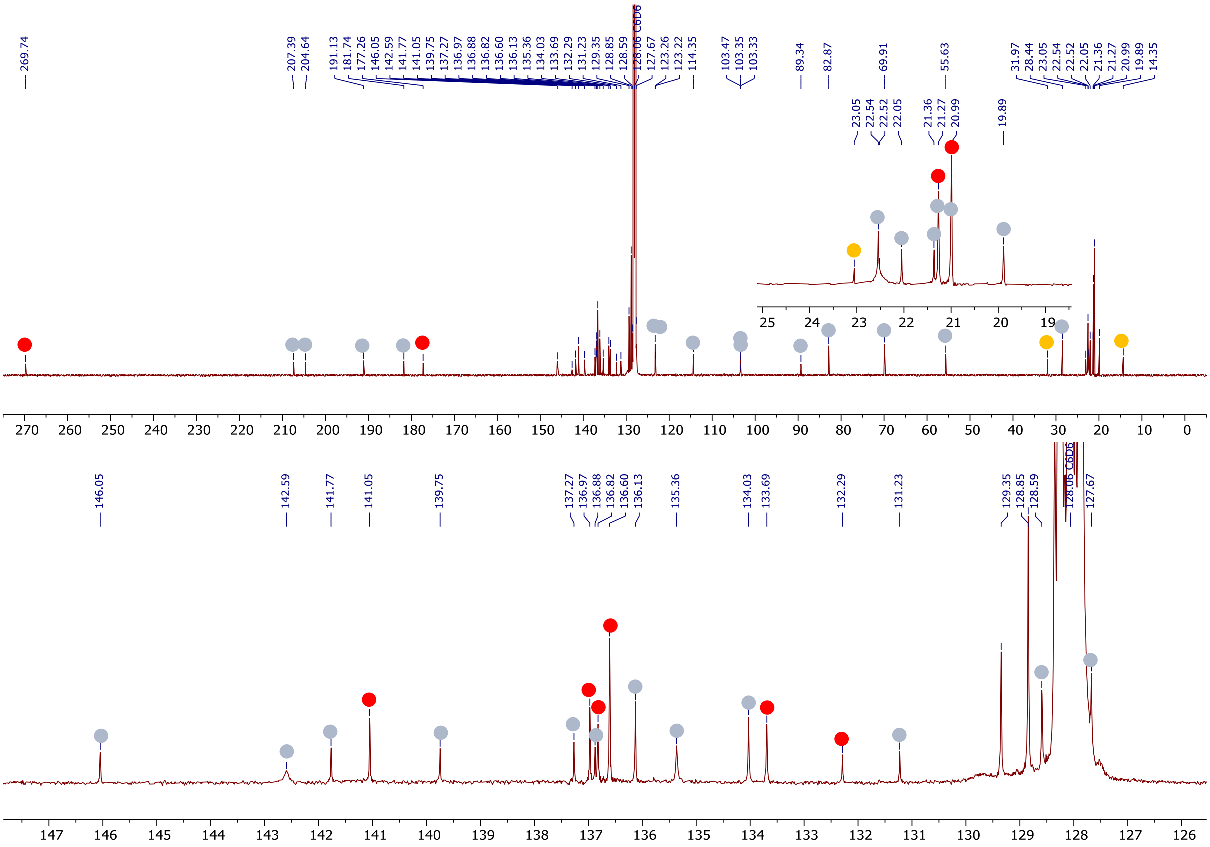

Figure S11- $^{13}C\{^{1}H\}$ NMR spectrum of a mixture of **10** and **2^{Mes}** in C_6D_6 at 25 °C. Top, full spectrum. Bottom, zoom in of aryl region. **2^{Mes}** marked with red circle, **10** marked with blue circles, yellow is residual *n*-hexane.

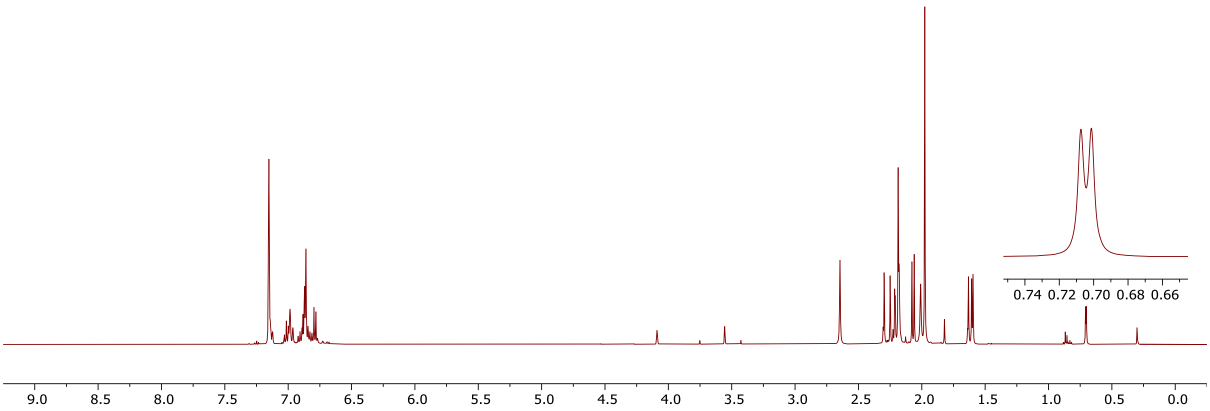

Figure S12 - ^{1}H NMR spectrum of a reaction of **1^{Mes}** with ^{13}CO in C_6D_6 at 25 °C. Reaction mixture contains **10-^{13}C** alongside **2^{Mes}-^{13}C, 8-^{13}C** and 2,6-Mes-C_6H_4. Inset is a zoom in on the peak of **10-^{13}C** showing ^{1}H-^{13}C coupling.

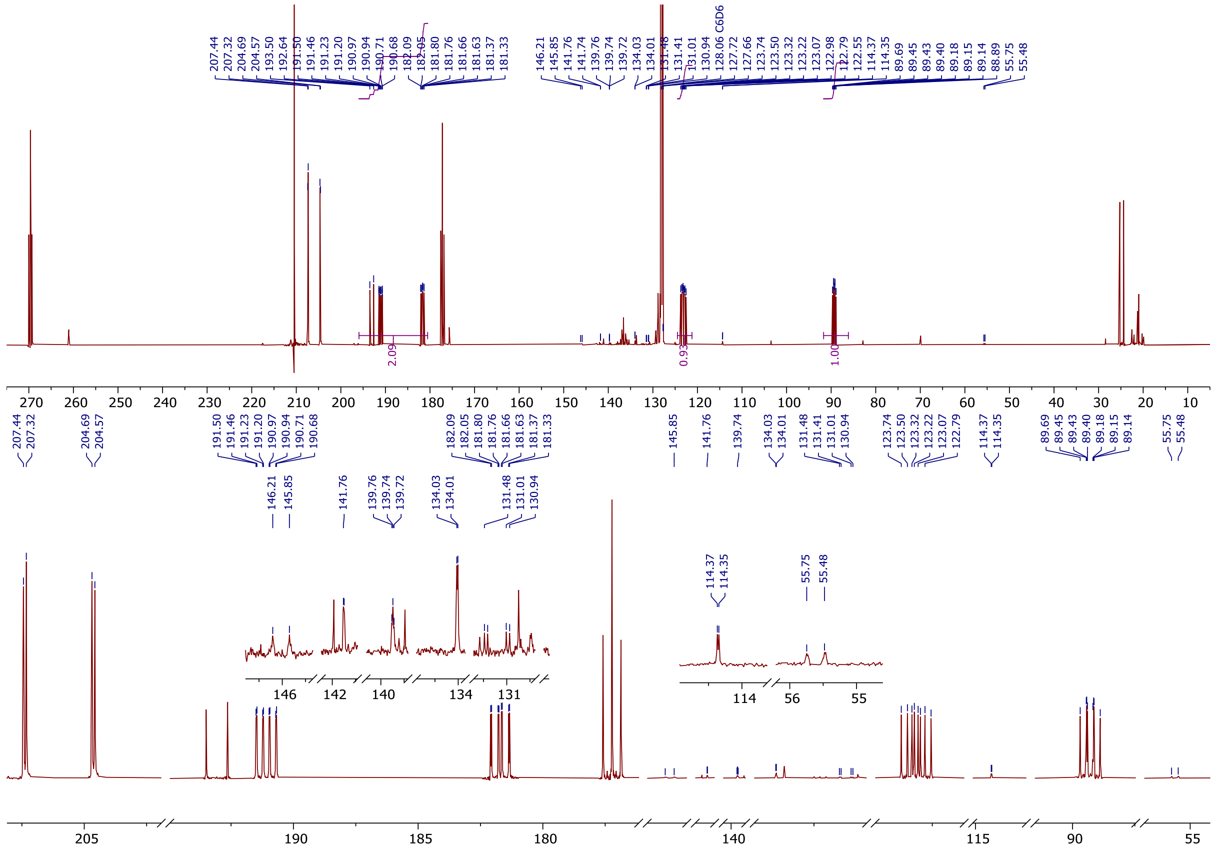

Figure S13 – $^{13}C\{^{1}H\}$ NMR spectrum of a reaction of **1^{Mes}** with ^{13}CO in C_6D_6 at 25 °C. Reaction mixture contains **10-^{13}C** alongside **2^{Mes}-^{13}C, 8-^{13}C** and 2,6-Mes-C_6H_4. Top, full spectrum. Bottom and inset, zoom in on peaks of **10-^{13}C** showing ^{13}C-^{13}C coupling.

2.3 IR spectra

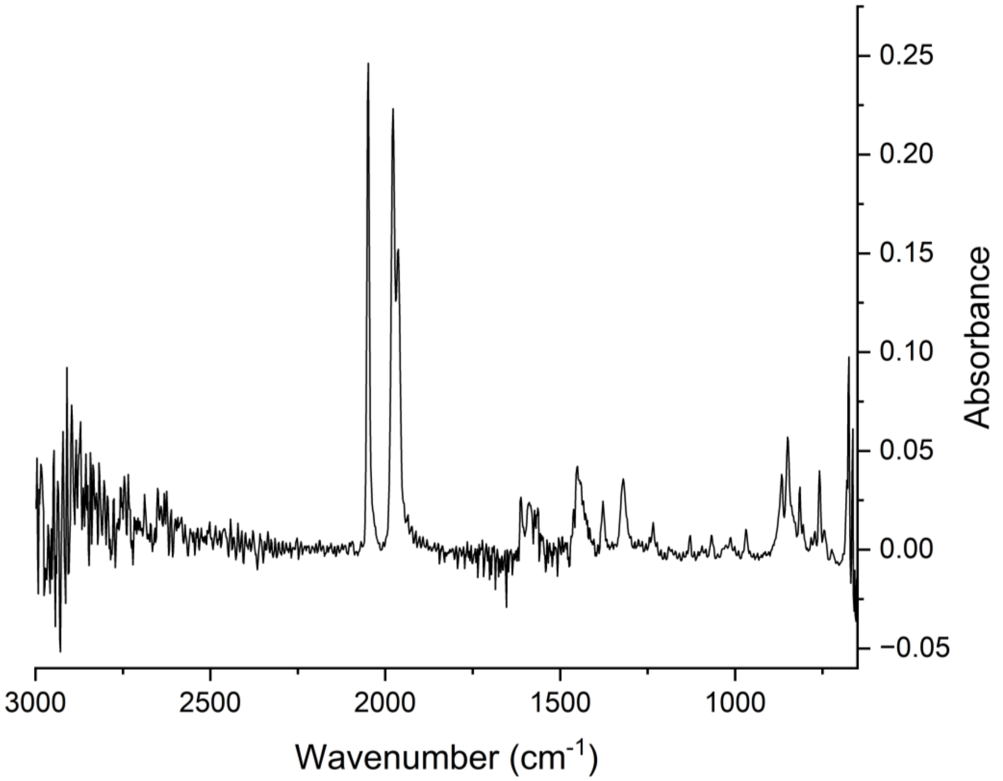

Figure S14 – Solution IR spectrum of **4^Mes^** recorded in C_6H_6 using a ReactIR spectrometer with a silicon probe. Full spectra shown. Solvent subtraction performed using iC IR to give the spectrum of **4^Mes^**.

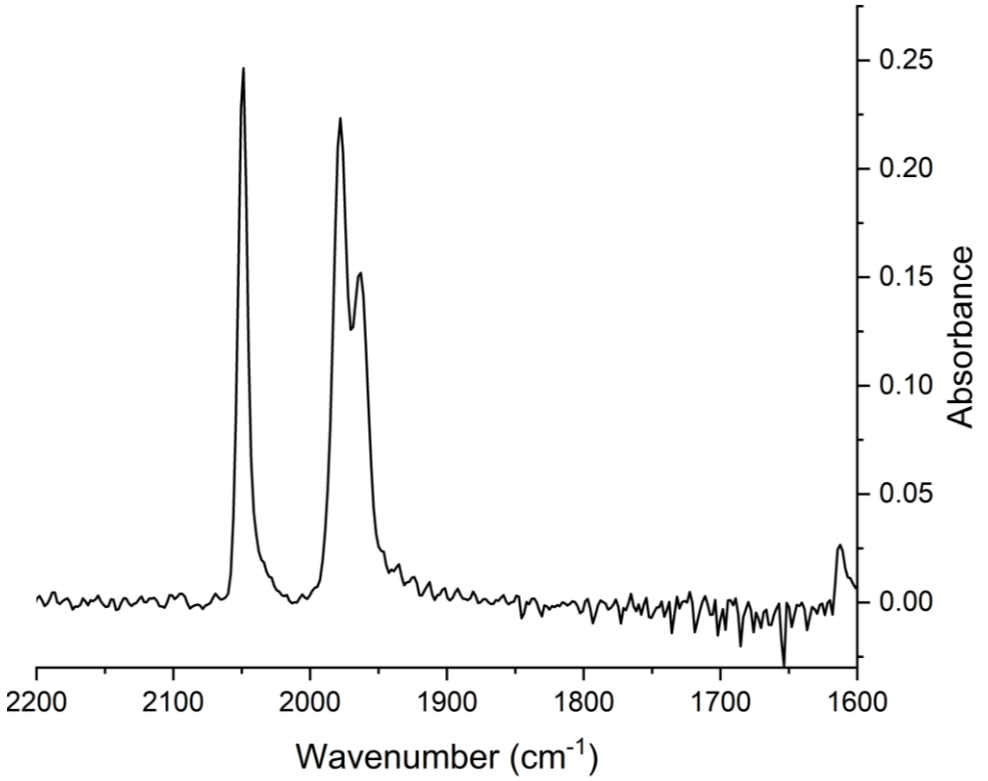

Figure S15 – Solution IR spectrum of **4^Mes^** recorded in C_6H_6 using a ReactIR spectrometer with a silicon probe. 2200 – 1600 cm^{-1} shown to clearly see the CO stretches. Solvent subtraction performed using iC IR to give the spectrum of **4^Mes^**.

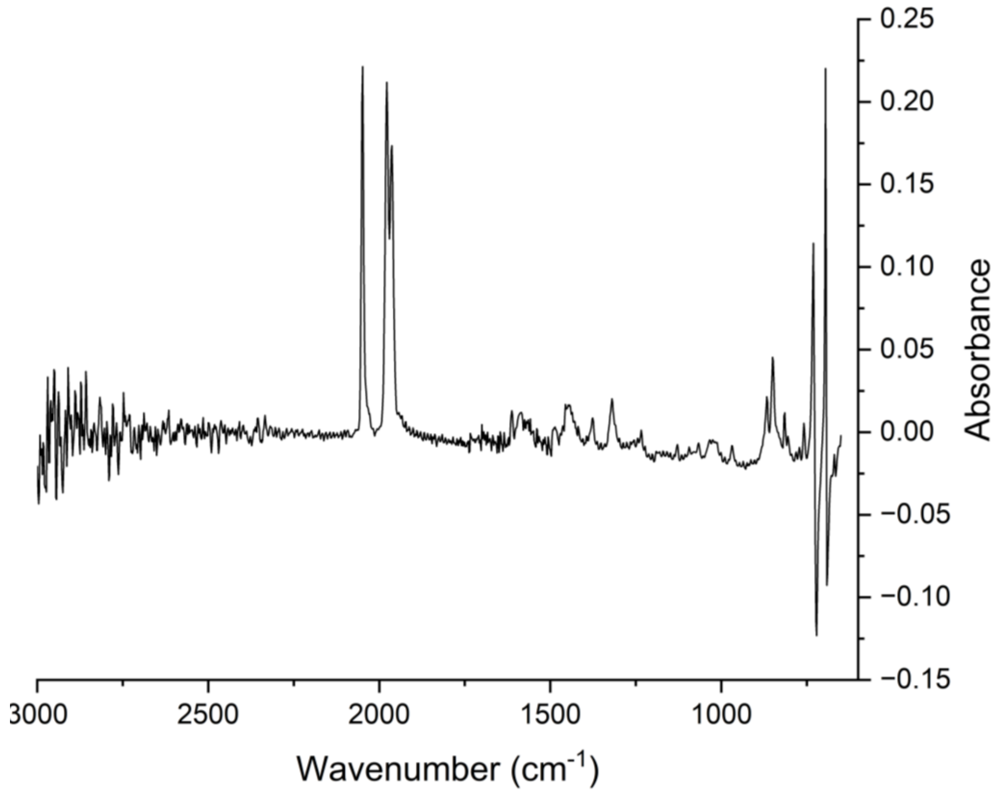

Figure S16 – Solution IR spectrum of **4^{Mes}** recorded in toluene using a ReactIR spectrometer with a silicon probe. Full spectra shown. Solvent subtraction performed using iC IR to give the spectrum of **4^{Mes}**.

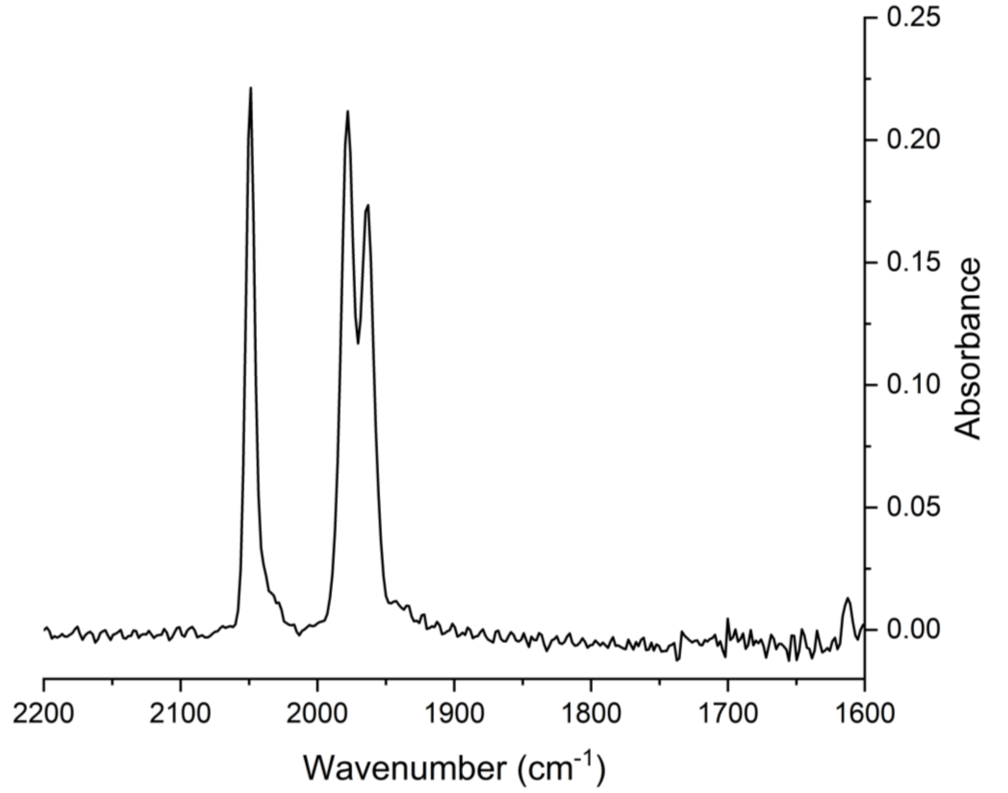

Figure S17 – Solution IR spectrum of **4^{Mes}** recorded in toluene using a ReactIR spectrometer with a silicon probe. 2200-1600 cm^{-1} shown to clearly see the CO stretches. Solvent subtraction performed using iC IR to give the spectrum of **4^{Mes}**.

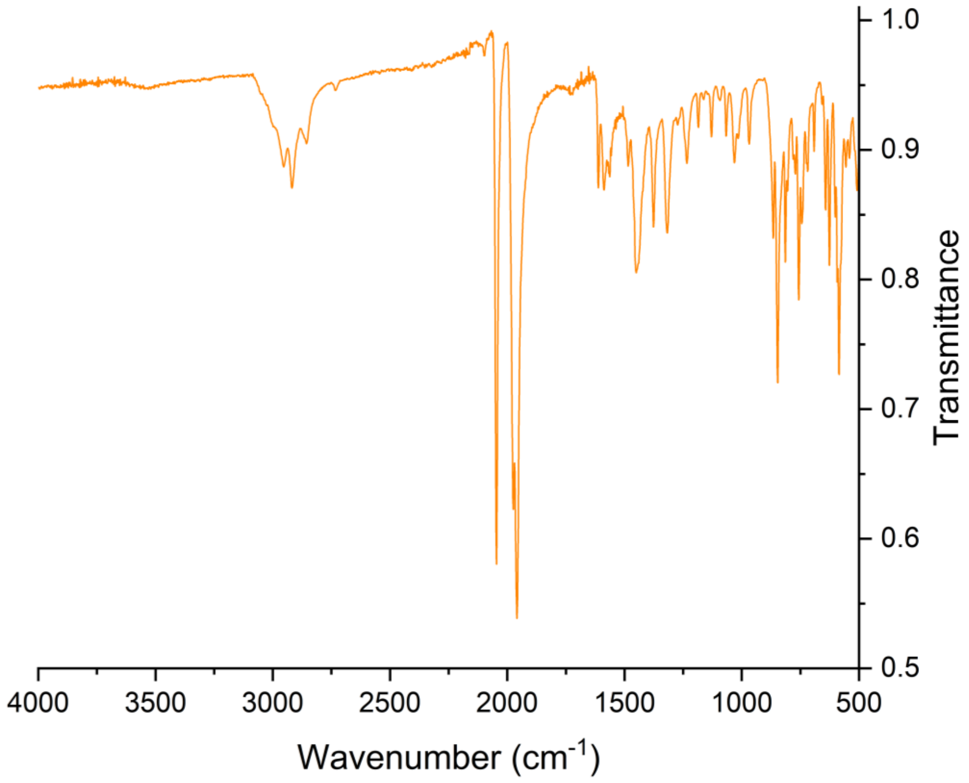

Figure S18 – ATR-IR spectrum of **4**^{Mes}. Full spectrum shown.

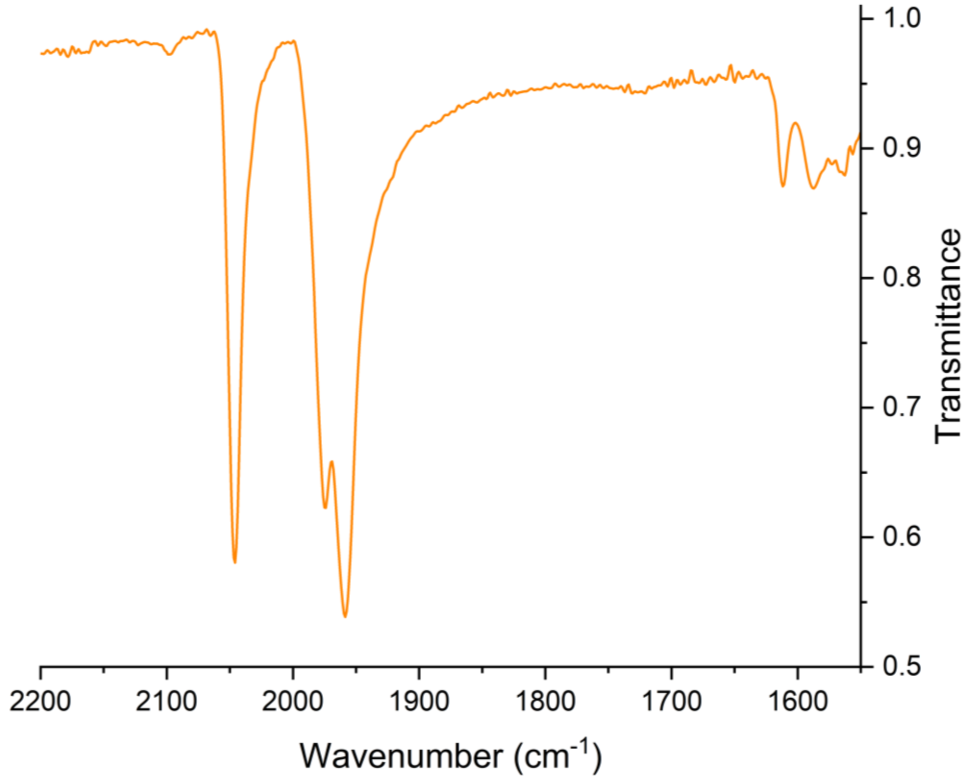

Figure S19 – ATR-IR spectrum of **4**^{Mes}. 2200-1550 cm^{-1} shown to clearly see the CO stretches.

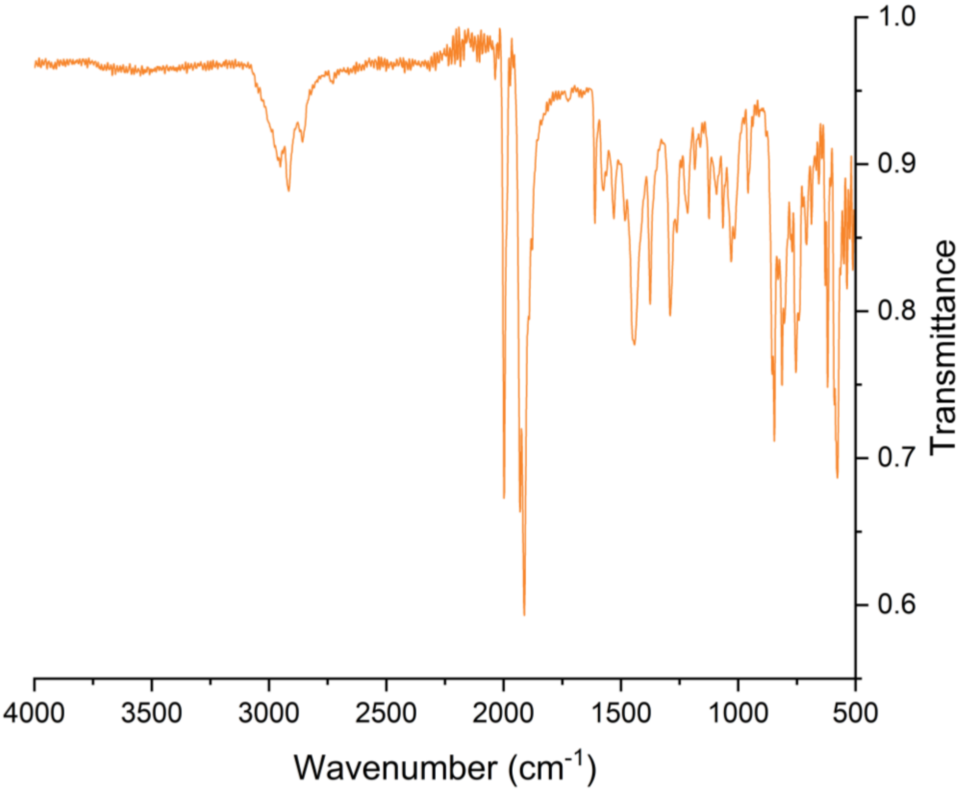

Figure S20 - ATR-IR spectrum of **4^{Mes}-^{13}C**. Full spectrum shown.

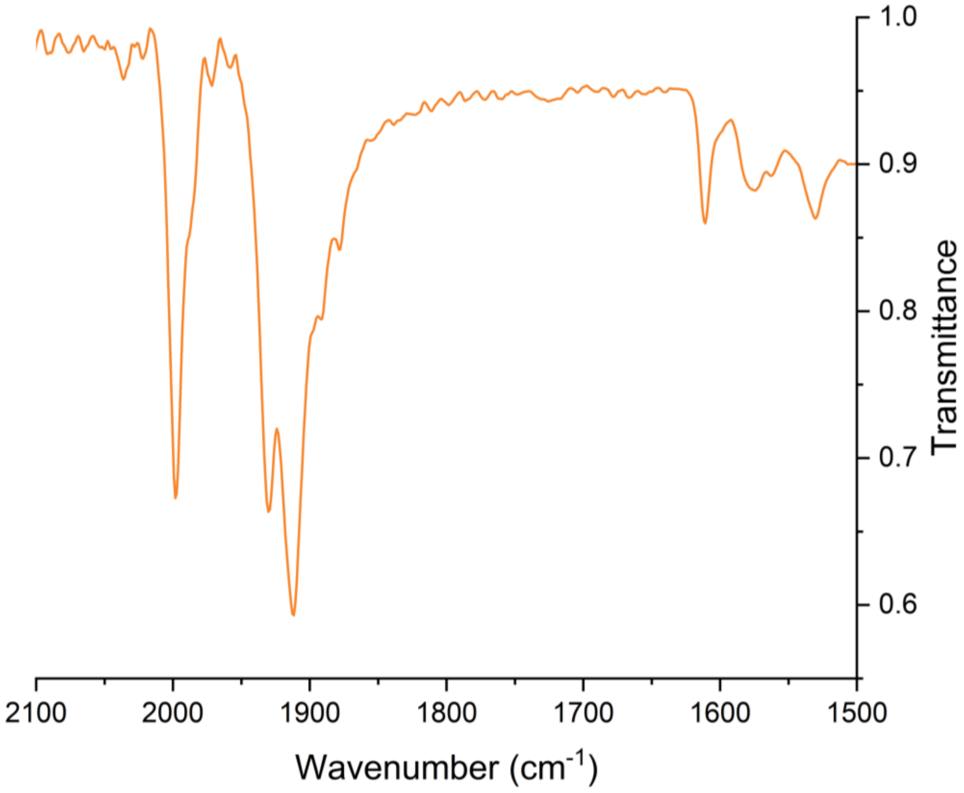

Figure S21 - ATR-IR spectrum of **4^{Mes}-^{13}C**. 2100-1550 cm^{-1} shown to clearly see the CO stretches.

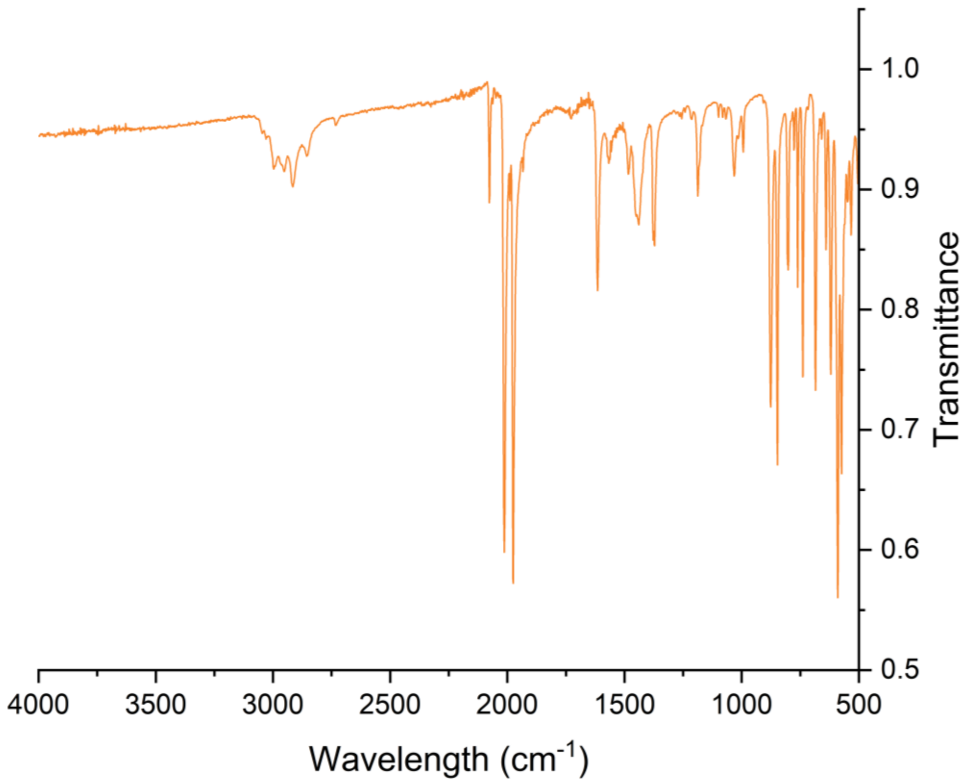

Figure S22 – ATR-IR spectrum of **6**. Full spectrum shown.

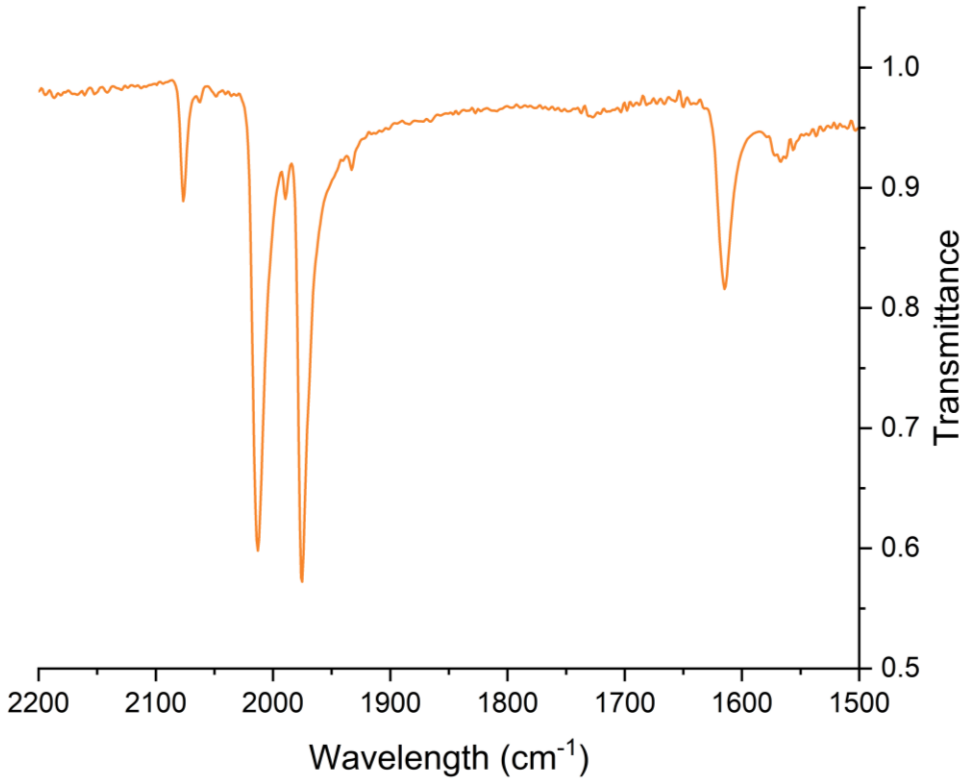

Figure S23 – ATR-IR spectrum of **6**. 2200-1500 cm^{-1} shown to clearly see the CO stretches**.**

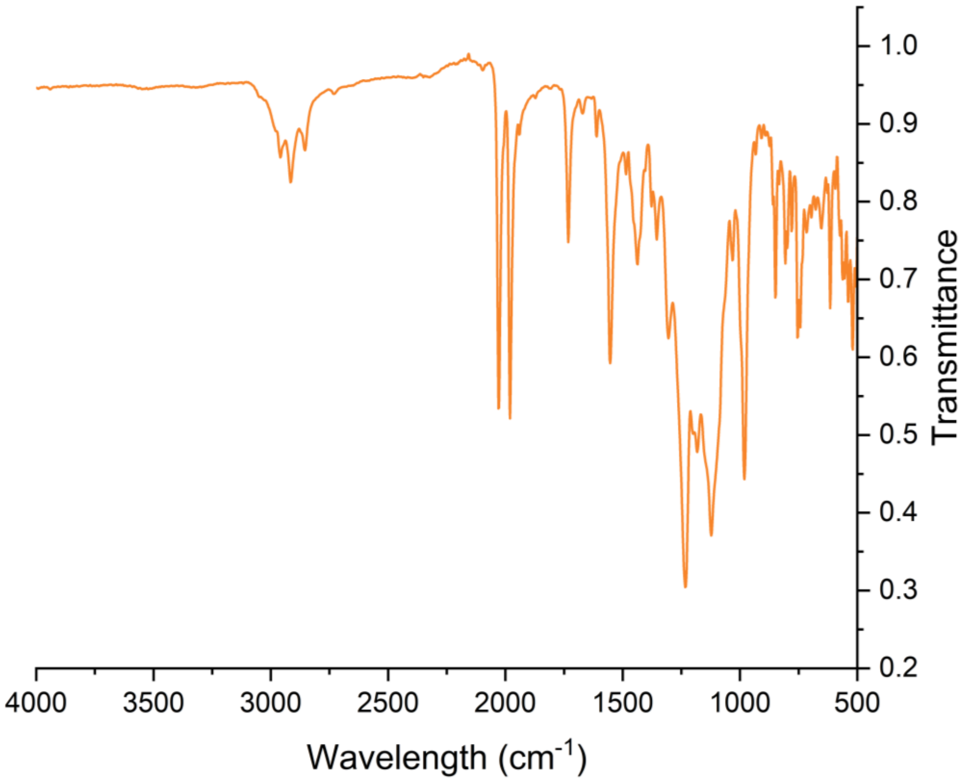

Figure S24 – ATR-IR spectrum of **10**, under Fomblin®. Full spectrum shown.

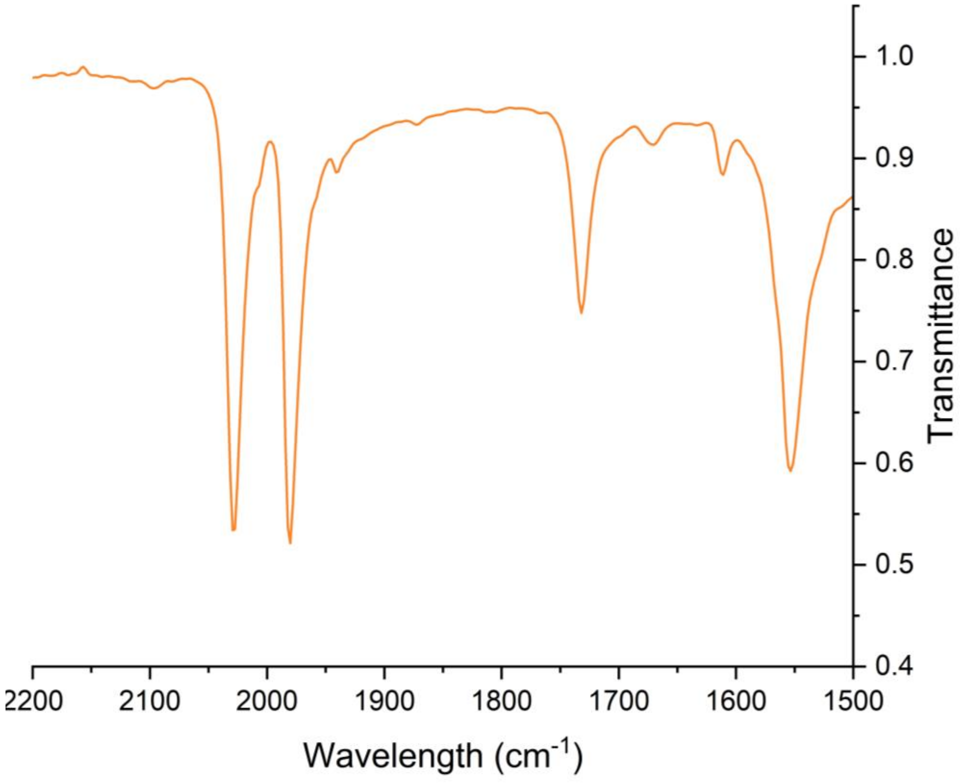

Figure S25 – ATR-IR spectrum of **10**, under Fomblin®. 2200-1500 cm^{-1} shown to clearly see the CO stretches**.**

2.4 Mass spectra

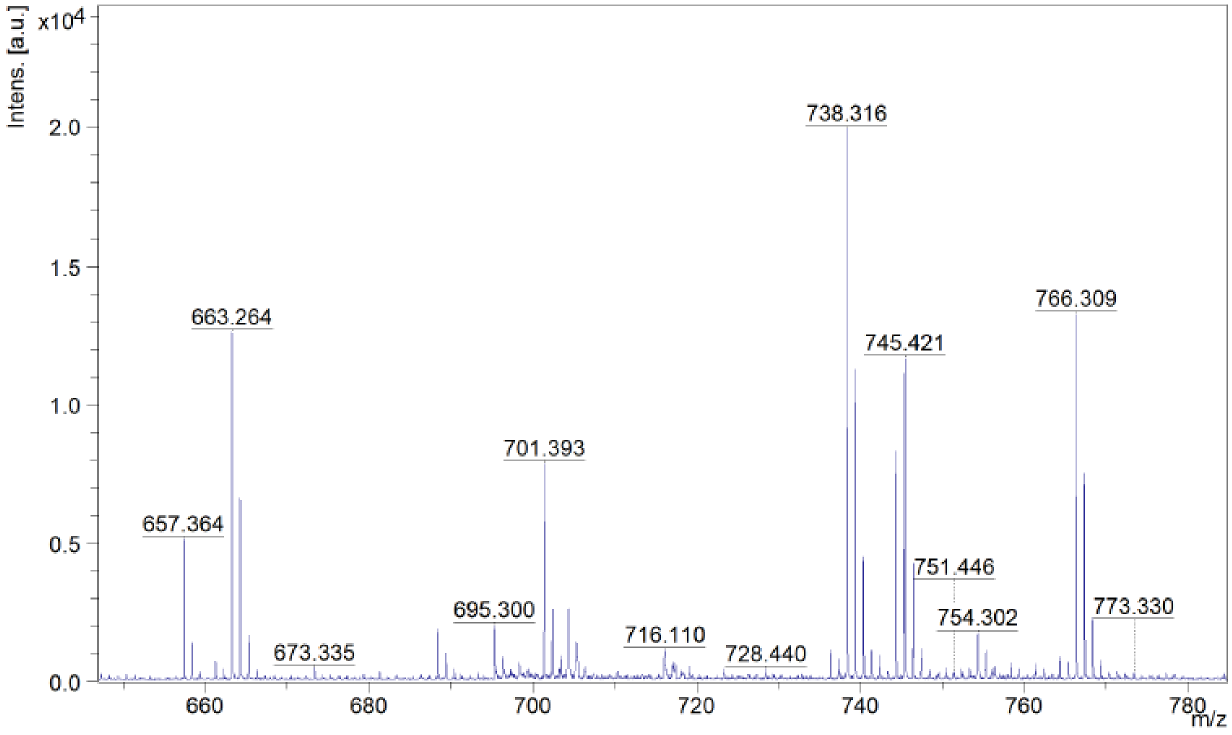

Figure S26 – Mass spectrum for **4^{Mes}** between 650 and 780 m/z.

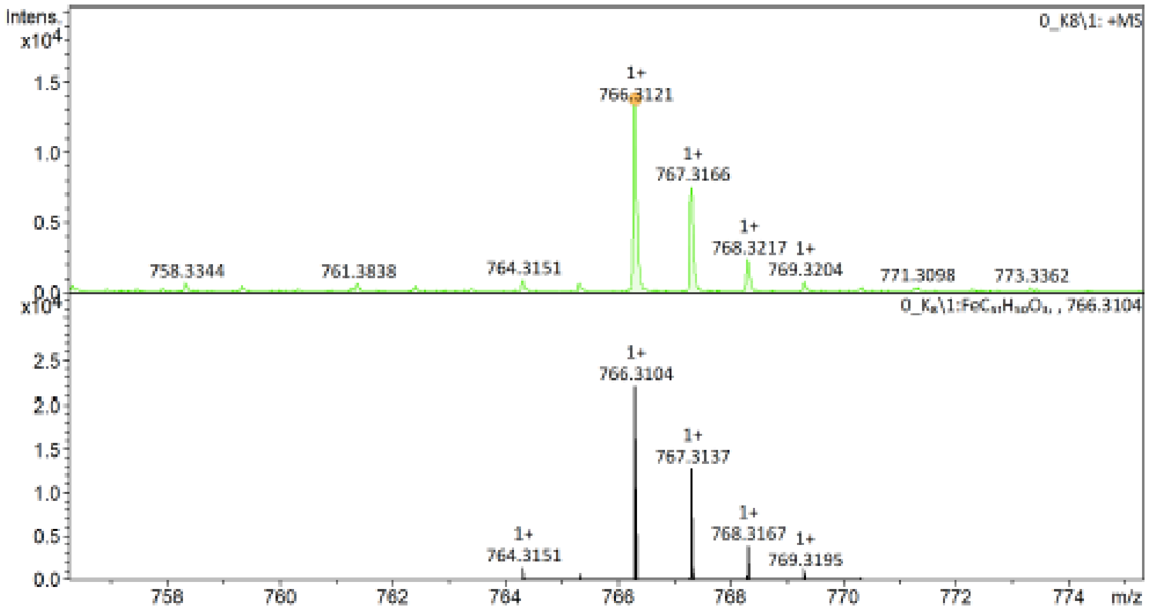

Meas. m/z	#	Ion Formula	Score	m/z	err [mDa]	err [ppm]	mSigma
766.3121	1	C51H50FeO3	100.00	766.3104	-1.6	-2.1	12.6

Figure S27 – Zoom in on the m/z peak at 766.3121 in the mass spectrum of **4^{Mes}**, [M–2(CO)]$^{+}$. Top, collected data. Bottom, calculated spectrum.

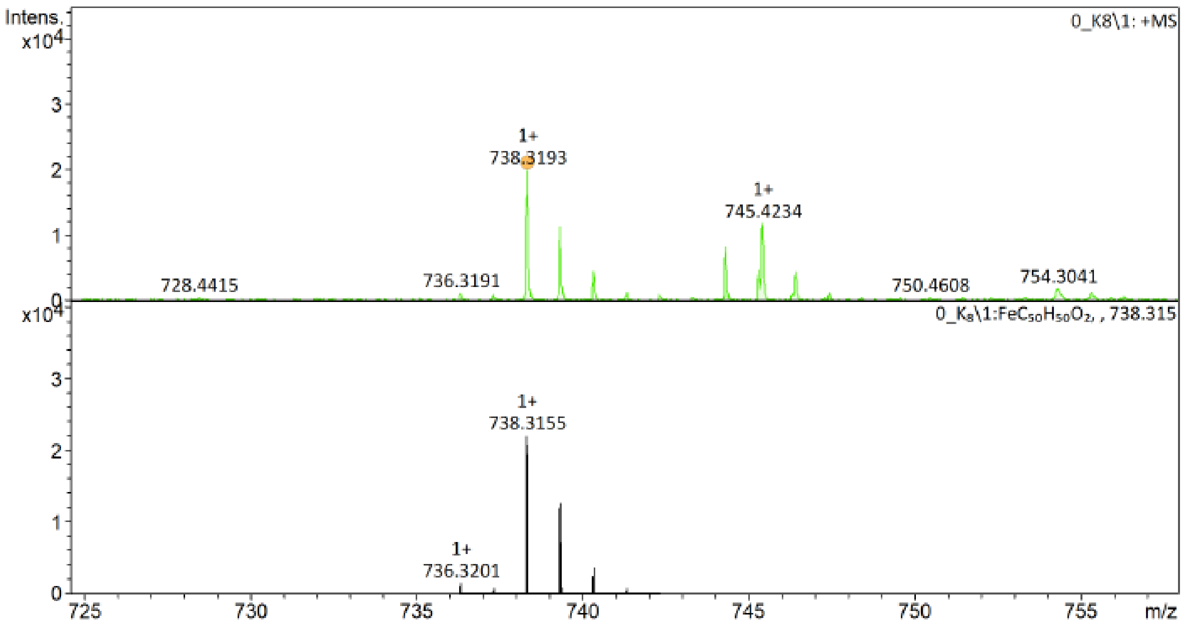

Meas. m/z	#	Ion Formula	Score	m/z	err [mDa]	err [ppm]	mSigma
738.3193	1	C50H50FeO2	100.00	738.3155	-3.7	-5.0	26.8

Figure S28 - Zoom in on the m/z peak at 738.3193 in the mass spectrum of **4^{Mes}**, [M–3(CO)]$^+$. Top, collected data. Bottom, calculated spectrum.

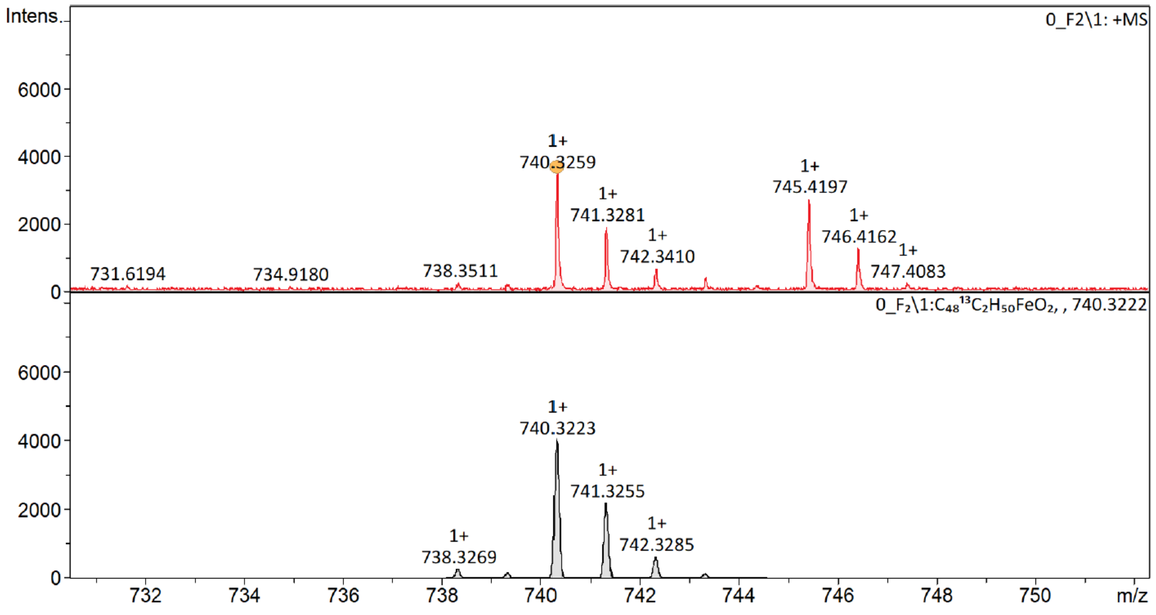

Meas. m/z	#	Ion Formula	Score	m/z	err [mDa]	err [ppm]	mSigma
740.3259	1	C48H50FeO2^13C2	100.00	740.3222	-3.6	-4.9	44.2

Figure S29 - Zoom in on the m/z peak at 740.3259 in the mass spectrum of **4^{Mes}-^{13}C,** [M–3(CO)]$^+$. Top, collected data. Bottom, calculated spectrum.

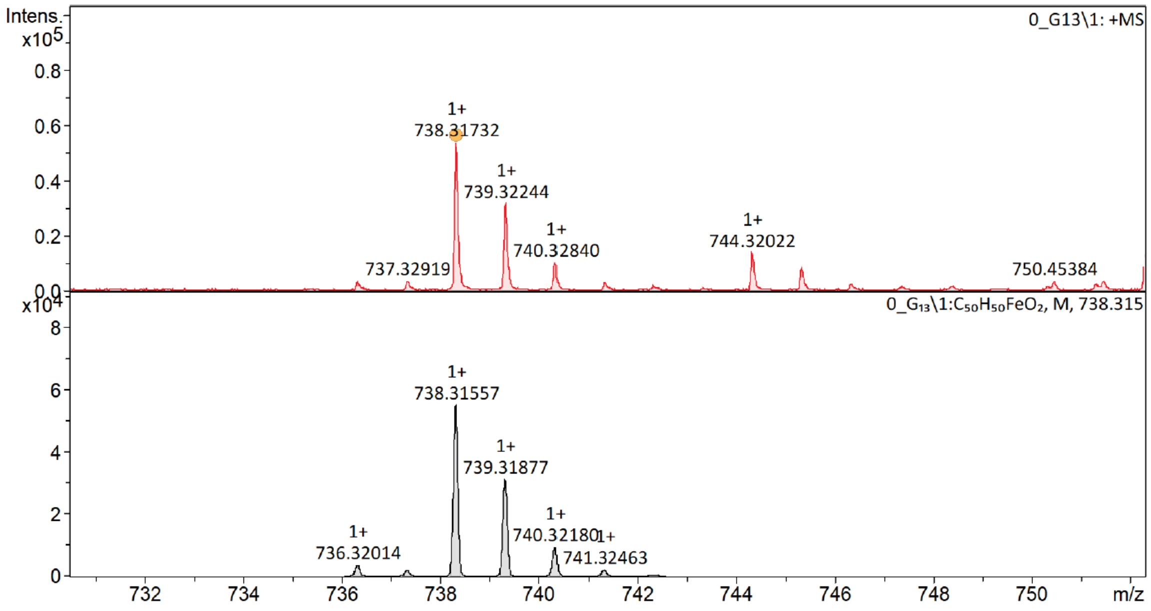

Meas. m/z	#	Ion Formula	Score	m/z	err [mDa]	err [ppm]	mSigma
738.31732	1	C50H50FeO2	100.00	738.31547	-1.75	-2.36	21.1

Figure S30 - Zoom in on the m/z peak at 738.31732 in the mass spectrum of **6**, $[M\text{-}2(CO)]^{+}$. Top, collected data. Bottom, calculated spectrum.

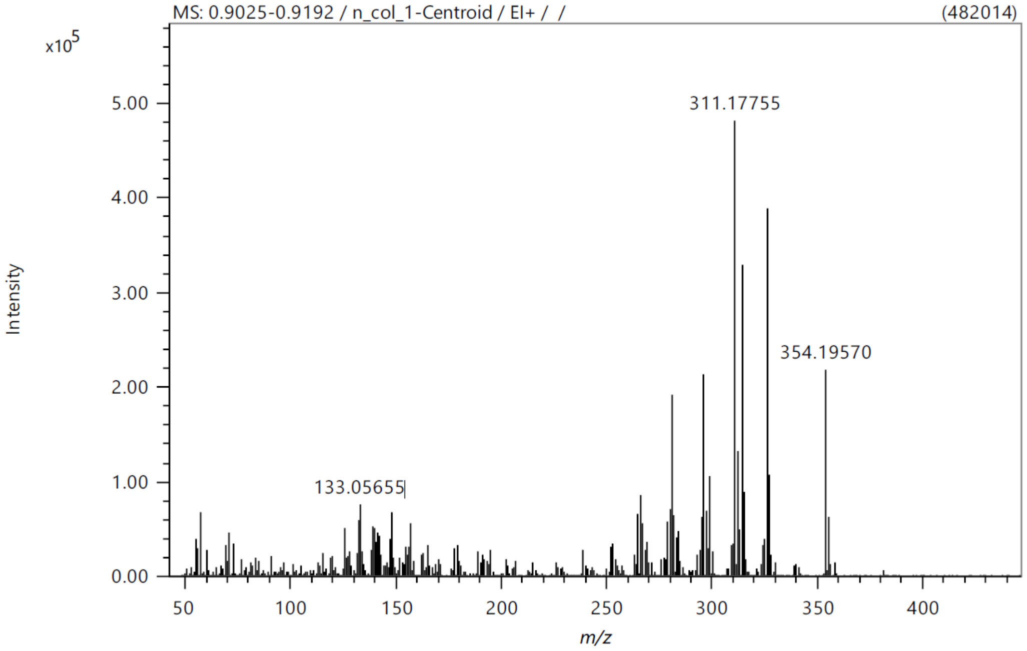

Figure S31 – Full mass spectrum for **8**.

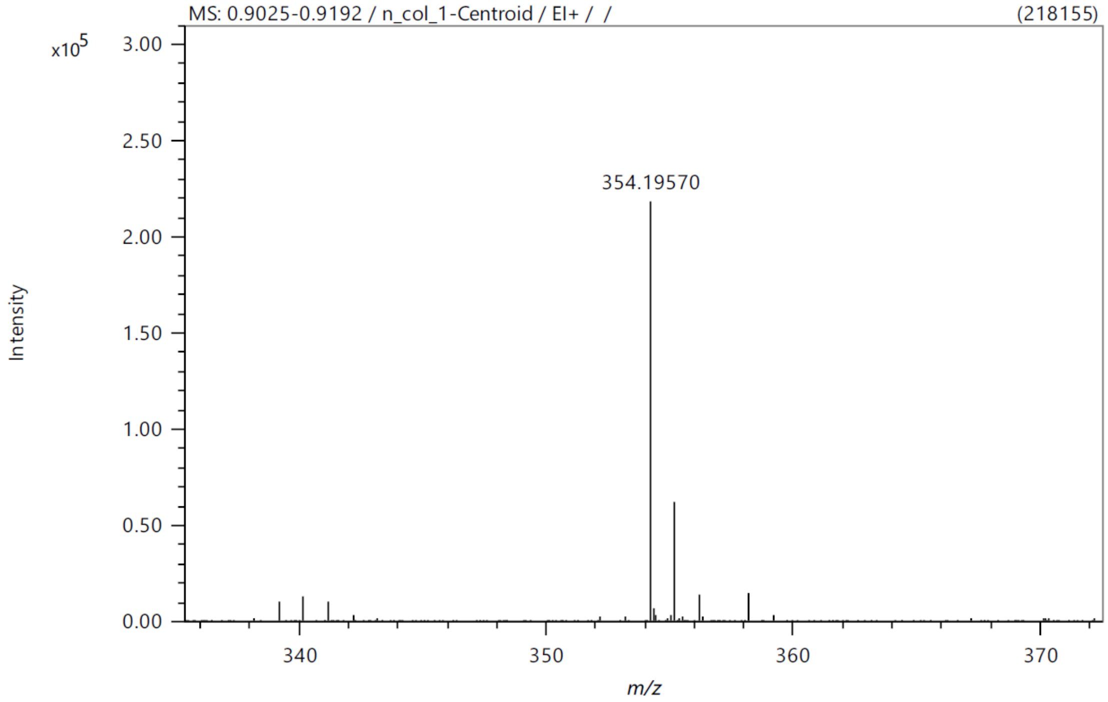

Figure S32 - Zoom in on the m/z peak at 354.19570 in the mass spectrum of **8,** [M]$^+$. Top, collected data. Bottom, calculated spectrum.

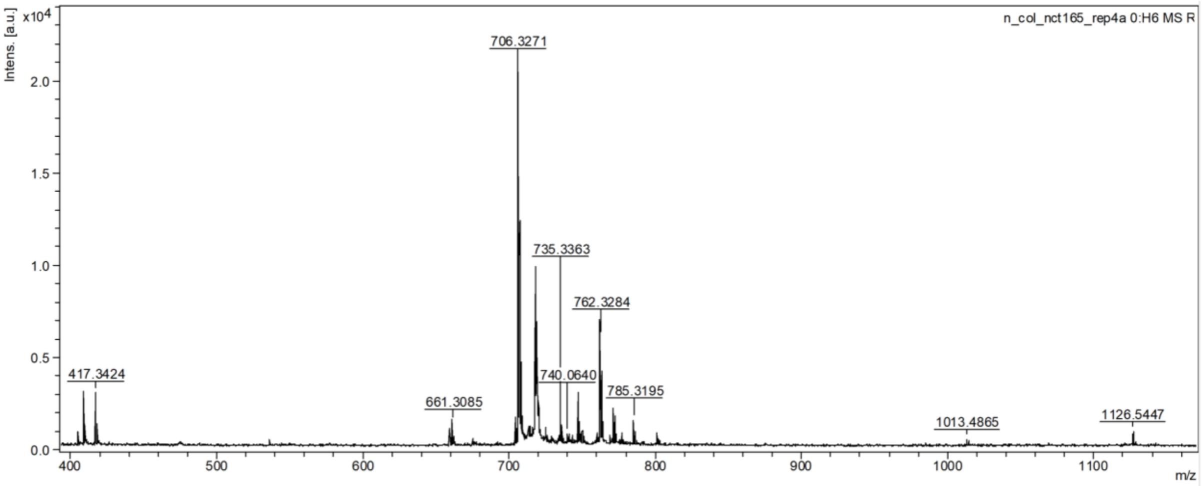

Figure S33 - Full mass spectrum for the mixture of **10** and **2^{Mes}**.

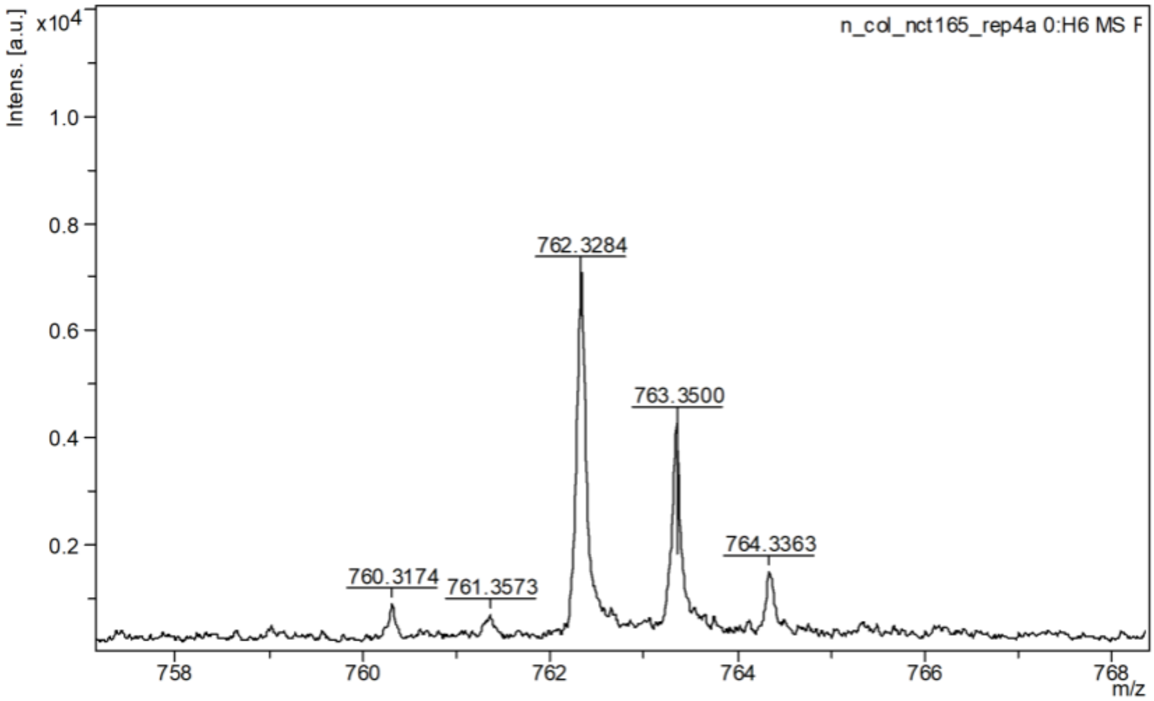

Figure S34 - Zoom in on the m/z peak at 762.3284 in the mass spectrum of **10**, $[M-2(CO)]^+$.

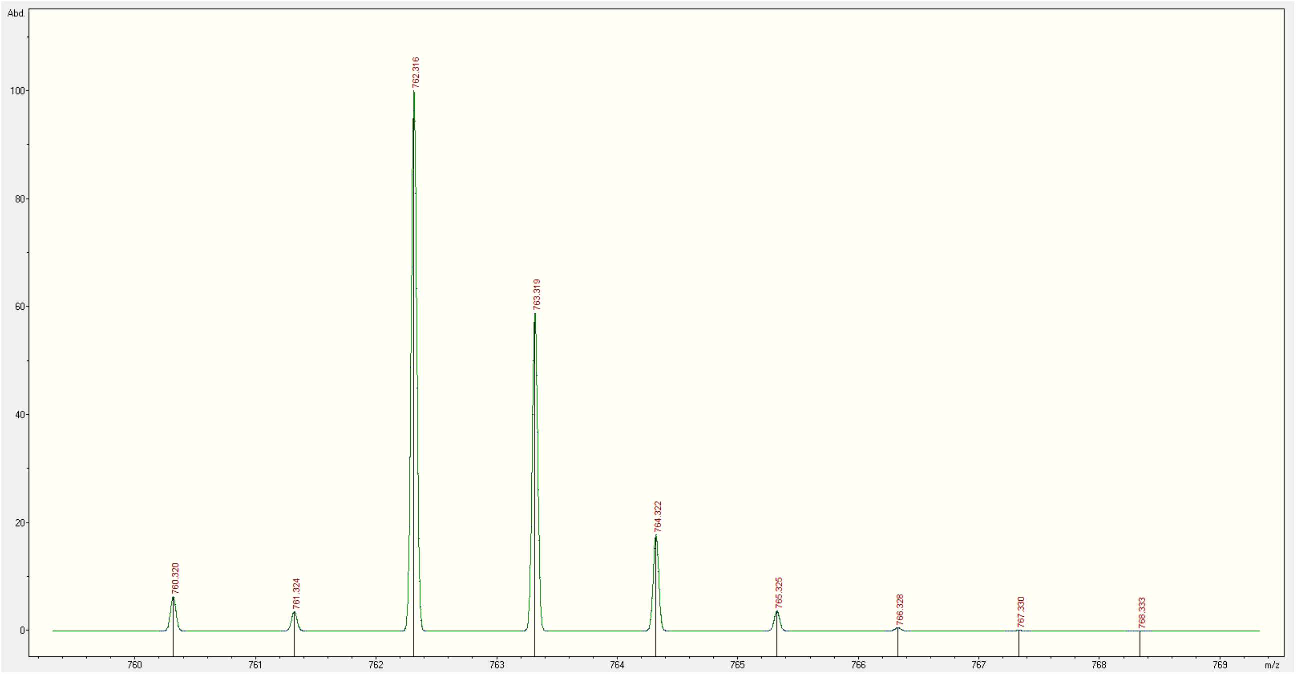

Figure S35 Calculated mass spectrum for $[M-2(CO)]^+$ of **10**.

3. Crystallographic information

The CCDC entries 2334282–2334296 contains the supplementary crystallographic data for this article. These data can be obtained free of charge from The Cambridge Crystallographic Data Centre *via* www.ccdc.cam.ac.uk/structures.

Table S1 - Selected crystallographic data

	$\mathbf{4^{Mes}\cdot Et_2O}$	$\mathbf{4^{Mes}}$	**5**	**6**
CCDC number	2334282	2334283	2334284	2334285
Empirical formula	$C_{57}H_{60}FeO_6$	$C_{53}H_{50}FeO_3$	$C_{52}H_{50}FeO_4$	$C_{52}H_{50}FeO_4$
Formula weight	896.90	822.78	794.77	794.77
Temperature/K	100(2)	120(2)	120.00(10)	120(2)
Crystal system	Triclinic	Triclinic	Monoclinic	Monoclinic
Space group	$P\bar{1}$	$P\bar{1}$	$P2_1/c$	$P2_1/m$
a/Å	12.19110(10)	12.3798(4)	12.1688(3)	10.9292(2)
b/Å	12.24350(10)	12.6107(4)	16.2148(5)	15.7523(3)
c/Å	32.9680(2)	14.6241(5)	21.5184(5)	12.3261(3)
α/°	93.1460(10)	86.062(3)	90	90
β/°	92.4990(10)	76.881(3)	91.955(2)	95.500(2)
γ/°	98.6370(10)	79.062(3)	90	90
Volume/Å^3	4851.01(5)	2182.32(13)	4243.42(19)	2112.29(8)
Z	4	2	4	2
Reflections collected	82643	38409	46376	9050
Independent reflections (R_{int})	29130 (0.0903)	8546 (0.0362)	8611 (0.0703)	4297 (0.0254)
R1 [I>=2σ (I)]	0.059	0.0345	0.0789	0.0607
wR_2 (all data)	0.1754	0.0892	0.2232	0.1730

	**7**	**10**	**11**	**12/13**
CCDC number	2334286	2334287	2334288	2334289
Empirical formula	$C_{51}H_{50}FeO_3$	$C_{54}H_{50}FeO_4$	$C_{52}H_{50}FeO_2$	$C_{156.5}H_{160.5}Fe_3O_{10}$
Formula weight	896.01	818.79	762.77	2368.89
Temperature/K	119.99(11)	99.95(10)	119.99(10)	120(2)
Crystal system	Triclinic	Monoclinic	Triclinic	Triclinic
Space group	$P\bar{1}$	$P2_1/c$	$P\bar{1}$	$P\bar{1}$
a/Å	11.0369(5)	11.4153(5)	10.4522(4)	13.2353(3)
b/Å	13.8006(5)	20.3383(9)	11.8004(4)	13.8441(5)
c/Å	18.0187(8)	18.6089(8)	16.7576(7)	36.1863(7)
α/°	73.812(3)	90	86.655(3)	79.731(2)
β/°	89.056(4)	90.572(4)	89.550(3)	83.226(2)
γ/°	72.173(4)	90	76.007(3)	72.453(3)
Volume/Å^3	2502.04(19)	4320.2(3)	2002.09(13)	6206.1(3)
Z	2	4	2	2
Reflections collected	18052	27247	10797	116054
Independent reflections (R_{int})	9606 (0.0393)	3901 (0.1390)	10797	21827 (0.0880)
R1 [I>=2σ (I)]	0.0508	0.0589	0.0941	0.1085
wR_2 (all data)	0.1402	0.1600	0.2726	0.3229

	14	2^{Mes}·nosolv1	2^{Mes}·nosolv2	2^{Mes}·C_6H_6
CCDC number	2334290	2334291	2334292	2334293
Empirical formula	$C_{77}H_{75}FeO_4$	$C_{52}H_{50}O_2$	$C_{52}H_{50}O_2$	$C_{55}H_{53}O_2$
Formula weight	1120.22	706.92	706.92	745.97
Temperature/K	120(2)	100(2)	120(2)	120(2)
Crystal system	Triclinic	Monoclinic	Orthorhombic	Orthorhombic
Space group	$P\bar{1}$	$P2_1/n$	$Pna2_1$	*Pbca*
a/Å	12.1282(3)	19.52240(10)	17.8916(6)	17.89460(10)
b/Å	14.9679(3)	20.01400(10)	16.8787(6)	20.08990(10)
c/Å	20.5823(5)	22.41850(10)	13.6585(5)	23.55470(10)
α/°	102.728(2)	90	90	90
β/°	99.634(2)	114.5960(10)	90	90
γ/°	107.973(2)	90	90	90
Volume/$Å^3$	3353.35(14)	8005.46(8)	4124.7(3)	8467.93(7)
Z	2	8	4	8
Reflections collected	26603	132890	38008	202923
Independent reflections (R_{int})	13307 (0.0228)	24396 (0.0421)	7224 (0.0613)	8791 (0.0415)
R1 [I>=2σ (I)]	0.0404	0.0626	0.0487	0.0475
wR_2 (all data)	0.1135	0.1948	0.1354	0.1232

	3^{Mes}·*i*Hex	3^{Mes}·CyH	3^{Mes}·$(H_2O)_4$
CCDC number	2334294	2334295	2334296
Empirical formula	$C_{106}H_{114}Fe_2O_8$	$C_{106}H_{112}Fe_2O_8$	$C_{124}H_{156.46}Fe_2O_{12.23}$
Formula weight	1627.67	1625.65	1954.32
Temperature/K	120(2)	120(2)	120(2)
Crystal system	Triclinic	Triclinic	Triclinic
Space group	$P\bar{1}$	$P\bar{1}$	$P\bar{1}$
a/Å	12.4176(2)	12.6674(3)	12.9566(2)
b/Å	13.2980(2)	13.2102(3)	13.7268(3)
c/Å	14.4695(2)	14.2164(3)	17.6044(4)
α/°	65.894(2)	67.027(2)	81.108(2)
β/°	83.350(2)	83.240(2)	70.509(2)
γ/°	84.359(2)	84.686(2)	70.199(2)
Volume/$Å^3$	2162.86(7)	2162.86(7)	2774.01(1)
Z	1	1	1
Reflections collected	54294	36898	62731
Independent reflections (R_{int})	8700 (0.0412)	8472 (0.0254)	11311 (0.0440)
R1 [I>=2σ (I)]	0.0449	0.0317	0.0482
wR_2 (all data)	0.1335	0.0899	0.1346

3.1 Additional refinement details

3.1.1 Further refinement details for 6

An attempt to improve the absorption correction for this compound was performed but led to no improvement. Platon TwinRotMat was used to search for potential twinning within the structure, but no twin law was observed. An attempt was made to model this atom as an additional Fe atom. When

allowed to freely refine, an occupancy of approx. 3% was found. No other atoms relating to this species could be found, and so this was not pursued further.

3.1.2 Further refinement details for 7

Complex **7** contained disordered solvent (*i*-hexane, 1.5 molecules per formula unit) which could not be modelled sensibly and was excluded from the electron density map using the solvent mask implemented in Olex2.[12]

3.1.3 Further refinement details for 10

The crystal diffracted weakly with a low-resolution diffraction limit; despite the use of synchrotron radiation. The data was truncated to a resolution of 1.05 Å. The truncation of the data resulted in a low data to parameter ratio of 7.17. The crystal also showed signs of powdering which further hindered data collection to high resolution. Attempts to isolate this compound over the course of 1 year have been made with various solvent combinations and crystallisation techniques. When crystals were finally obtained, they were part of a 50:50 mix of the desired structure and a squaraine ($\mathbf{2}^{\mathbf{Mes}}$), another product from this reaction. As both compounds are the same colour and have similar morphologies, it is difficult to separate the two compounds, in addition to their air and moisture sensitivity.

All hydrogen atoms were geometrically placed and refined using a riding model.

3.1.4 Further refinement details for 11

Integrated and refined as a 3-component twin. The 3-components were found using the twin finding tool in CrysalisPro.

3.1.5 Further refinement details for 12/13

The crystal consists of 1 eq. of **12** and 0.5 eq. of **13** per asymmetric unit.

Diffraction from the air sensitive crystal was weak at high angle with I/sig dropping below a value of 3 above a resolution of 0.9 Å.

Residual electron density peaks of 1.53 and 1.18 e $Å^{-3}$ are observed 0.99 and 2.04 Å away from iron atoms Fe1 and Fe3 respectively. These peak are assumed to be caused by unmodelled disorder likely involving scrambling of the mixture of ligands already present in the two complexes. Attempts to model the disorder were unsuccessful. The presence of twinning was not indicated by PLATON TwinRotMat.

To aid refinement of the weak data with underlying unmodelled suspected disorder, rigid bond and similarity restraints were applied to the anisotropic displacement parameters of all atoms in the structure (RIGU, SIMU). The anisotropic displacement parameters of carboxylate moiety C128 were restrained to be more isotropic (ISOR). The phenyl-carboxylate C-C bond of carboxylate moiety C128 was restrained to have a distance of 1.5 Å (DIFX, esd 0.02 Å). The atoms of carboxylate moiety C128 and the connected phenyl carbon atoms were restrained to be planar (FLAT). The oxygen-oxygen distances of all carboxylate moieties in the structure were restrained to be similar (SADI).

3.1.6 Further refinement details for 14

13 shows disorder about the cyclobutenone core with the disordered components freely refining to sum unity with values of 0.46(1) and 0.54(1), respectively. To model the disorder, geometric similarity restraints were applied to all chemically identical 1,2 and 1,3 distances of the two disordered components (SAME). Bonds C4A-C3A and C4B-C3B were restrained to a distance of 1.56 Å based on similar moieties with the CCDC (DFIX, esd 0.02 Å). Bonds C3A-C2A and C3B-C2B were restrained to a distance of 1.53 Å bas ed on similar moieties in the CCDC (DFIX, esd 0.02 Å). Same distance restraints were employed to the following C-Ar bonds C6A C11, C6B C41, C41 C3A, C3B C11 (SADI). Rigid bond and similarity restraints were applied to all isotropic and anisotropic displacement parameters in the structure (RIGU, SIMU). The oxygen bound to the Fe (O1A/O1B) was split but constrained to have the same coordinates (EXYZ) and thermal parameters (EADP).

All hydrogen atoms except were observed in the electron density map before being geometrically placed and refined using a riding model.

Disordered solvent molecules could not be sensibly modelled and were excluded from the electron density map using the solvent mask implemented in Olex2.[12] A total of 101 electrons were accounted from the $\bar{P}1$ cell equating to 1 *iso*-hexane molecule (C6H14) per asymmetric unit, which have been included in the unit cell contents and calculation of derived parameters.

3.1.7 Further refinement details for 3^{Mes}·*i*Hex

FENCLO contained disordered solvent (*iso*-hexane, 1 molecule per formula unit) which could not be modelled sensibly and was excluded from the electron density map using the solvent mask implemented in Olex2.[12]

3.1.8 Further refinement details for $3^{Mes}\cdot(H_2O)_4$

Structure contains a partially occupied H_2O bridging two bound H_2O molecules. The occupancy was allowed to refine freely and settled on an occupancy of 11% per asymmetric unit.

The cyclohexane solvent that co-crystalised were both disordered. The occupancies were allowed to refine freely. Thermal restraints were used on all disordered atoms (RIGU, SIMU). Same distance restraints were also used (SADI).

3.2 $4^{Mes}\cdot Et_2O$

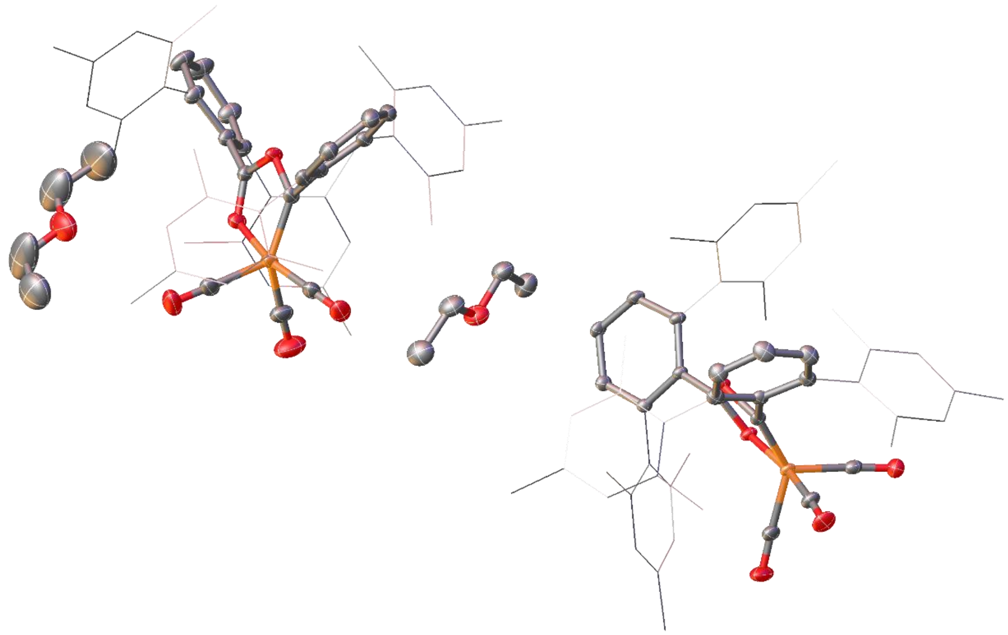

Figure S36 – Asymmetric unit for $\mathbf{4^{Mes}\cdot Et_2O}$. Protons omitted for clarity. Mesityl groups shown as wireframes. Thermal ellipsoids given at the 50% level.

Table S2 – Comparison of the bond lengths obtained for $\mathbf{4^{Mes}}$ and $\mathbf{4^{Mes}\cdot Et_2O}$.

	4^{Mes}	$4^{Mes}\cdot Et_2O$	
**Fe–C$_{carbene}$**	Fe1–C1; 1.8395(14) Å	Fe1–C1; 1.8362(16) Å	Fe2–C54; 1.839(2) Å
**Fe–O**	Fe1–O1; 1.9470(11) Å	Fe1–O1; 1.9686(11) Å	Fe2–O6; 1.9508(11) Å
**Fe–C$_{carbonyl}$**	Fe1–C51; 1.7549(16) Å<br>Fe1–C52; 1.828(2) Å<br>Fe1–C53; 1.8382(19) Å	Fe1–C51; 1.8297(19) Å<br>Fe1–C52; 1.8373(17) Å<br>Fe1–C53; 1.7484(16) Å	Fe2–C104; 1.816(2) Å<br>Fe2–C105; 1.836(3) Å<br>Fe2–C106; 1.7521(19) Å
**C$_{carbene}$–Fe–O**	C1–Fe1–O1 80.81(5) Å	C1–Fe1–O1 81.81(5)°	C54–Fe2–O6 82.02(7)°
**O–C–O (carboxyl)**	O1–C2–O2; 118.77(13) Å	O1–C2–O2; 119.03(13)°	O7–C55–O6; 119.42(15)°

3.3 Squaraine (2^{Mes}) solvatomorphs

3.3.1 No solvent (from *i*-hexane)

Crystals were isolated from a concentrated Et_2O solution of $\mathbf{2}^{Mes}$ stored in a freezer at −30 °C.

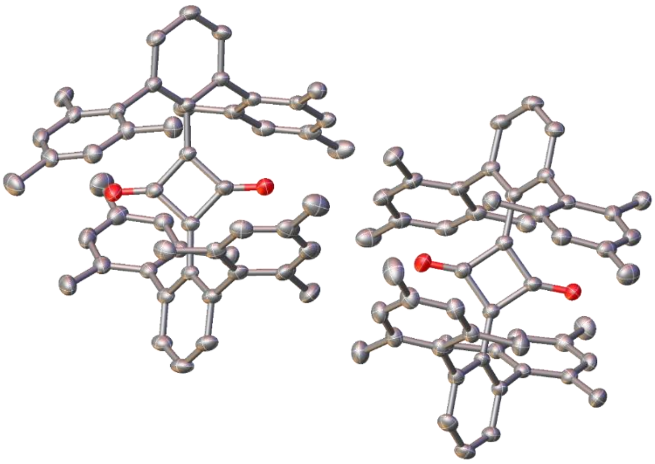

Figure S37 – Asymmetric unit of $\mathbf{2}^{Mes}$. Protons omitted for clarity. Thermal ellipsoids given at the 50% level.

3.3.2 No solvent (from Et_2O)

Crystals were isolated from a concentrated Et_2O solution of $\mathbf{2}^{Mes}$ stored in a freezer at −30 °C.

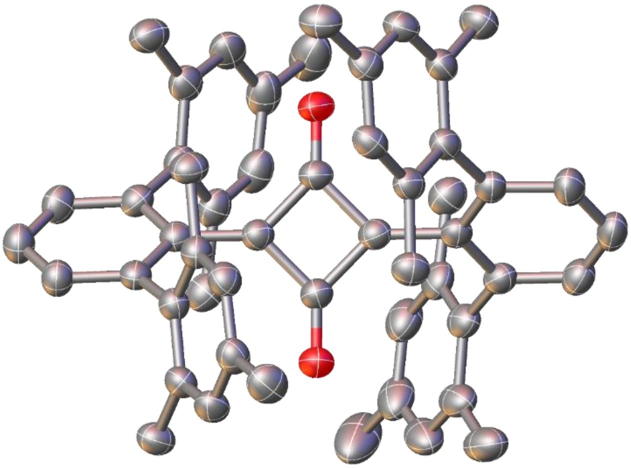

Figure S38 – Asymmetric unit $\mathbf{2}^{Mes}$. Protons omitted for clarity. Thermal ellipsoids given at the 50% level.

3.3.3 C_6H_6 solvate

Crystals were isolated from a concentrated C_6H_6 solution of **2^{Mes}** at room temperature.

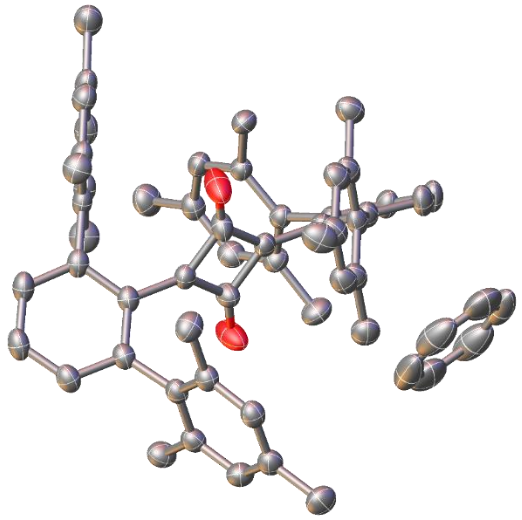

Figure S39 - Asymmetric unit **2^{Mes}·C_6H_6**. Protons omitted for clarity. Thermal ellipsoids given at the 50% level.

3.4 Carboxylate (3^{Mes}) solvatomorphs/derivatives

3.4.1 *iso*-Hexane solvatomorph

Crystals were isolated from a reaction of **4^{Mes}** (100 mg, 0.15 mmol) under an atmosphere of CO in *i*-hexane without stirring. Large pale yellow/colourless crystals of **3^{Mes}·*i*Hex** were obtained over the course of 7 days.

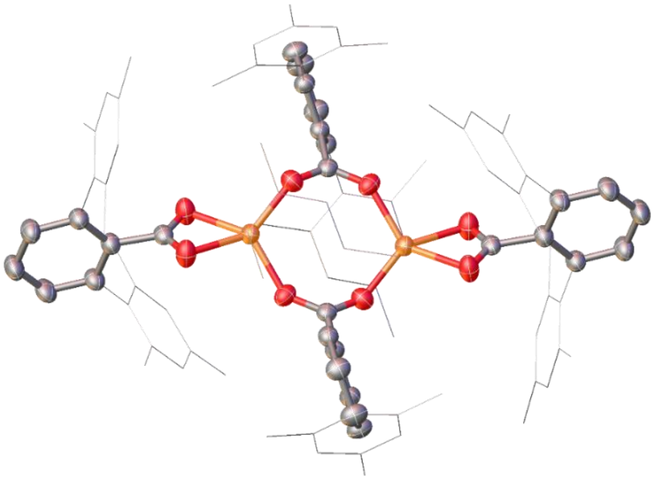

Figure S40 – Unit cell for **3^{Mes}·*i*Hex**. Protons omitted for clarity. Disordered *iso*-hexane removed during processing with a solvent mask. Mesityl groups shown as wireframes. Thermal ellipsoids given at the 50% level.

3.4.2 Cyclohexane solvatomorph

Crystals were isolated from a reaction of **4^Mes^** (100 mg, 0.15 mmol) under an atmosphere of CO in cyclohexane without stirring. Large pale yellow/colourless crystals of **3^{Mes}·CyH** were obtained over the course of 7 days.

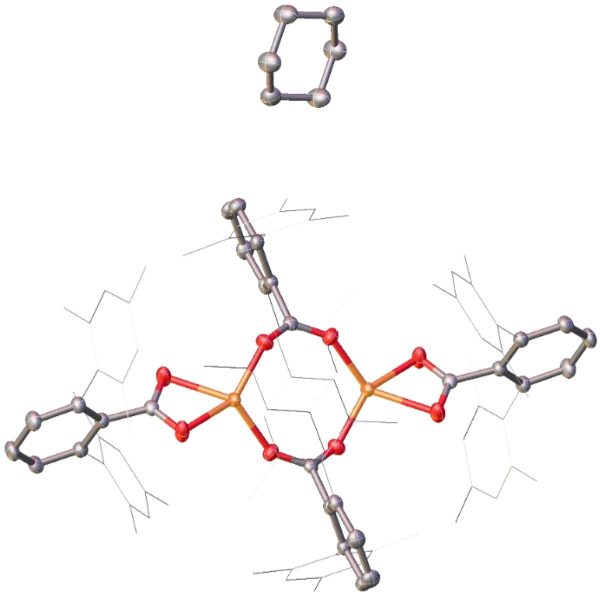

Figure S41 - Unit cell for **3^{Mes}·CyH**. Protons omitted for clarity. Mesityl groups shown as wireframes. Thermal ellipsoids given at the 50% level.

3.4.3 Water complex of 3^{Mes}

Crystals were isolated from a reaction of **4^{Mes}** under an atmosphere of CO in cyclohexane in a Harrick cell. Over 2 weeks, moisture slowly diffused into the cell and **3^{Mes}·$(H_2O)_4$** was obtained as colourless crystals.

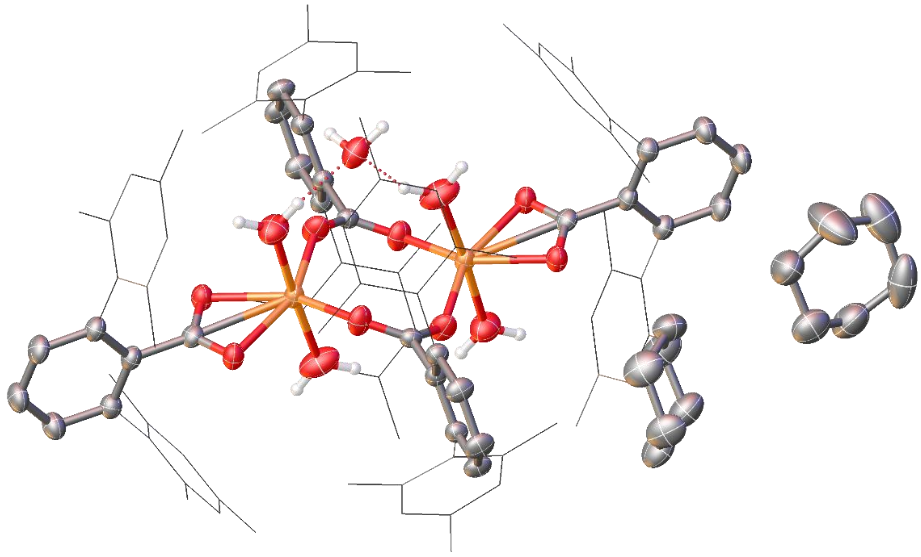

Figure S42 - Unit cell for **3^{Mes}·$(H_2O)_5$**. Protons, except those bound to water molecules, omitted for clarity. One part of disordered cyclohexane shown. Mesityl groups shown as wireframes. Thermal ellipsoids given at the 50% level.

4. Reaction monitoring details

4.1 Pre carbene formation

4.1.1 ReactIR monitoring

A flask was charged with **1^{Mes}** (161 mg, 0.24 mmol) in the glovebox and dissolved in toluene (5 mL). The flask was connected to the Schlenk line under a flow of argon and the ReactIR probe (Si) inserted. A background was taken in the headspace of the reaction flask before lowering the probe into the solution. IR spectra were then periodically recorded every 2 minutes. The first spectra of **1^{Mes}** in toluene were collected twice to ensure that the spectrometer was working effectively and equilibrated. After 5 minutes, CO was purged through the flask by lifting the Teflon seal attached to the probe, creating a gap to allow the CO to flow through the flask. Uptake of CO into the solution can be observed visually by a colour change from yellow to orange and CO stretched started to form between 2200-1600 cm^{-1}. To clearly see the peaks, solvent suppression was performed using the iC IR software (v7.1, Mettler Toledo). This was performed by obtaining a spectrum of the solvent used (toluene) and using the built-in feature to subtract the signals relating to the solvent.

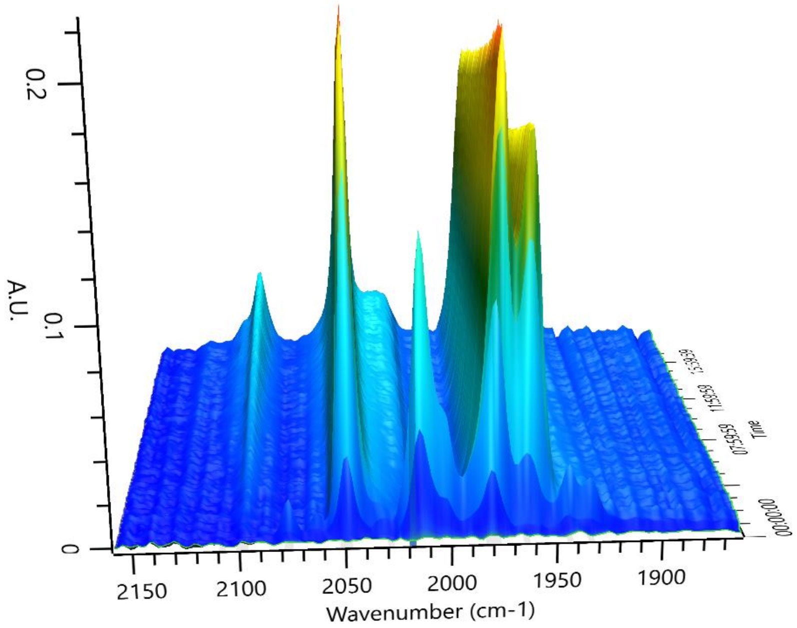

Figure S43 - Graph taken from the iC IR software (ReactIR) for the reaction monitoring of **1^{Mes}** with CO in toluene. Three-dimensional plot showing the IR spectra as a function of time from 0 to 17 h 17 min. The large peak at 2098 cm^{-1} indicates that degradation of the reaction has occurred (ketene formation).

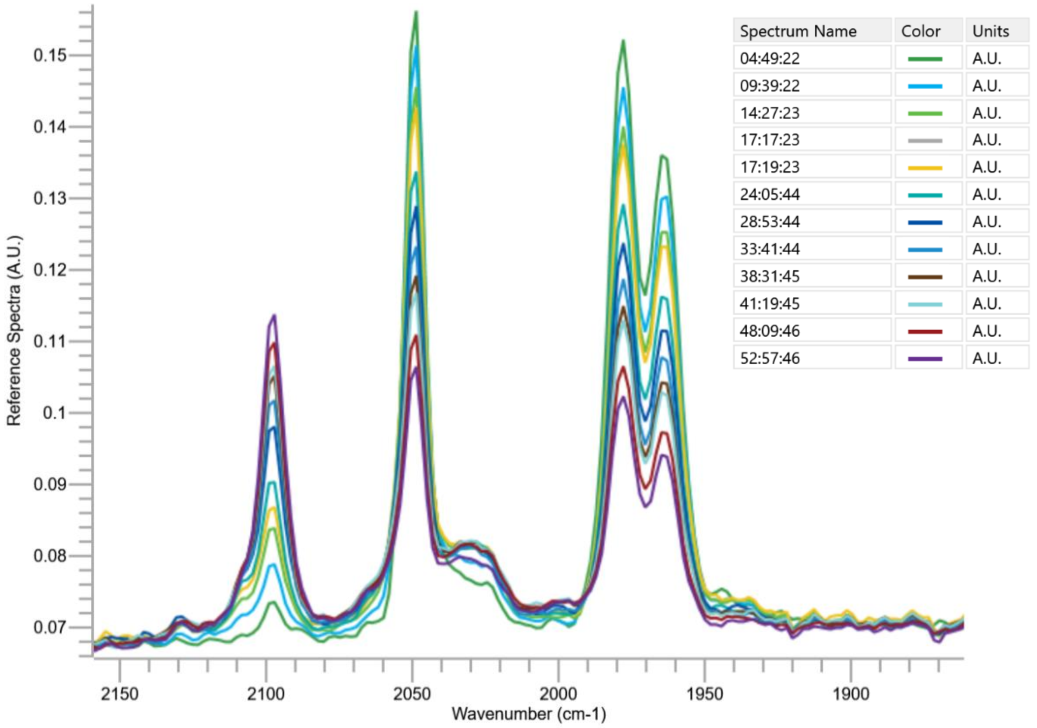

Figure S44 - Graph taken from the iC IR software (ReactIR) for the reaction monitoring of **1^Mes^** with CO in toluene. Overlaid IR spectra from 4 h 50 mins to 17 h 17 min. The large peak at 2098 cm^{-1} indicates that degradation of the reaction has occurred (ketene formation).

4.1.2 *In situ* ^{1}H NMR monitoring

1^{Mes} (14.7 mg , 0.02 mmol) was dissolved in C_6D_6 (0.6 mL) in a J Young NMR tube in a glovebox. The NMR tube was placed under vacuum on a Schlenk line to remove the argon from the tube and then sealed under a static vacuum. The sample was then placed under an atmosphere of CO and vigorously shaken to make sure some of the gas saturated the solvent. The sample was then placed back under vacuum and refilled with CO again. The sample was then placed in the NMR spectrometer and ^{1}H spectra periodically recorded.

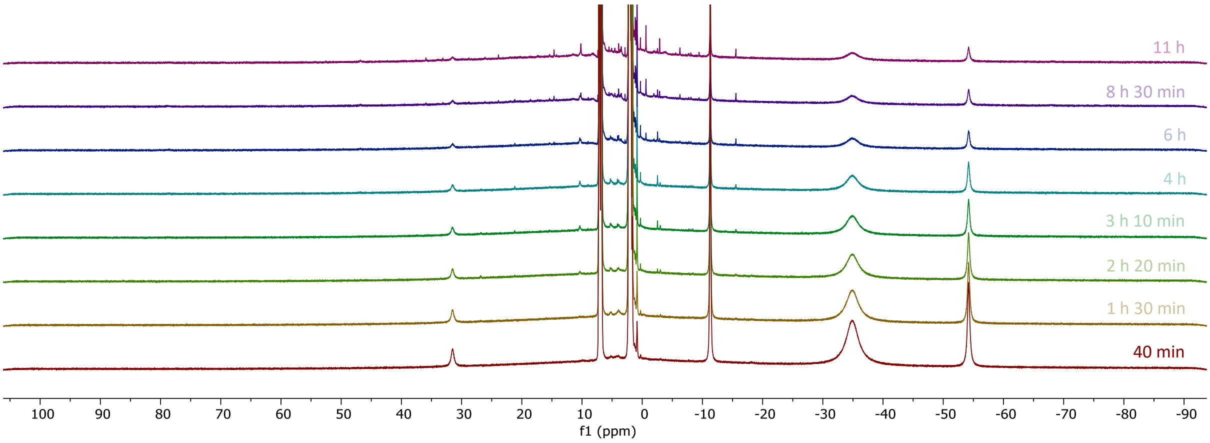

Figure S45 – ^{1}H spectrum in C_6D_6 at 25°C from +106 to −93 ppm for the in situ monitoring of **1^{Mes}** with CO for the first 11 hours. Paramagnetic **1^{Mes}** is still present.

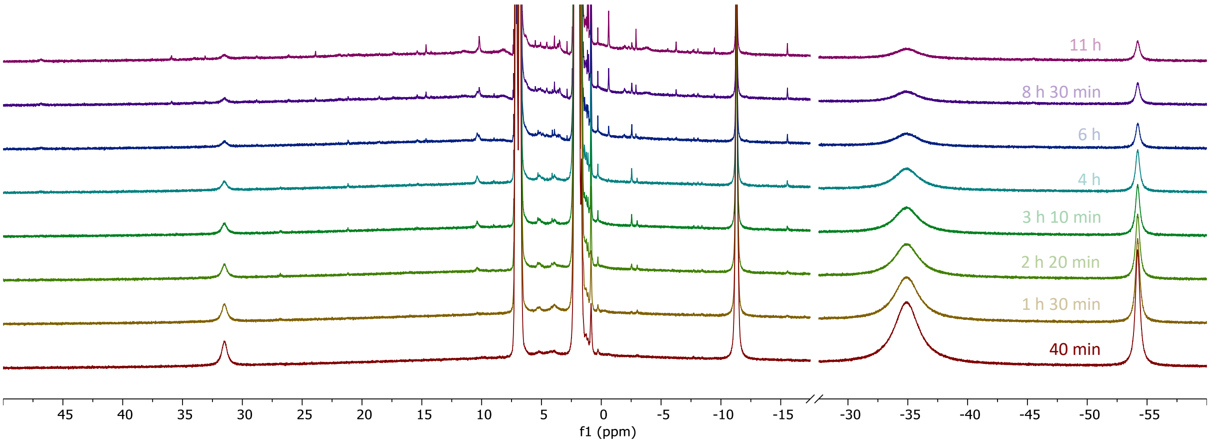

Figure S46 – Zoomed in ^{1}H spectrum in C_6D_6 at 25°C from +50 to −650 ppm for the in situ monitoring of $\mathbf{1}^{\mathbf{Mes}}$ with CO for the first 11 hours showing that paramagnetic $\mathbf{1}^{\mathbf{Mes}}$ is still present. 17.5-27.5 ppm has been trimmed due to the absence of peaks to allow for better visualisation of the relevant peaks. New paramagnetic signals are seen from 2 hours 20 minutes.

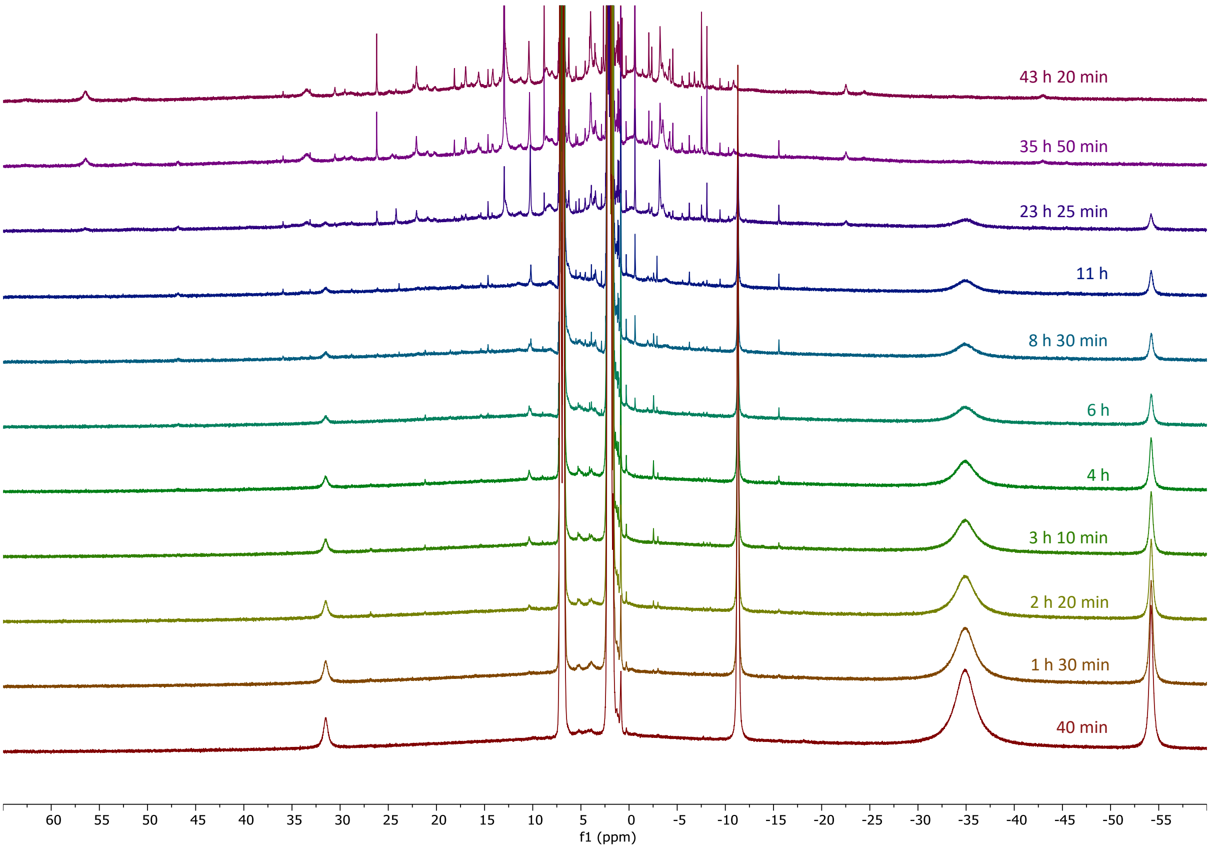

Figure S47 – ^{1}H NMR spectrum in C_6D_6 at 25°C from +70 to −60 ppm for the in situ monitoring up to 40.5 h into the reaction. After ~36 h $\mathbf{1}^{\mathbf{Mes}}$ is consumed. Paramagnetic species are observed to form and be consumed during this timeframe.

4.1.3 *In situ* $^{13}C\{^{1}H\}$ NMR monitoring

$\mathbf{1}^{\mathbf{Mes}}$ (34 mg , 0.04 mmol) was dissolved in C_6D_6 (0.6 mL) in a J young NMR tube in a glovebox. The NMR tube was connected to the Schlenk line *via* a three-way tap which was also connected to a cylinder of ^{13}CO. The system was purged under vacuum and backfilled with argon 3 times. On the third vacuum cycle, the headspace of the NMR tube was carefully removed, and the tube sealed under a static vacuum. The three-way tap was the closed to the Schlenk line and the tubing filled with ^{13}CO up to a pressure of 1 atm. The ^{13}CO is then introduced to the NMR tube which was shaken to allow some of

the CO react. The tube was then opened again to replenish the CO taken up by the complex. The tube was sealed under an atmosphere of ^{13}CO. The sample was then placed in the NMR spectrometer and ^{1}H and ^{13}C{^{1}H} spectra periodically recorded. It was noted after *ca.* 1 h that the reaction did not appear to proceed further and so more ^{13}CO was added to the NMR tube before returning it to continue monitoring.

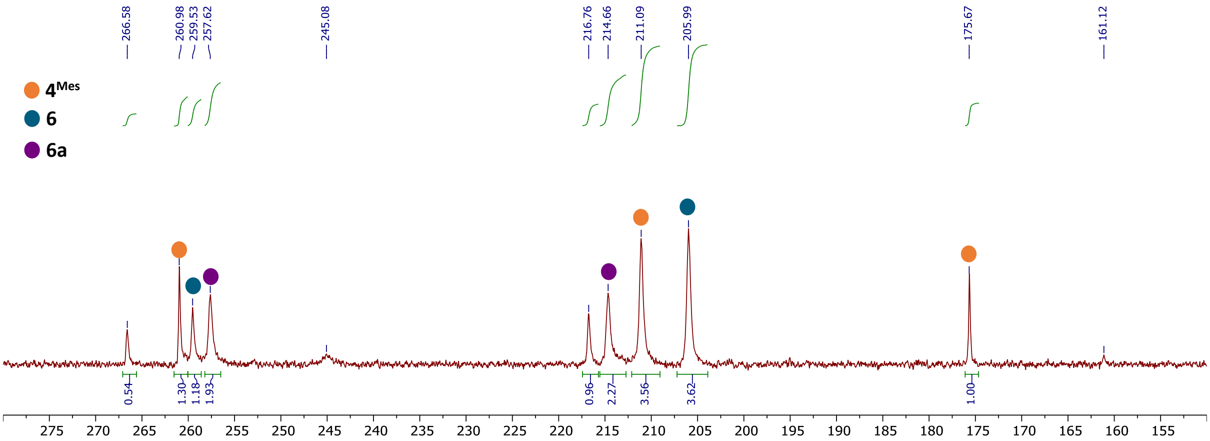

Figure S48 – ^{13}C{^{1}H} spectrum at 25°C of the reaction between **1^{Mes}** and ^{13}CO, 15 minutes after addition of the ^{13}CO. Peaks relating to **4^{Mes}** highlighted with orange circles, **6** with turquoise circles and **6a** with purple circles. Unknown peaks still present.

4.2 Post carbene formation

4.2.1 End of reaction starting from 1^{Mes}

A J Young ampoule was charged with **1^{Mes}** (150 mg, 0.23 mmol) in the glovebox and dissolved in toluene (5 mL). The flask was connected to the Schlenk line and the inert gas removed under vacuum, with care taken to not remove any solvent. The flask was backfilled with CO and the vacuum/refilling process was repeated two more times. The reaction was left for 8 days before filtering through a Teflon canula fitted with glass fibre paper. The reaction solution was analysed, before distilling the volatiles and collecting the IR spectrum of the distillate.

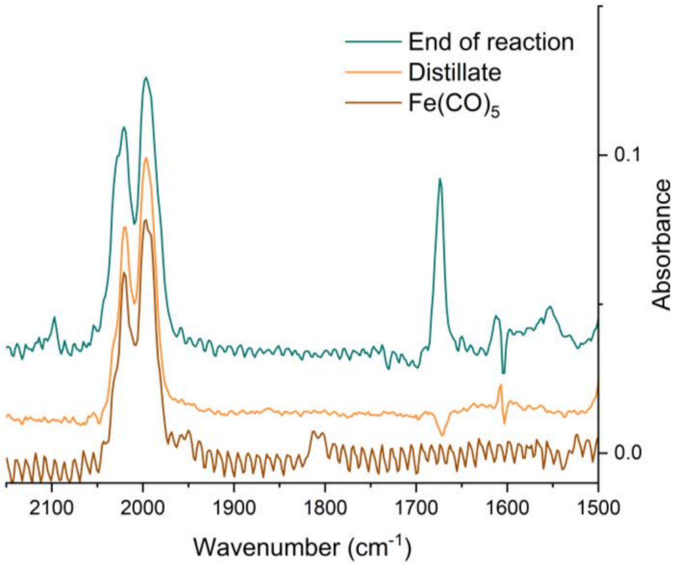

Figure S49 - IR spectra collected on ReactIR of the reaction between **1^{Mes}** and CO in toluene after 7 days. a) Turquoise, spectrum after the reaction has reached completion. b) Orange, distillate of the reaction. c) Brown, spectrum of $Fe(CO)_5$ in toluene.

4.2.2 End of reaction starting from 4^{Mes}

A J Young ampoule was charged with **4^{Mes}** (300 mg, 0.36 mmol) in the glovebox and dissolved in toluene (12 mL). The flask was connected to the Schlenk line and the inert gas removed under vacuum, being careful to not remove any solvent. The flask was backfilled with CO and the vacuum/refilling process was repeated two more times. The reaction was left for 8 days before filtering through a Teflon canula fitted with glass fibre paper. The reaction solution was analysed, before distilling the volatiles and collecting the IR spectrum of the distillate. The solid residue was also redissolved in toluene and the IR spectrum of the non-volatile components obtained.

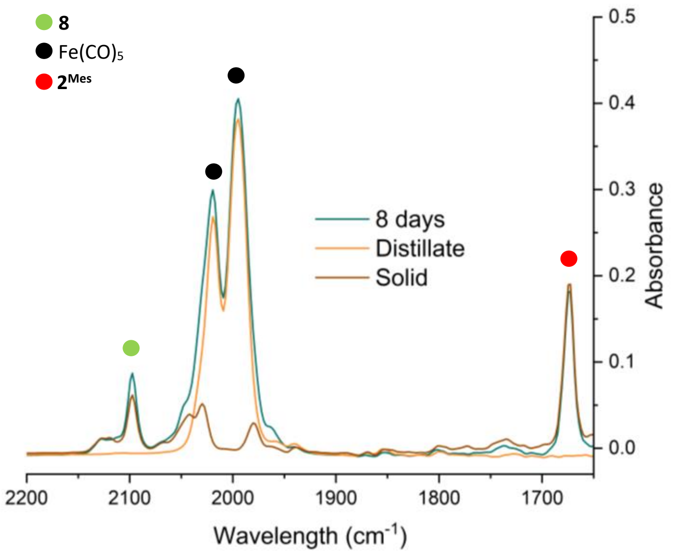

Figure S50 - IR spectra collected in a Harrick cell of the reaction between **4^{Mes}** and CO in toluene after 8 days. a) Turquoise, spectrum after the reaction has reached completion. b) Orange, distillate of the reaction. c) Brown, species present in the residual solid after vacuum.

4.2.3 IR spectra by reaction sampling, toluene

A J Young ampoule was charged with **4^{Mes}** (300 mg, 0.36 mmol) in the glovebox and dissolved in toluene (12 mL). The flask was connected to the Schlenk line and the inert gas removed under vacuum, with care taken to not remove any solvent. The flask was backfilled with CO and the vacuum/refilling process was repeated two more times. The reaction was then left to react for ~22 hours and after this time the flask was transferred to the glovebox. 0.7 mL of solution was then removed from the ampoule and filtered through a pipette plugged with glass fibre paper. The filtered solution was then transferred into the Harrick cell which was then sealed under an inert atmosphere. The Harrick cell could then be removed from the glovebox once sealed, and the data collected. Further samples were then taken approximately every 24 hours for the following three days. A further data point was collected on the ninth day of reaction.

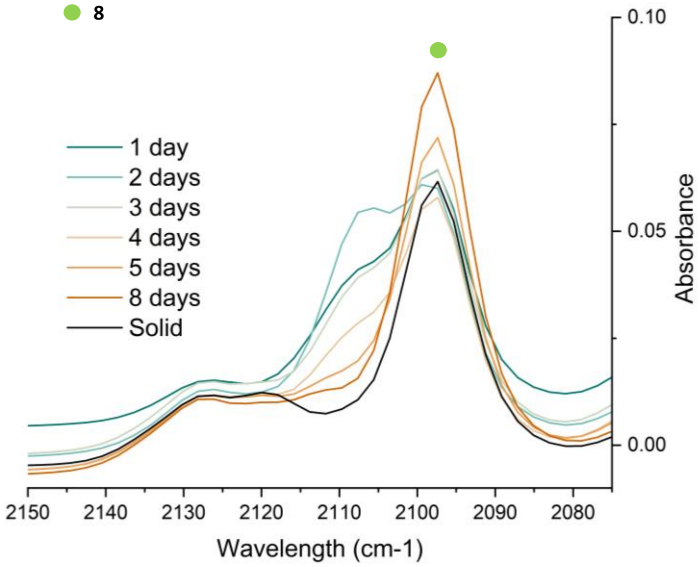

Figure S51 – Zoom in of the ketene region for the IR spectra collected in a Harrick cell of the reaction between **4^{Mes}** and CO in toluene after 8 days. The colour gradient goes from dark turquoise (earliest) to dark orange (latest). Signal for **8** marked in green.

4.2.4 IR spectra by reaction sampling, cyclohexane

A J Young ampoule was charged with **4**^{Mes} (250 mg, 0.28 mmol) in the glovebox and dissolved in cyclohexane (10 mL). 0.7 mL of the solution was sealed in a Harrick cell to obtain a spectrum representative of the start of the reaction. The ampoule was removed from the glovebox and connected to the Schlenk line. The inert gas was removed under vacuum, with care taken to not remove any solvent. The flask was backfilled with CO and the vacuum/refilling process was repeated two more times. The reaction was then left to react for ~22 hours and after this time the flask was transferred to the glovebox 0.7 mL of solution was then removed from the ampoule and filtered through a pipette plugged with glass fibre paper. The filtered solution was then transferred into the Harrick cell and sealed under an inert atmosphere. The Harrick cell was then removed from the glovebox once sealed and the data collected. Further samples were then taken approximately every 24 hours for the following three days. A further data point was collected on the ninth day of reaction.

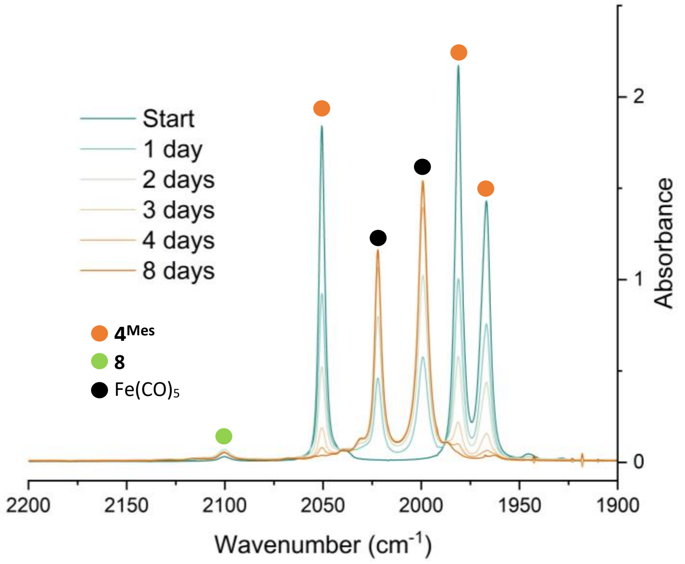

Figure S52 - IR spectra collected in a Harrick cell of the reaction between **4**^{Mes} and CO in cyclohexane over 8 days. The colour gradient goes from dark turquoise (earliest) to dark orange (latest).

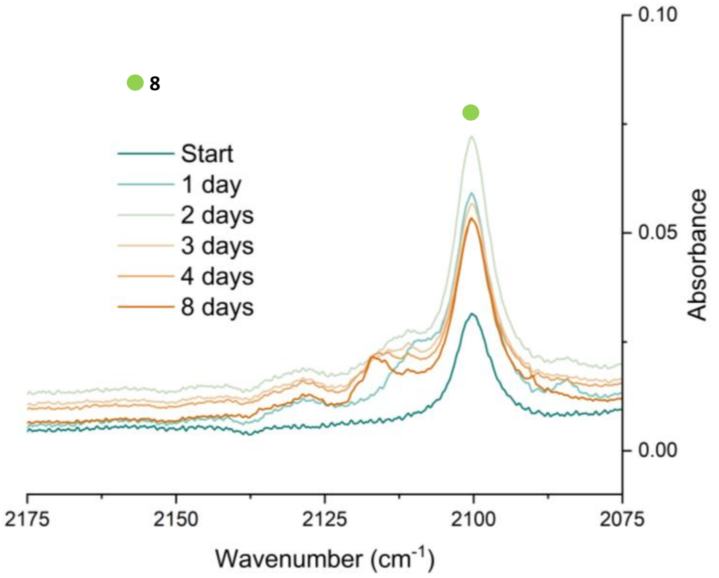

Figure S53 – Zoom in of the ketene region of an IR spectra collected in a Harrick cell for the reaction between **4**^{Mes} and CO in cyclohexane over 8 days. The colour gradient goes from dark turquoise (earliest) to dark orange (latest).

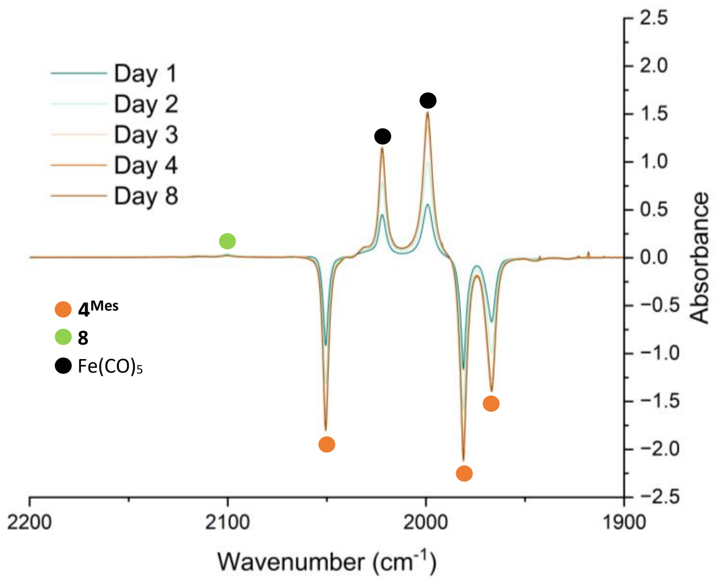

Figure S54 - IR spectra collected in a Harrick cell of the reaction between **4^{Mes}** and CO in cyclohexane over 8 days. Start spectra (Figure S40) has been subtracted from days 1-8. Positive peaks indicate formation while negative peaks indicate consumption. The colour gradient goes from dark turquoise (earliest) to dark orange (latest).

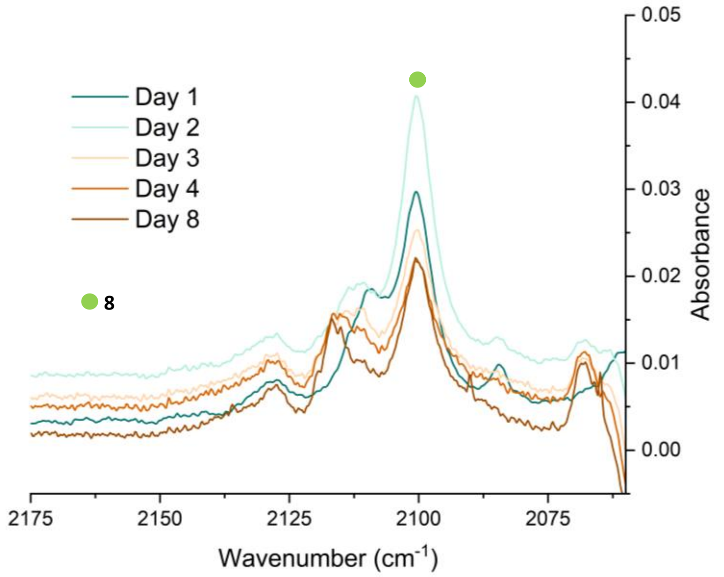

Figure S55 – Zoom in on the ketene region of the IR spectra collected in a Harrick cell of the reaction between **4^{Mes}** and CO in cyclohexane over 8 days. Start spectra (Figure S40) has been subtracted from days 1-8. Positive peaks indicate formation while negative peaks indicate consumption. The colour gradient goes from dark turquoise (earliest) to dark orange (latest).

4.2.5 NMR spectra by reaction sampling

A J Young ampoule was charged with **4^{Mes}** (300 mg, 0.36 mmol) in the glovebox and dissolved in C_6H_6 (12 mL). The flask was connected to the Schlenk line and the inert gas removed under vacuum, with care taken to not remove any solvent. The flask was backfilled with CO and the vacuum/refilling process was repeated twice more. The reaction was then left to react for ~22 hours and after this time the flask was transferred to the glovebox. ~0.7 mL of solution was then removed from the ampoule and filtered through a pipette plugged with glass fibre paper. The filtered solution was then transferred into the J Young NMR tube which is then sealed under an inert atmosphere. NMR spectra are collected in on a Bruker AV500 with the lock turned off. Samples are shimmed against the signal for C_6H_6 using topshim 1H lockoff o1p=# selwid=0.5, where # is the chemical shift (ppm) of the middle

of the C_6H_6 signal in a spectrum obtained prior to shimming. The ampoule containing the remaining reaction solution was then refreshed on the Schlenk line with more CO to ensure a sufficient quantity of CO is present. Further samples were then taken approximately every 24 hours for the following four days. A further data point was collected on the eighth day of reaction.

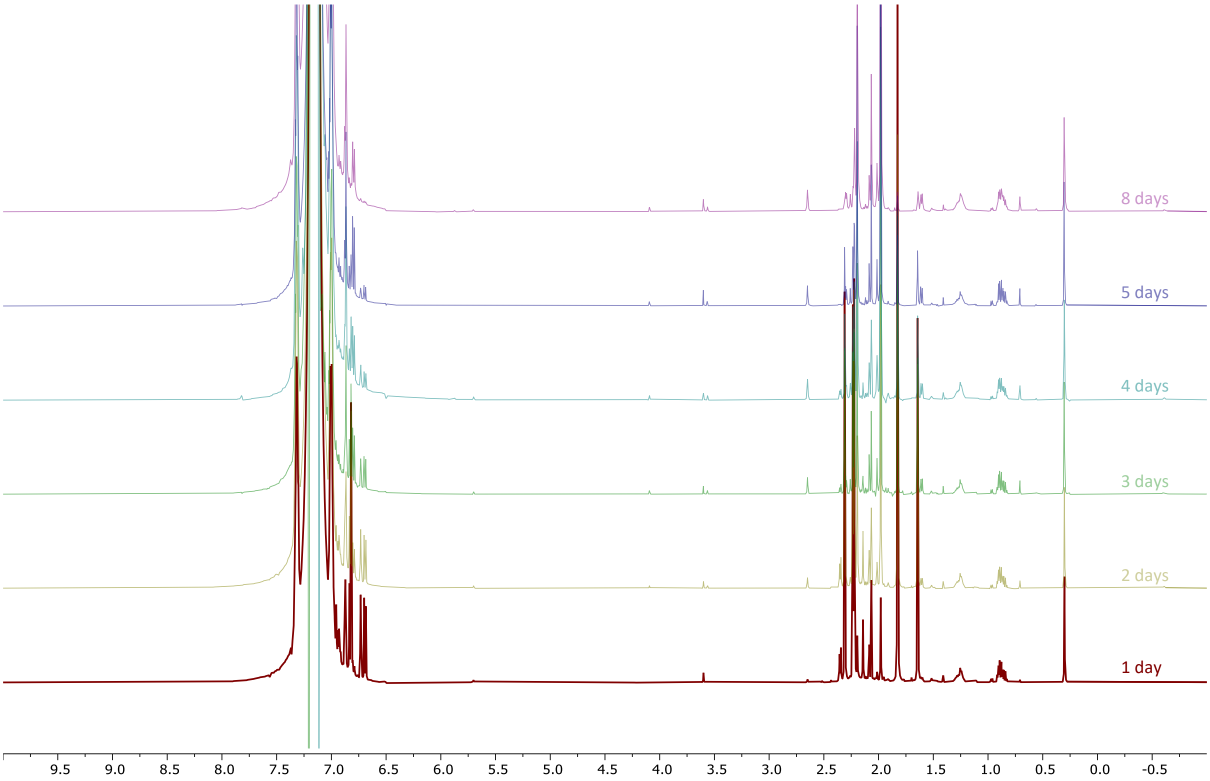

Figure S56 - ^{1}H NMR spectrum at 25°C of the reaction between $\mathbf{4^{Mes}}$ and CO in C_6H_6 from 10 - −1 ppm. This is the full spectrum view of Figure 8 in the manuscript.

4.2.6 *In situ* monitoring by EPR spectra

A quartz EPR tube fitted with a stopcock and ground glass joint was charged with **4**^{Mes} (35 mg, 0.04 mmol) in the glovebox and dissolved in toluene (0.6 mL). The tube was connected to the Schlenk line and the inert gas removed under vacuum, with care taken to not remove any solvent. The flask was backfilled with CO and the vacuum/refilling process was repeated twice more. The reaction was monitored periodically over the course of 15 days.

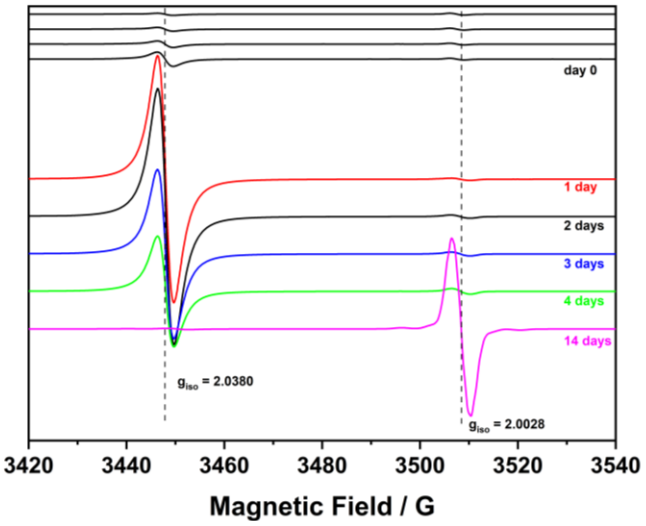

Figure S57 - EPR spectra recorded at 25°C of the reaction between **4**^{Mes} and CO in toluene over 14 days. Day 0 measurements started ~30 minutes after setting up the reaction.

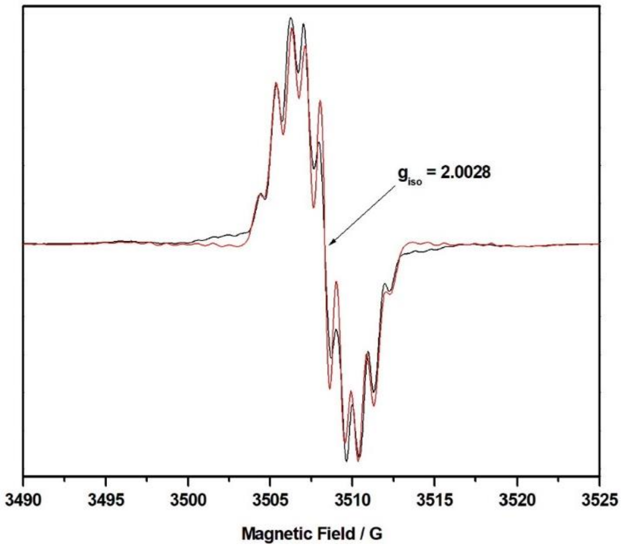

Figure S58 - Experimental X-band EPR spectrum of [**2**^{Mes}]$^{\cdot -}$ formed by the reaction of **4**^{Mes} under an atmosphere of natural abundance CO (black line) recorded at ambient temperature as a solution in toluene. The red line represents a simulation of the experimental spectrum using parameters given in **Table S3**.

4.2.6 Frozen EPR spectra

A J Young ampoule was charged with **4**^{Mes} (200 mg, 0.3 mmol) in the glovebox and dissolved in toluene (8 mL). The flask was connected to the Schlenk line and the inert gas removed under vacuum, with care taken to not remove any solvent. The flask was backfilled with CO up to a pressure of ~1 atm. The reaction was then left to react for 24 hours. After this time the flask was transferred to the glovebox. ~0.5 mL of solution was then removed from the ampoule and filtered through a pipette plugged with

glass fibre paper. The filtered solution was then transferred into an EPR tube and sealed under an inert atmosphere. A room temperature EPR data collection was conducted to confirm the presence of the signal at g_{iso} = 2.00. The sample was then carefully frozen in liquid nitrogen and a second EPR spectrum (shown below) collected at −196 °C.

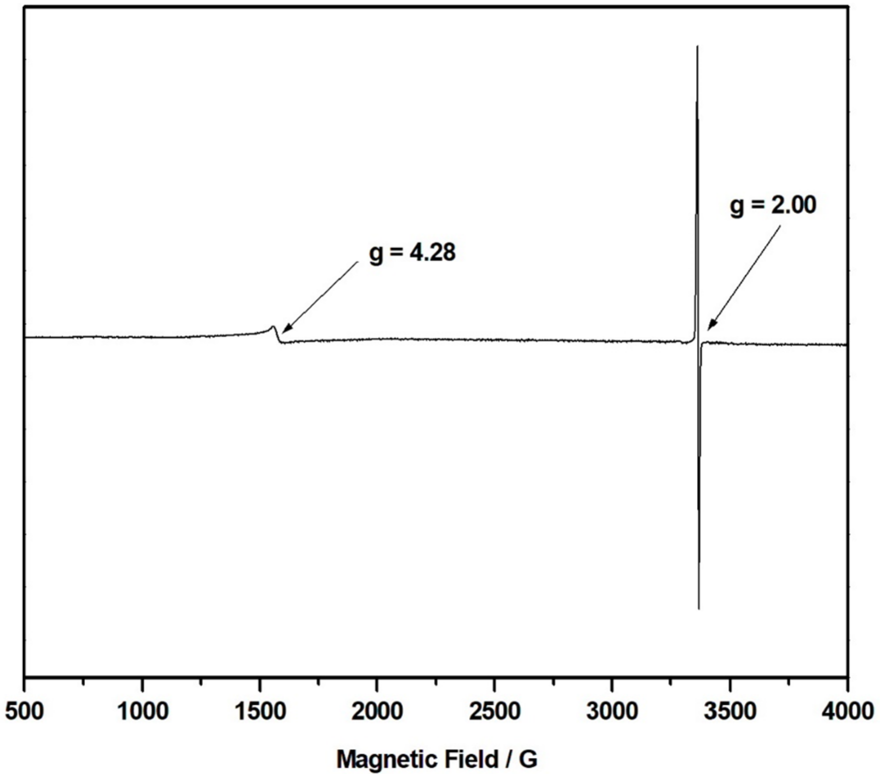

Figure S59 - EPR spectra recorded at −196°C of the reaction between **4^{Mes}** and CO in toluene after 1 day. No anisotropic splitting of the signal at g_{iso} = 2.00, indicating the signal is not due to an Fe(I) complex.

4.2.7 Mössbauer spectroscopy characterisation

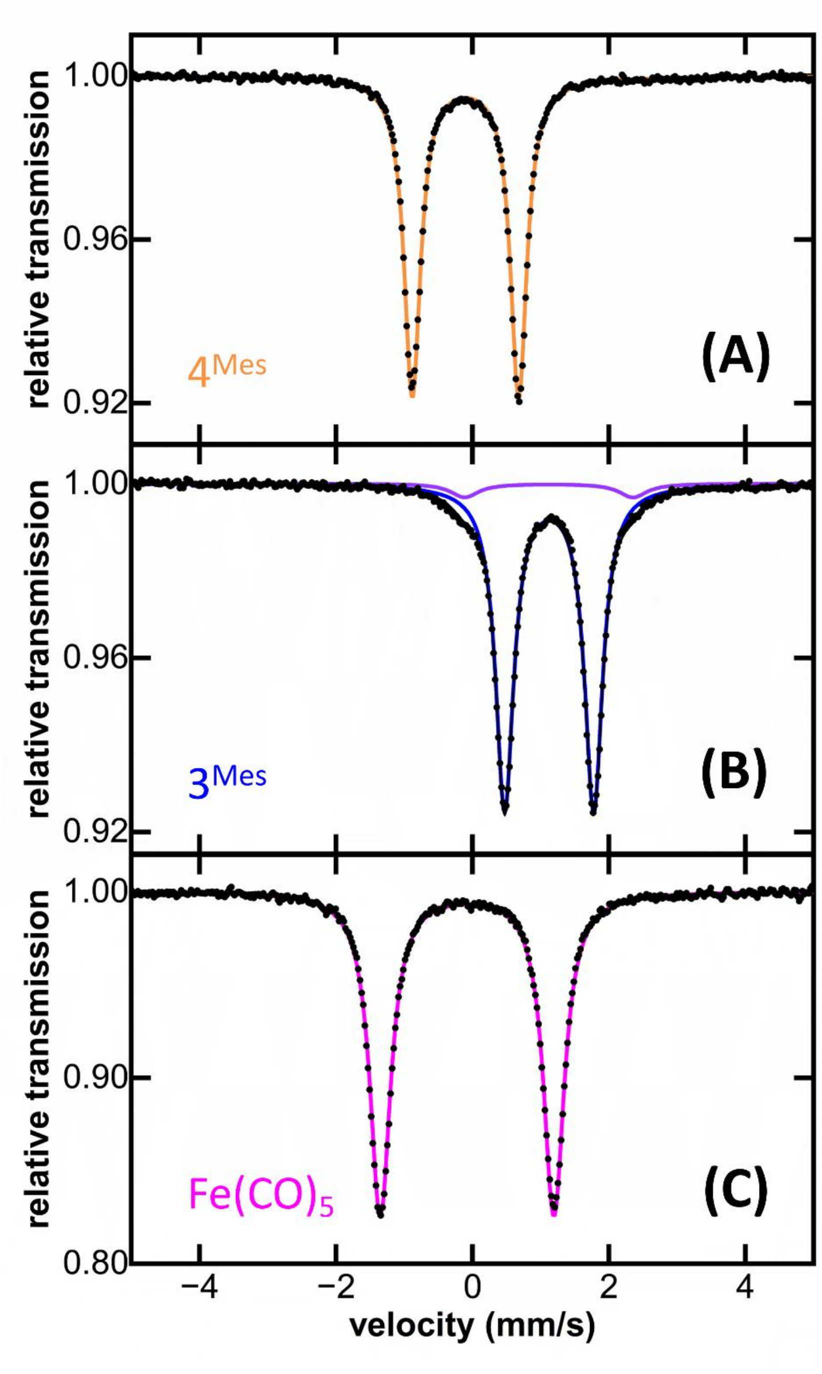

Figure S60 - 80 K Mössbauer spectrum of (A) solid $\mathbf{4^{Mes}}$ (orange), characterised by a doublet with δ = -0.10 mm/s and $|\Delta E_Q|$ = 1.56 mm/s. (B) solid $\mathbf{3^{Mes}}$ (blue) characterised by a doublet with δ = 1.12 mm/s and $|\Delta E_Q|$ = 1.30 mm/s (94%) with a minor component characterised by a doublet with δ = 1.11 mm/s and $|\Delta E_Q|$ = 2.46 mm/s (6%). (C) freeze-trapped $Fe(CO)_5$ (pink) characterised by a doublet with δ = -0.08 mm/s and $|\Delta E_Q|$ = 2.54 mm/s.[13] Raw data are shown as black dots, total fit as a black line, and individual components as coloured lines.

4.2.8 Reaction monitoring *via* Mössbauer spectroscopy

A J Young ampoule was charged with $\mathbf{4^{Mes}}$ (2 g, 2.27 mmol mmol) and dissolved in C_6H_6 (10 mL). 0.7 mL of this solution was transferred into a plastic Mössbauer cup and frozen in the glovebox freezer. Once frozen, the sample was quickly transferred from the glovebox into centrifuge tube filled with liquid nitrogen, which was subsequently submerged in a liquid nitrogen filled shipping dewar. 0.1 mL

of the solution was transferred to a J Young NMR tube and diluted with 0.5 mL of C_6H_6, for the spectra to be collected using the method described above for non-deuterated solvents. The remainder of the solution was removed from the glovebox and placed on a Schlenk line. The inert atmosphere was removed under vacuum, being careful not to remove the solvent and the flask backfilled with CO. After ~24 hours the flask was transferred back into the glovebox. 0.8 mL the of solution was then removed from the ampoule and filtered through a pipette plugged with glass fibre paper. 0.7 mL of the solution was transferred into a plastic Mössbauer cup and frozen using the method described above, and the remaining 0.1 mL was transferred to an NMR tube and diluted with 0.5 mL of C_6H_6. This process was repeated for a total of 5 days.

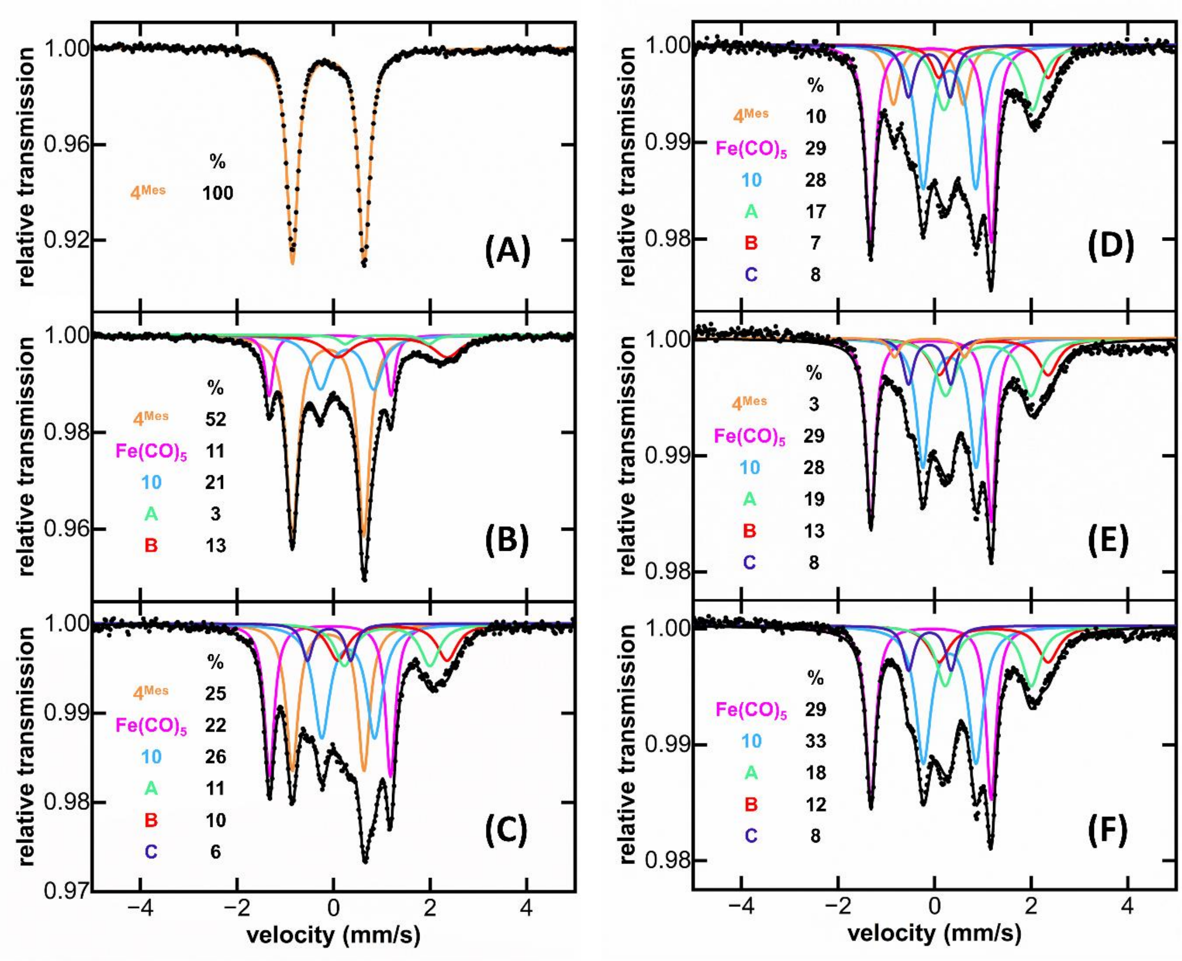

Figure S61 - Freeze-trapped 80K Mössbauer spectrum of the *in situ* formed iron species upon reaction of with CO at (A) 0 min, (B) 1 day, (C) 2 days, (D) 3 days, (E) 4 days and (F) 5 days. In the spectra were identified **4^{Mes}**, **$Fe(CO)_5$**, side products: **10**, **A**, **B** (also generated in absence of CO, see Figure S85) and additional species **C.** Assignments of the doublets are as follows: δ = 0.30 mm/s and ΔE_Q = 1.10 mm/s (**10,** light blue), δ = 1.10 mm/s and $|\Delta E_Q|$ = 1.79 mm/s **(A,** light green), δ = 1.21 mm/s and $|\Delta E_Q|$ = 2.33 mm/s (**B,** red) and δ = -0.10 mm/s and $|\Delta E_Q|$ = 0.87 mm/s (**C,** dark purple). Raw data are shown as black dots, total fit as a black line, and individual components as coloured lines**.**

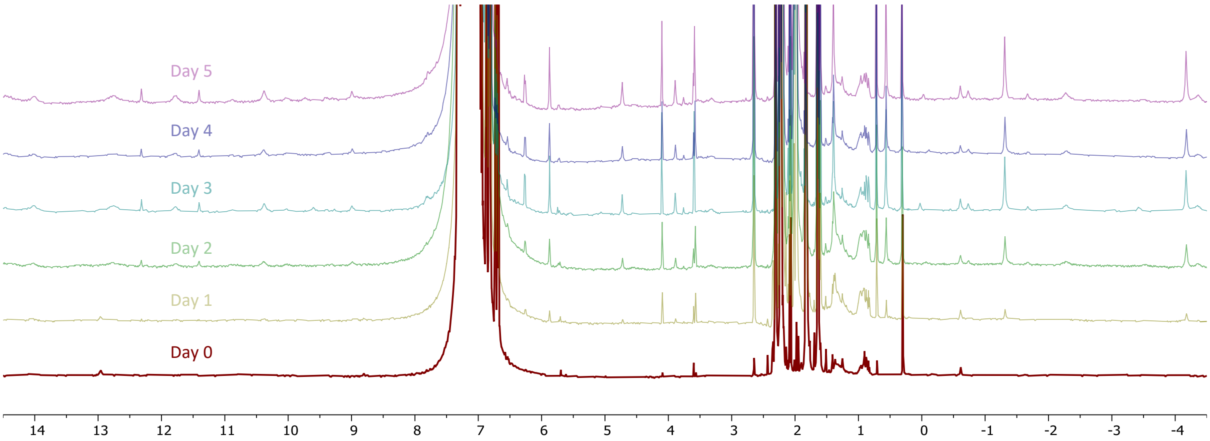

Figure S62 - ^{1}H NMR spectrum at 25°C of the reaction between $\mathbf{4}^{\mathbf{Mes}}$ and CO in C_6H_6 from 14.5 - −4.5 ppm for simultaneous Mössbauer and NMR spectroscopy monitoring over 5 days. Paramagnetic species are observed on day 5, consistent with the Mössbauer spectra obtained.

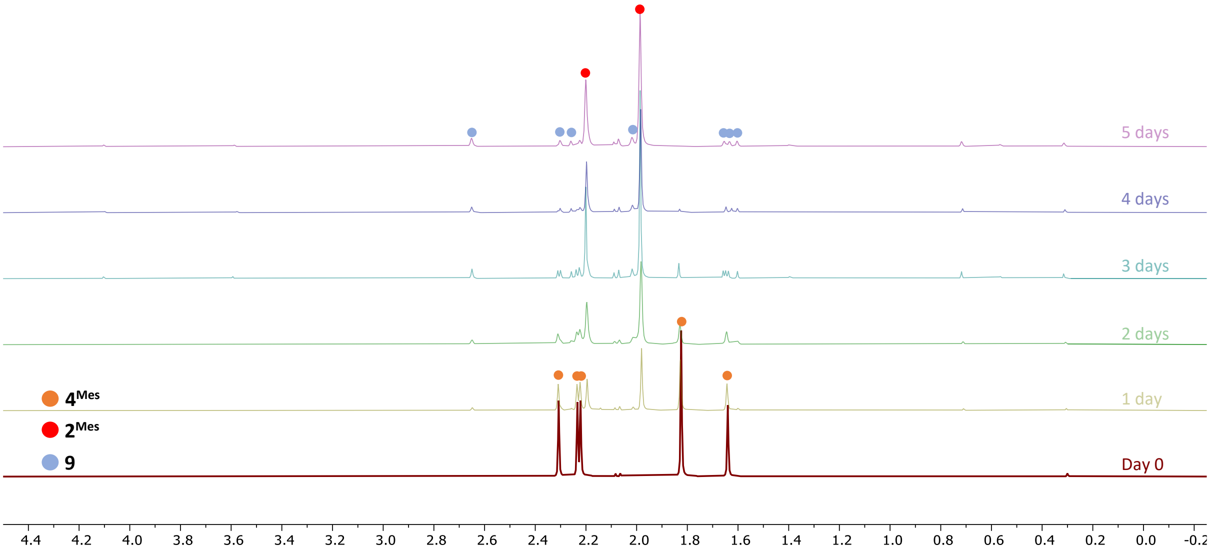

Figure S63 – Zoom in on the alkyl region of the ^{1}H NMR spectrum at 25°C for the reaction between $\mathbf{4}^{\mathbf{Mes}}$ and CO in C_6H_6 for simultaneous Mössbauer and NMR spectroscopy monitoring over 5 days.

4.2.9 Simultaneous EPR, IR and NMR sampling

To ensure that the signals observed were consistent between the three spectroscopic methods, the analysis was carried out from a single reaction with all samples removed from the reaction vessel at the same time. A J Young ampoule was charged with $\mathbf{4}^{\mathbf{Mes}}$ (600 mg, 0.72 mmol) in the glovebox and dissolved in C_6H_6 (16 mL). The flask was connected to the Schlenk line and the inert gas removed under vacuum, being careful to not remove any solvent. The flask was backfilled with CO and the vacuum/refilling process was repeated twice more. The reaction was then left to react for ~22 hours. After this time the flask was transferred to the glovebox. 1.5 mL of solution was removed from the ampoule and filtered through a pipette plugged with glass fibre paper. The filtered solution was then transferred into the EPR tube, Harrick cell and J Young NMR tube all of which are then sealed under an inert atmosphere. Each measurement was then performed within 2 hours of the sample being removed from the ampoule. The ampoule containing the remaining reaction solution was then refreshed on the Schlenk line with more CO to ensure a sufficient quantity of CO is present. Further samples were then taken approximately every 24 hours for the following four days. A further data point was collected after 8 days of reaction.

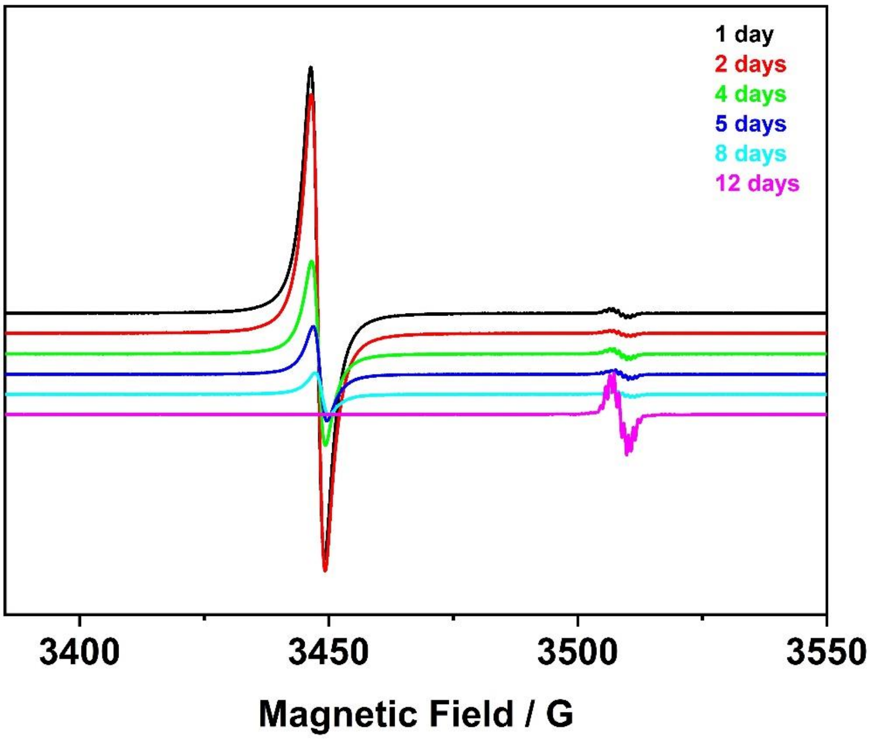

Figure S64 - EPR spectra recorded at 25°C over 12 days of the reaction between **4**^{Mes} and CO in C_6H_6. Day 1 is ~2 hours after setting up the reaction. Spectra collected from the same sample as IR/NMR spectra shown below.

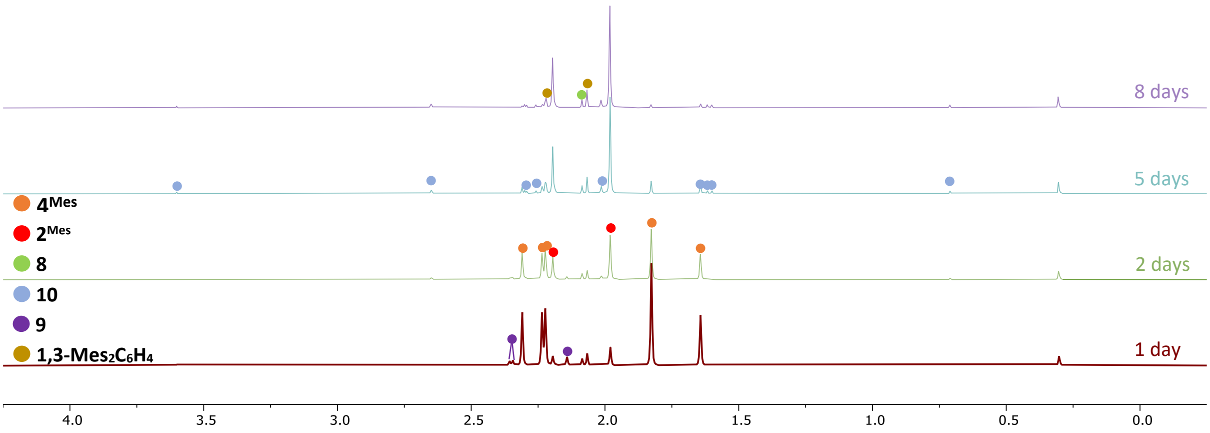

Figure S65 – Alkyl region for the ^1H NMR spectra recorded at 25°C over 8 days of the reaction between **4**^{Mes} and CO in C_6H_6. Spectra collected from the same sample as IR/EPR spectra shown above and below.

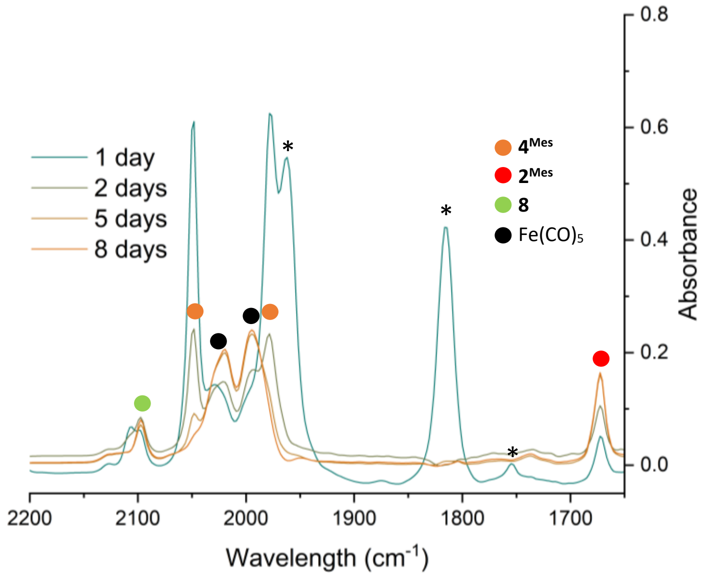

Figure S66 - IR spectra recorded over 8 days of the reaction between **4^{Mes}** and CO in C_6H_6. The spectrum after 1 day of reactivity shows three peaks relating to C_6H_6 (*) due to the cell not sealing properly, creating a longer path length, therefore the background signal from solvent cannot be removed completely. Spectra collected from the same sample as EPR/NMR spectra shown above.

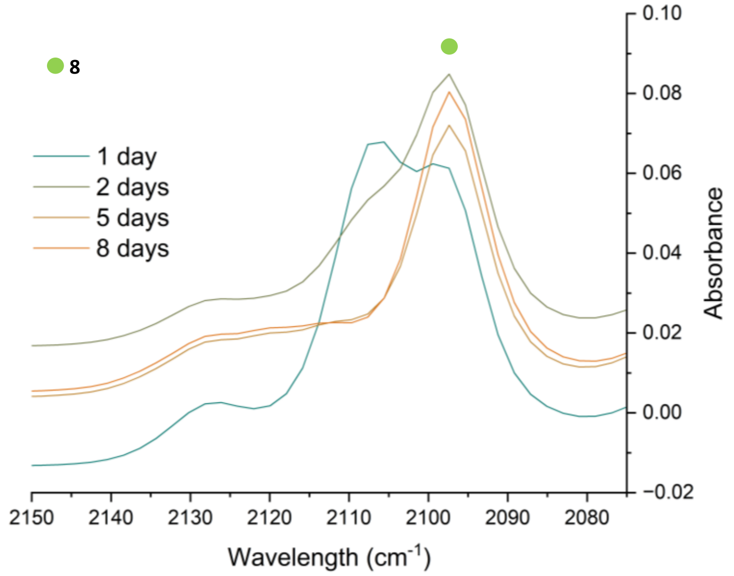

Figure S67 - Zoom in of the ketene region of the IR spectra recorded over 8 days of the reaction between **4^{Mes}** and CO in C_6H_6. Spectra collected from the same sample as EPR/NMR spectra shown above.

4.2.10 ^{1}H and ^{13}C{^{1}H} NMR, EPR and IR spectra of 4^{Mes}-^{13}C under ^{13}CO

A J Young ampoule was charged with **4^{Mes}-^{13}C** (150 mg, 0.18 mmol) in the glovebox and dissolved in C_6H_6 (6 mL). The flask was connected to the Schlenk line and the inert gas removed under vacuum, with care taken to not remove any solvent. The flask was backfilled with ^{13}CO up to a pressure of ~1 atm. The reaction was then left to react for 2 days. After this time the flask was transferred to the glovebox. ~1.5 mL of solution was then removed from the ampoule and filtered through a pipette plugged with glass fibre paper. The filtered solution was then transferred into an EPR tube, a J-Young NMR tube and a Harrick cell which are sealed under an inert atmosphere. The corresponding spectra were collected after reacting for 2 days of taking the sample. An additional set of spectra were taken after 8 days.

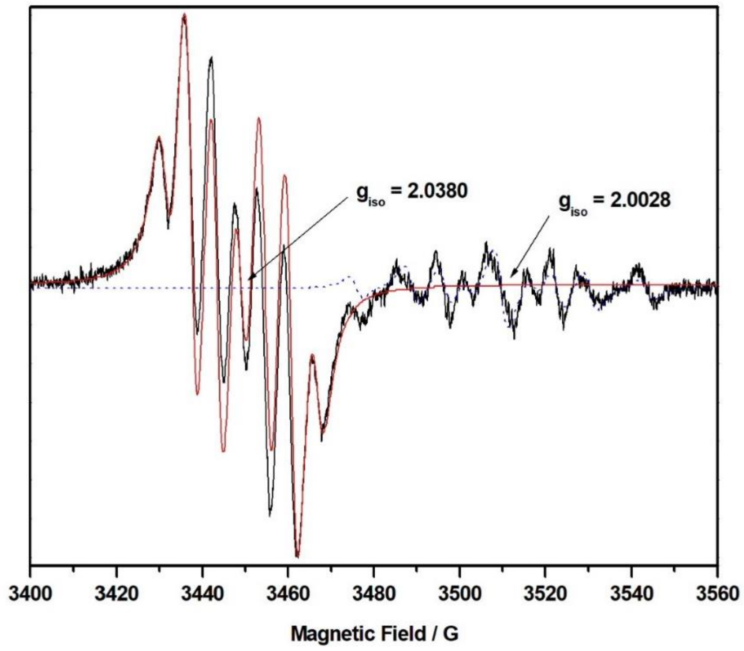

Figure S68 - Experimental X-band EPR spectrum of products formed by the reaction of **4^{Mes}-^{13}C** under an atmosphere of ^{13}CO (black line) recorded at ambient temperature as a solution in toluene. The red and blue lines represent simulations of the experimental spectrum centred at g_{iso} = 2.0380 (red, **intermediate**) and g_{iso} = 2.0028 (blue, [**2^{Mes}**]$^{\cdot -}$), respectively, using parameters given in **Table S3**.

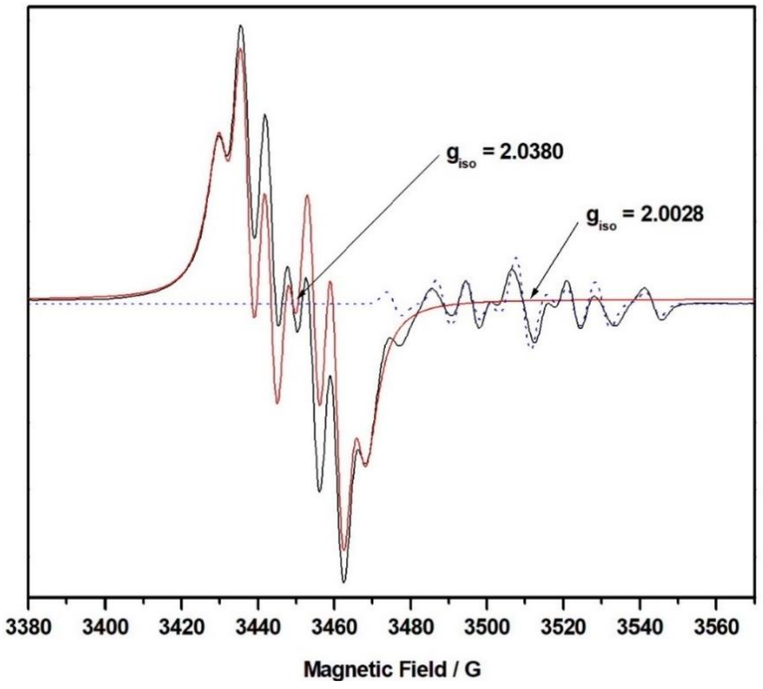

Figure S69 - Experimental X-band EPR spectrum of products formed by the reaction of **4^{Mes}-^{13}C** under an atmosphere of ^{13}CO (black line) recorded at ambient temperature as a solution in toluene. The red and blue lines represent simulations of the experimental spectrum centred at giso = 2.0380 (red, **intermediate**) and g_{iso} = 2.0028 (blue, [**2^{Mes}**]$^{\cdot -}$), respectively, using parameters given in **Table S3**.

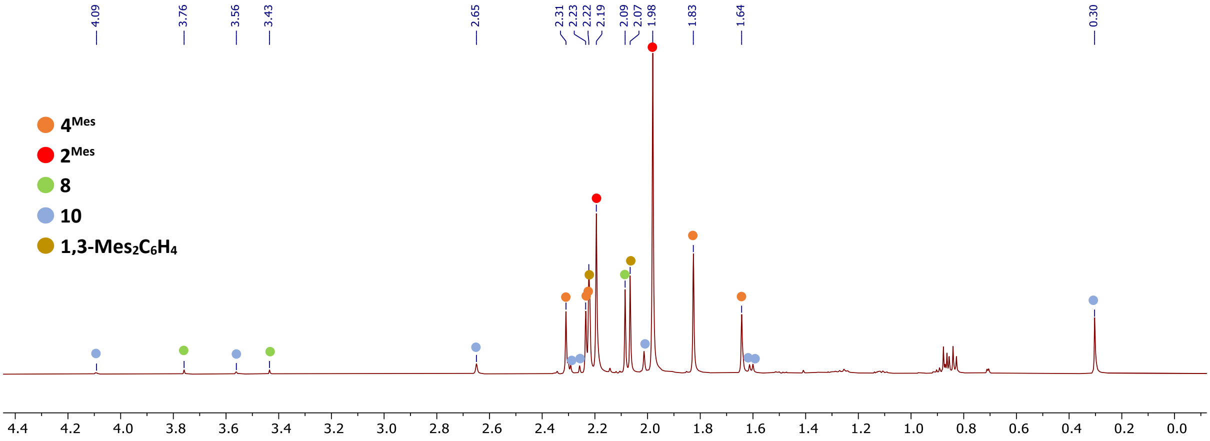

Figure S70 - Alkyl region for the ^{1}H NMR spectra recorded at 25°C for the reaction of $\mathbf{4^{Mes}}$**-**$\mathbf{^{13}C}$ under a ^{13}CO atmosphere after 2 days in C_6H_6.

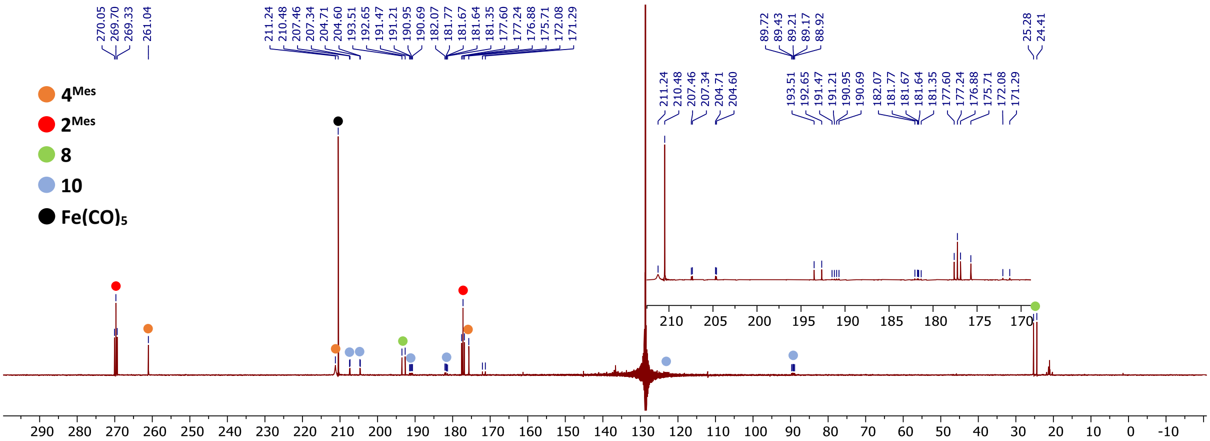

Figure S71 – $^{13}C\{^{1}H\}$ NMR spectrum at 25°C of the reaction of $\mathbf{4^{Mes}}$**-**$\mathbf{^{13}C}$ under a ^{13}CO atmosphere after 2 days in C_6H_6. Peaks marked are ^{13}C enriched.

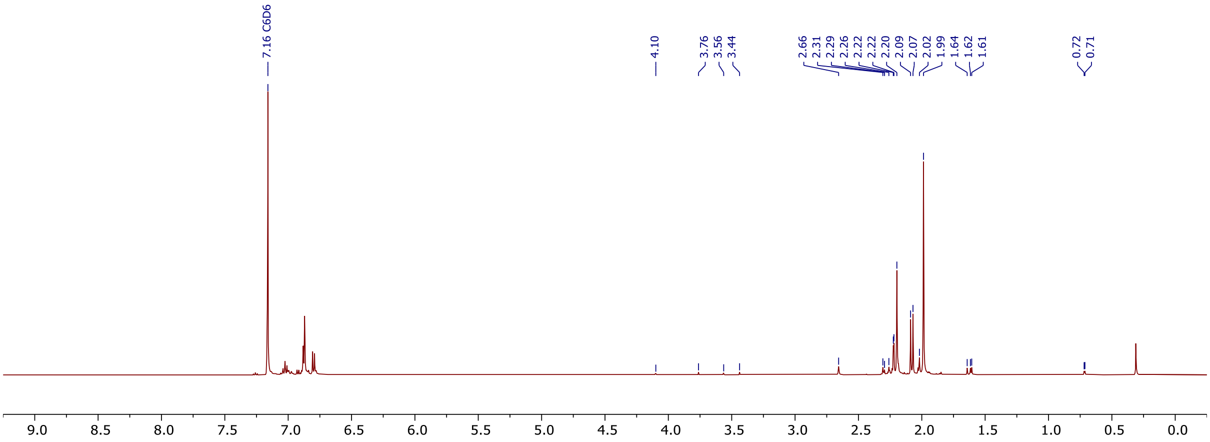

Figure S72 - ^{1}H NMR spectrum at 25°C of the reaction of $\mathbf{4^{Mes}}$**-**$\mathbf{^{13}C}$ under a ^{13}CO atmosphere after 8 days. Volatiles were removed and the spectrum collected in C_6D_6.

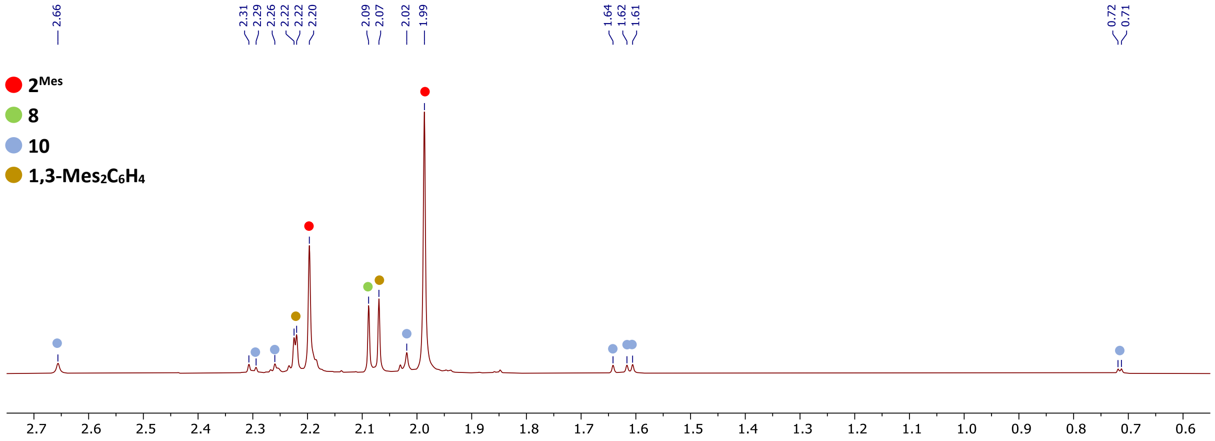

Figure S73 - Zoom in on the alkyl region for the ^{1}H NMR spectrum at 25°C of the reaction of **4^{Mes}-^{13}C** under a ^{13}CO atmosphere after 8 days. Volatiles were removed and the spectrum collected in C_6D_6.

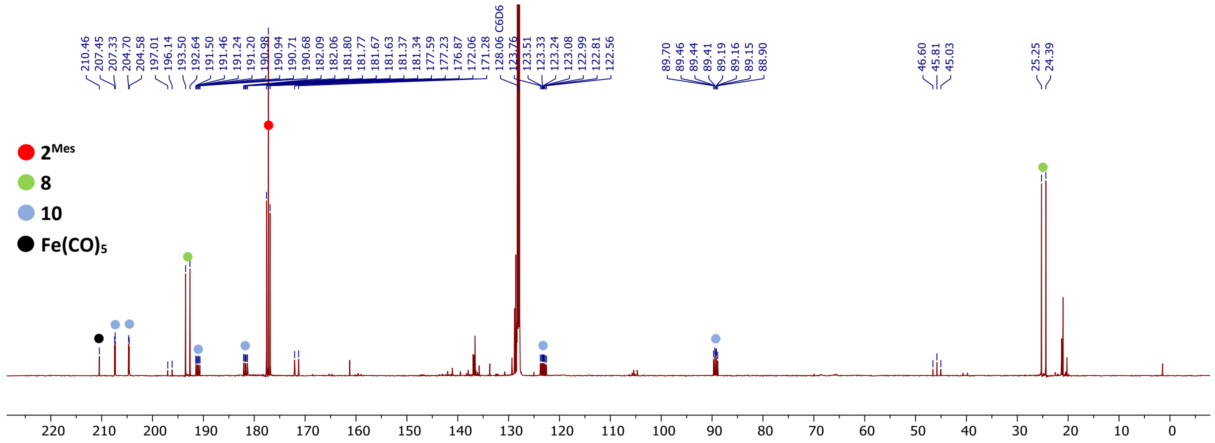

Figure S74 - ^{13}C{^{1}H} NMR spectrum at 25°C of the reaction of **4^{Mes}-^{13}C** under a^{13}CO atmosphere after 2 days in C_6H_6. Volatiles were removed and the spectrum collected in C_6D_6. Peaks marked are ^{13}C enriched.

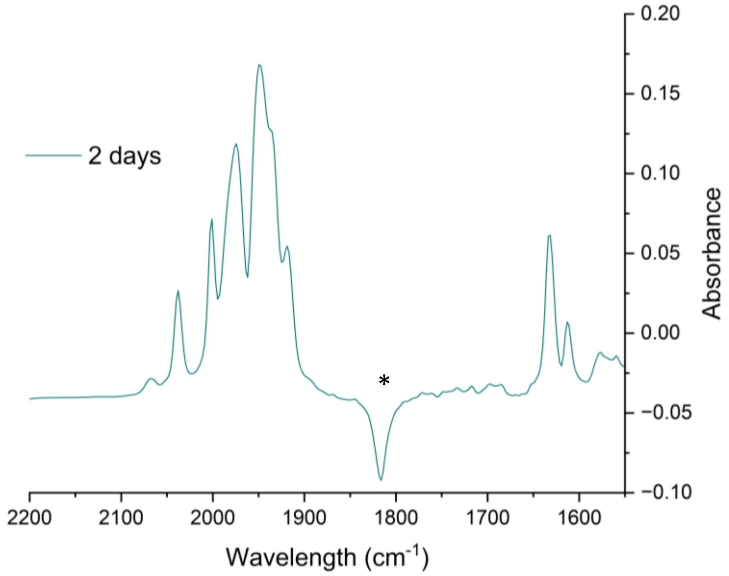

Figure S75 - IR spectra recorded after 2 days of the reaction between **4^{Mes}-^{13}C** and ^{13}CO in C_6H_6. The spectrum shows a strong negative peak relating to C_6H_6 (*) sealing properly for the baseline measurement, creating a longer path length. Spectra collected from the same sample as EPR/NMR spectra shown above.

4.2.11 ^{1}H and $^{13}C\{^{1}H\}$ NMR, EPR and IR spectra of 4^{Mes}-^{13}C under natural abundance CO

A J Young ampoule was charged with **4^{Mes}-^{13}C** (150 mg, 0.18 mmol) in the glovebox and dissolved in C_6H_6 (6 mL). The flask was connected to the Schlenk line and the inert gas removed under vacuum, being careful to not remove any solvent. The flask was backfilled with CO and the vacuum/refilling process was repeated twice more. The reaction was then left to react for ~22 hours. After this time the flask was transferred to the glovebox. ~0.6 mL of solution was then removed from the ampoule and filtered through a pipette plugged with glass fibre paper. The filtered solution is then transferred into a J-Young NMR tube which was then sealed under an inert atmosphere. The ampoule containing the remaining reaction solution was stirred for a further 8 days, with samples taken on days 2-4.

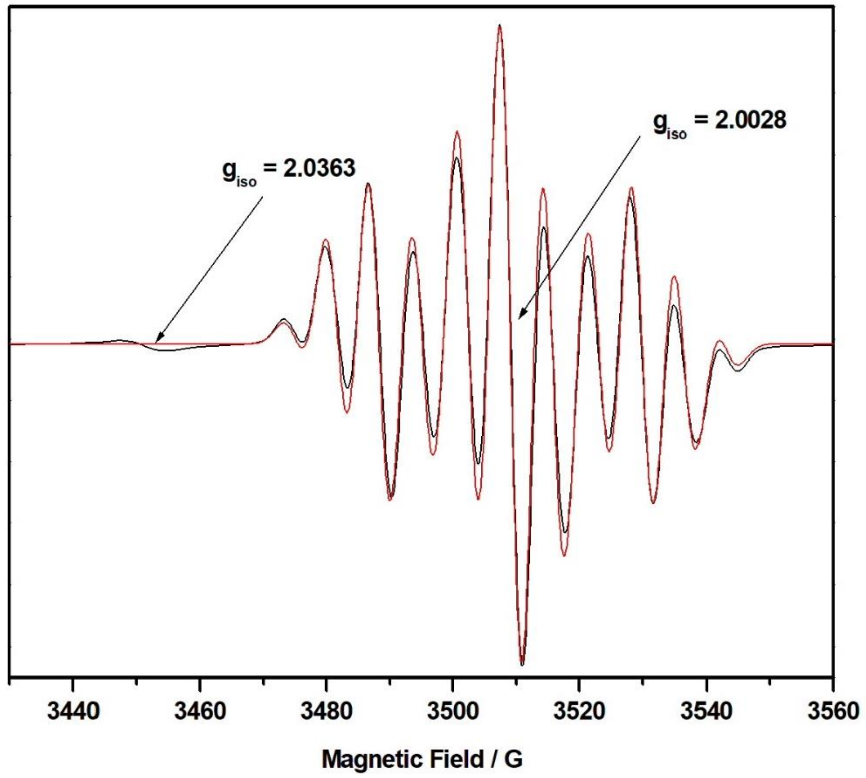

Figure S76 - Experimental X-band EPR spectrum of [**2^{Mes}**]$^{\cdot -}$ formed by the reaction of **4^{Mes}-^{13}C** under an atmosphere of natural abundance CO (black line) recorded at ambient temperature as a solution in toluene. The red line represents a simulation of the experimental spectrum using parameters given in **Table S3**.

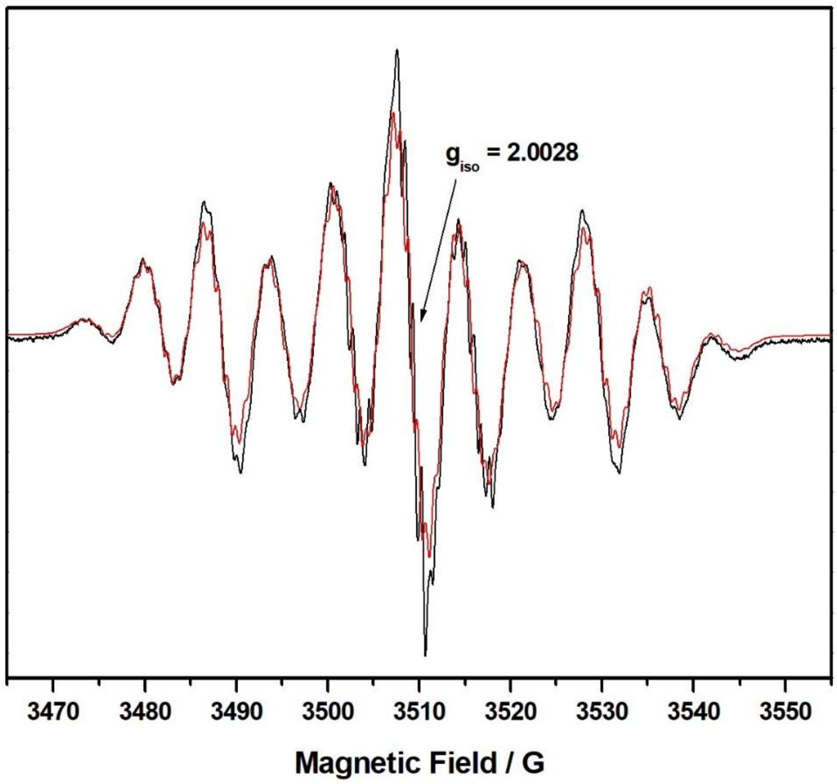

Figure S77 - Experimental X-band EPR spectrum of [**2**$^{\mathbf{Mes}}$]$^{\cdot-}$ formed by the reaction of **4**$^{\mathbf{Mes}}$**-**$^{\mathbf{13}}$**C** under an atmosphere of natural abundance CO (black line) recorded at ambient temperature as a solution in toluene. The red line represents a simulation of the experimental spectrum using parameters given in **Table S3**.

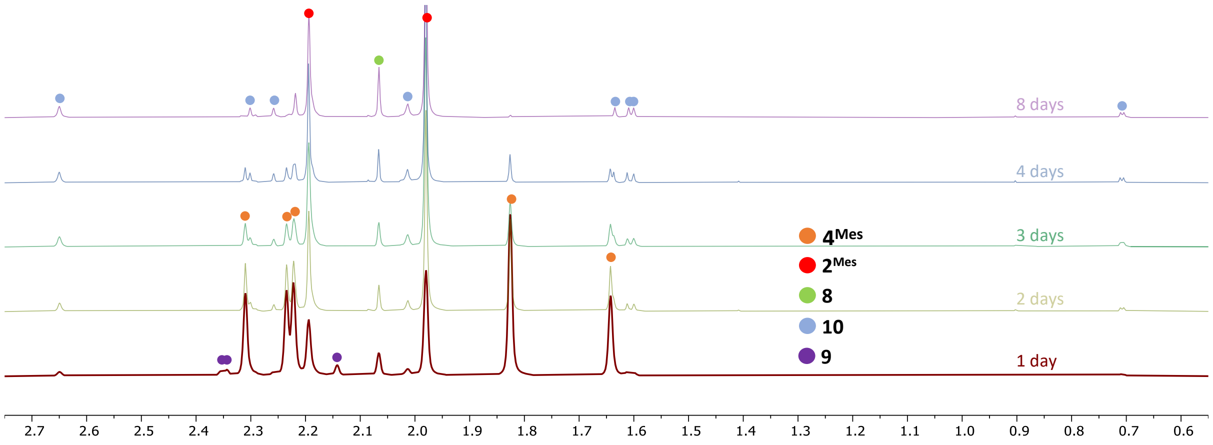

Figure S78 – Alkyl region for the ^{1}H NMR spectra recorded at 25°C over 8 days of the reaction between **4**$^{\mathbf{Mes}}$**-**$^{\mathbf{13}}$**C** and natural abundance CO in C_6H_6. Spectra collected from the same sample as EPR/IR spectra shown above and below.

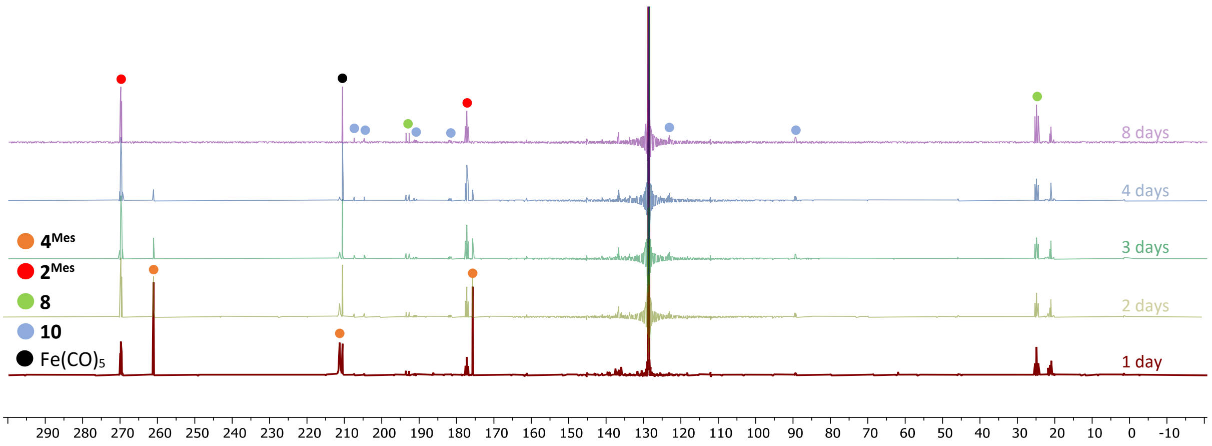

Figure S79 – $^{13}C\{^{1}H\}$ NMR spectra recorded at 25°C over 8 days of the reaction between **4^{Mes}-^{13}C** and natural abundance CO in C_6H_6. Enriched signals highlighted. Spectra collected from the same sample as EPR/IR spectra shown above and below.

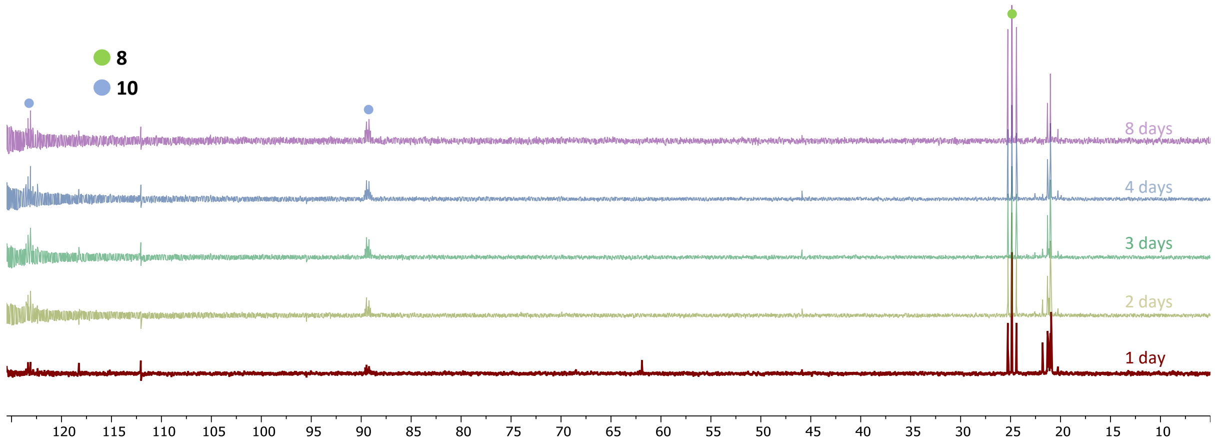

Figure S80 – Zoom in between 5 and 125.5 ppm of the $^{13}C\{^{1}H\}$ NMR spectra recorded at 25°C over 8 days of the reaction between **4^{Mes}-^{13}C** and natural abundance CO in C_6H_6. Enriched signals highlighted. Spectra collected from the same sample as EPR/IR spectra shown above and below.

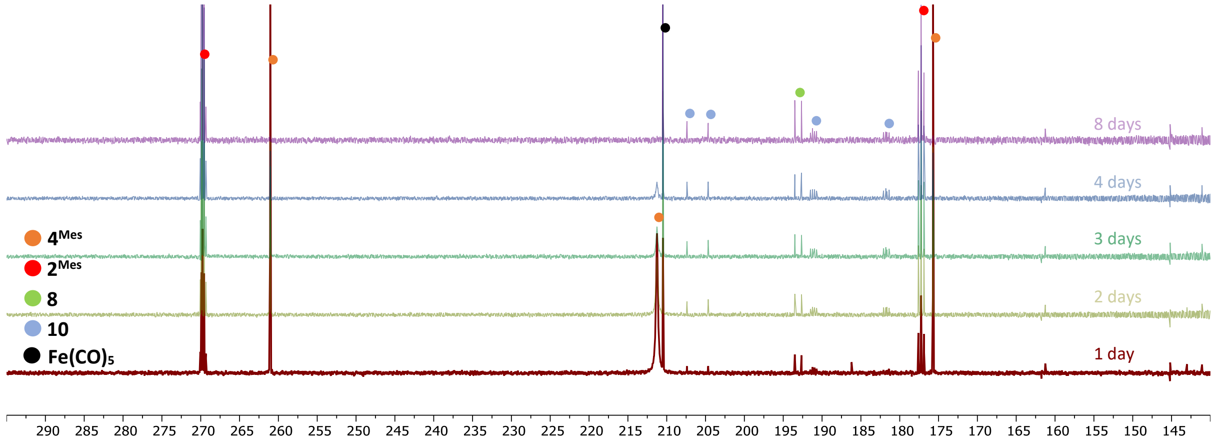

Figure S81 – Zoom in between 140 and 290 ppm of the $^{13}C\{^{1}H\}$ NMR spectra recorded at 25°C over 8 days of the reaction between **4^{Mes}-^{13}C** and natural abundance CO in C_6H_6. Enriched signals highlighted. Spectra collected from the same sample as EPR/IR spectra shown above and below.

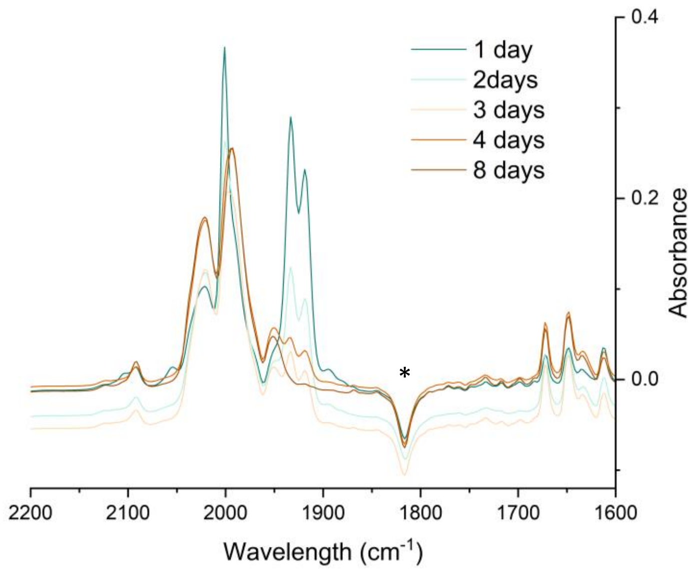

Figure S82 - IR spectra recorded after 2 days of the reaction between **4^{Mes}-^{13}C** and natural abundance CO in C_6H_6. The spectrum shows a strong negative peak and a downward shift in the signals relating to C_6H_6 (*) due to the cell not sealing correctly for the baseline measurement, creating a longer path length. Spectra collected from the same sample as EPR/NMR spectra shown above.

4.2.12 ^{1}H, ^{13}C{^{1}H} NMR and Mössbauer spectrum of 4^{Mes} after one month in the absence of CO

10 NMR tubes were filled with **4^{Mes}** (25 mg [0.03 mmol] × 10) and C_6H_6 (0.6 mL × 10) and thoroughly mixed. After 1 month, the solutions were combined and filtered through a pipette fitted with a glass fibre filter, washing the precipitate with C_6H_6 until the washings were colourless. The solvent was removed *in vacuo* yielding a red solid. To the solid was added *iso*-hexane (5 mL) and placed in the freezer to precipitate out **2^{Mes}**. The solution was filtered again and the solvent removed *in vacuo*. The remaining oily solid was extracted into C_6D_6 and the NMR collected. The sample contains 4 species that can be identified: 2,6-Mes-C_6H_4, **2^{Mes}**, **8** and **10**.

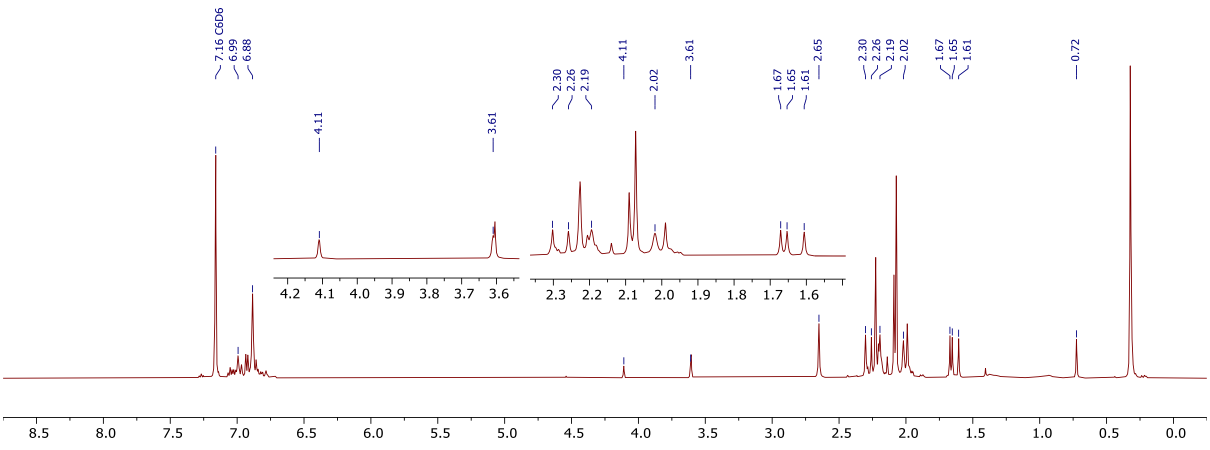

Figure S83 – ^{1}H NMR spectrum at 25°C of the reaction of **4^{Mes}** in the absence of a CO atmosphere. The spectrum contains at least 4 species.

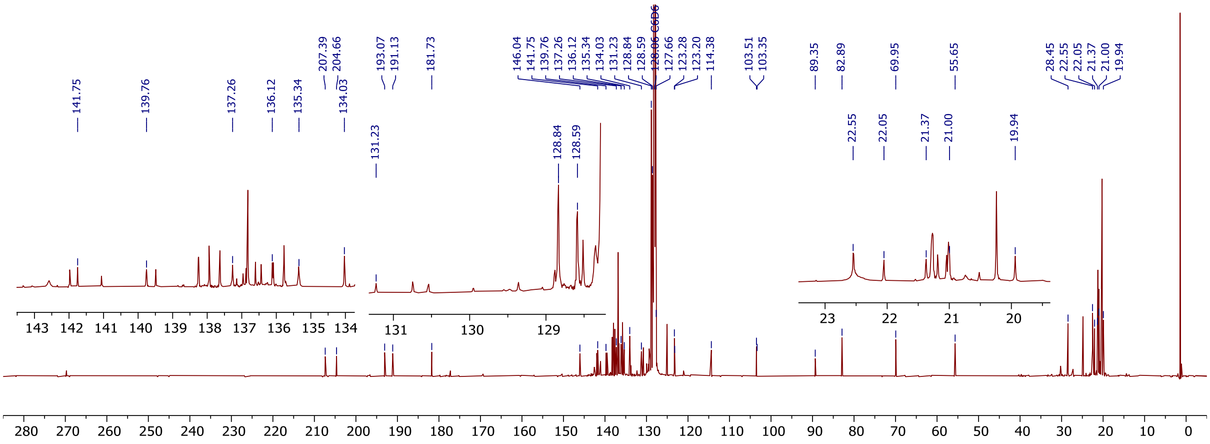

Figure S84 – $^{13}C\{^{1}H\}$ NMR spectrum at 25°C of the reaction of $\mathbf{4^{Mes}}$ in the absence of a CO atmosphere. The spectrum contains at least 4 species.

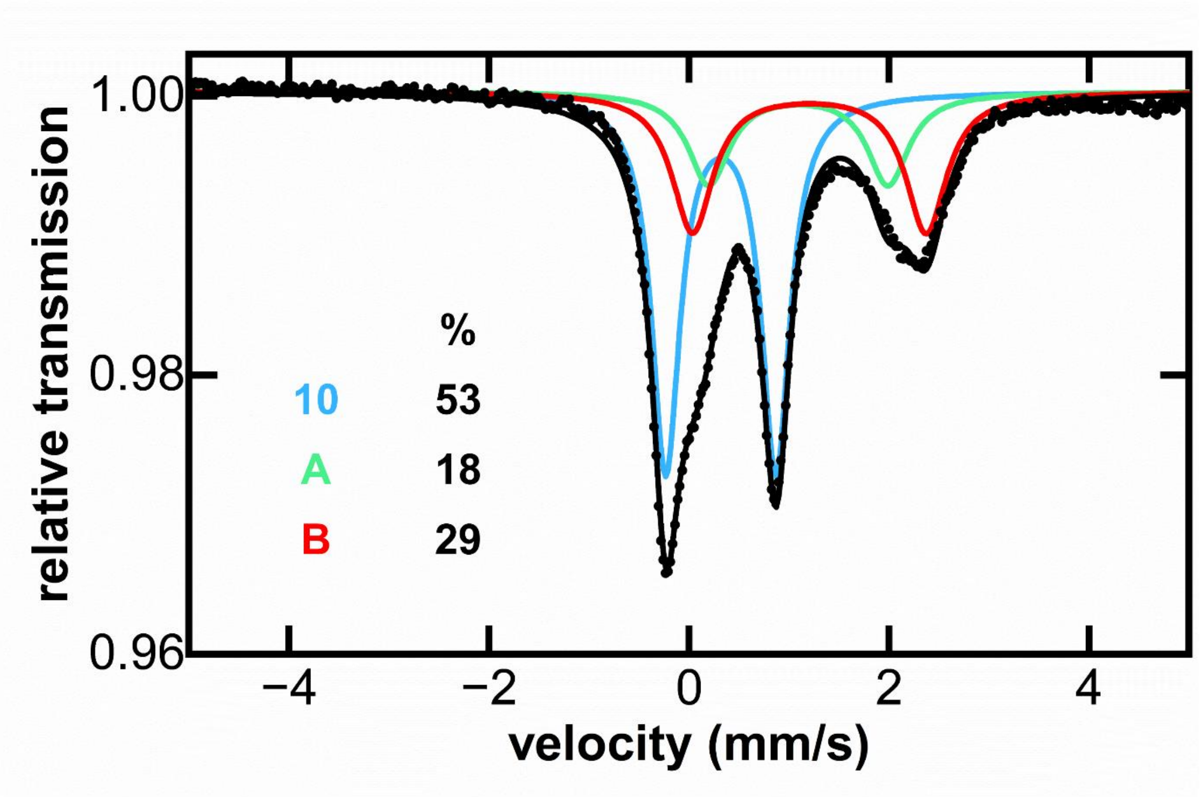

Figure S85 - Freeze-trapped 80K Mössbauer spectrum of the in situ formed iron species upon reaction of $\mathbf{4^{Mes}}$ in absence of CO. Assignments of the doublets are as follows: δ = 0.30 mm/s and ΔE_Q = 1.10 mm/s (**10,** light blue), δ = 1.10 mm/s and $|\Delta E_Q|$ = 1.79 mm/s **(A,** light green) and δ = 1.21 mm/s and $|\Delta E_Q|$ = 2.33 mm/s (**B,** red). Raw data are shown as black dots, total fit as a black line, and individual components as colored lines.

4.3 EPR fitting parameters

Table S3 - Fitting parameters used in modelling the EPR spectra obtained. Percentages for the mixed $^{12/13}C$ species are in line with ratios observed in the $^{13}C\{^{1}H\}$ spectra for these species.

	g_{iso}	a_{iso} / x 10^{-4} cm^{-1}	Lineshape	Linewidth / G
**Figure S58**<br>**[2^{Mes}]$^{\cdot -}$** (all ^{12}C)	2.0028	2 ^{1}H 1.59<br>4 ^{1}H 0.90	Gaussian	0.84
**Figure S68**<br>**Intermediate** (tentative)	2.0380 (strong)	1 ^{13}C (100%) 16.40<br>3 ^{13}C (100%) 5.72	Lorentzian	3.80
**[2^{Mes}]$^{\cdot -}$** (all ^{13}C)	2.0028 (weak)	2 ^{13}C (100%) 19.44<br>2 ^{13}C (100%) 12.20	Lorentzian	3.40
**Figure S69**<br>**Intermediate** (tentative)	2.0380 (strong)	1 ^{13}C (100%) 16.40<br>3 ^{13}C (100%) 5.72	Lorentzian	4.70
**[2^{Mes}]$^{\cdot -}$** (all ^{13}C)	2.0028 (weak)	2 ^{13}C (100%) 19.44<br>2 ^{13}C (100%) 12.20	Gaussian	3.90
**Figure S76**<br>**[2^{Mes}]$^{\cdot -}$** ($^{12/13}C$)	2.0028	2 ^{13}C (100%) 19.44<br>2 ^{13}C (43%) 12.20	Gaussian	4.00
**Figure S77**<br>**[2^{Mes}]$^{\cdot -}$** ($^{12/13}C$)	2.0028	2 ^{13}C (100%) 19.44<br>2 ^{13}C (43%) 12.20<br>2 ^{1}H 1.59<br>4 ^{1}H 0.90	Lorentzian	0.97

5. Computational Details

Density functional theory (DFT) calculations were performed with the ωB97X-D3 functional[13,14] and def2-SV(P) basis set and pseudopotential[12] as implemented in the Q-Chem 5.4.1 software package.[13] Geometry optimisations were performed for all potential reactants, intermediates and products up to **2**^{Mes}, with both low-spin and high-spin iron centres considered. Transition states were determined for all points on the reaction pathway at which the iron centre remained low-spin (up to and including **4**^{Mes}). Frequency calculations were used to determine the nature of the stationary point obtained.

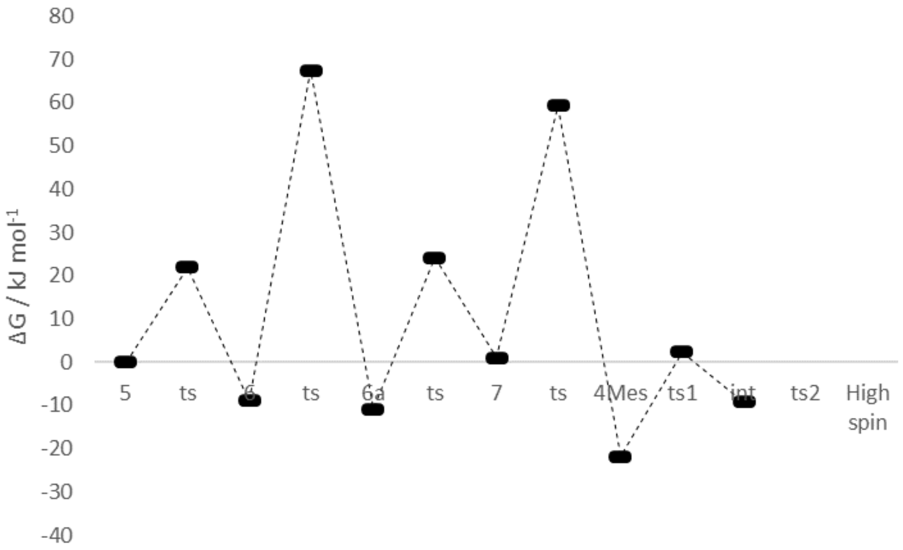

Figure S86 - Free energy reaction profile from the gas-phase ωB97X-D3/SRSC calculations. The barrier height for the transition state between **4** and **4a** is somewhat higher than expected. We expect that in experiments, explicit contact with solvent and other factors not included in the calculations would lower this barrier, as this step occurs on a fast timescale. It is likely that the relative heights of **5** and **2** are also likely to be affected in a similar manner. Additional calculations were performed for these molecules with implicit solvent, which only gave a modest change (less than 4 kJ mol^{-1} in all cases).

6. References

1. D. L. K. (née Coombs) and A. R. Cowley, *Chem. Commun.*, 2007, 1053–1055.
2. H. R. Sharpe, A. M. Geer, L. J. Taylor, B. M. Gridley, T. J. Blundell, A. J. Blake, E. S. Davies, W. Lewis, J. McMaster, D. Robinson and D. L. Kays, *Nat Commun*, 2018, **9**, 3757.
3. J. D. Winter, G. Deshayes, F. Boon, O. Coulembier, P. Dubois and P. Gerbaux, *J. Mass Spectrom.*, 2011, **46**, 237–246.
4. D. R. Allan, H. Nowell, S. A. Barnett, M. R. Warren, A. Wilcox, J. Christensen, L. K. Saunders, A. Peach, M. T. Hooper, L. Zaja, S. Patel, L. Cahill, R. Marshall, S. Trimnell, A. J. Foster, T. Bates, S. Lay, M. A. Williams, P. V. Hathaway, G. Winter, M. Gerstel and R. W. Wooley, *Crystals*, 2017, **7**, 336.
5. Rigaku Oxford Diffraction, (2018), CrysAlisPro Software system, version 1.171.40.45a, Rigaku Corporation, Oxford, UK.
6. G. Winter, D. G. Waterman, J. M. Parkhurst, A. S. Brewster, R. J. Gildea, M. Gerstel, L. Fuentes-Montero, M. Vollmar, T. Michels-Clark, I. D. Young, N. K. Sauter and G. Evans, *Acta Cryst D*, 2018, **74**, 85–97.

7.AIMLESS (CCP4: Supported Program): Journ.al, 2018, CCP4 7.0.062: AIMLESS, version 060.067.062: 027/005/018.
8. O. V. Dolomanov, L. J. Bourhis, R. J. Gildea, J. a. K. Howard and H. Puschmann, *J. Appl. Cryst.*, 2009, **42**, 339–341.
9. G. M. Sheldrick, *Acta Cryst A*, 2015, **71**, 3–8.
10. G. M. Sheldrick, *Acta Cryst C*, 2015, **71**, 3–8.
11. The facility "CheckCIF," can be found at http://checkcif.iucr.org.
12. A. L. Spek, *Acta Cryst C*, 2015, **71**, 9–18.
13. R. H. Herber, W. R. Kingston and G. K. Wertheim, *Inorg. Chem.*, 1963, **2**, 153–158.
14. J.-D. Chai and M. Head-Gordon, *Phys. Chem. Chem. Phys.*, 2008, **10**, 6615–6620.
15. Y.-S. Lin, G.-D. Li, S.-P. Mao and J.-D. Chai, *J. Chem. Theory Comput.*, 2013, **9**, 263–272.
16. E. Epifanovsky, A. T. B. Gilbert, X. Feng, J. Lee, Y. Mao, N. Mardirossian, P. Pokhilko, A. F. White, M. P. Coons, A. L. Dempwolff, Z. Gan, D. Hait, P. R. Horn, L. D. Jacobson, I. Kaliman, J. Kussmann, A. W. Lange, K. U. Lao, D. S. Levine, J. Liu, S. C. McKenzie, A. F. Morrison, K. D. Nanda, F. Plasser, D. R. Rehn, M. L. Vidal, Z.-Q. You, Y. Zhu, B. Alam, B. J. Albrecht, A. Aldossary, E. Alguire, J. H. Andersen, V. Athavale, D. Barton, K. Begam, A. Behn, N. Bellonzi, Y. A. Bernard, E. J. Berquist, H. G. A. Burton, A. Carreras, K. Carter-Fenk, R. Chakraborty, A. D. Chien, K. D. Closser, V. Cofer-Shabica, S. Dasgupta, M. de Wergifosse, J. Deng, M. Diedenhofen, H. Do, S. Ehlert, P.-T. Fang, S. Fatehi, Q. Feng, T. Friedhoff, J. Gayvert, Q. Ge, G. Gidofalvi, M. Goldey, J. Gomes, C. E. González-Espinoza, S. Gulania, A. O. Gunina, M. W. D. Hanson-Heine, P. H. P. Harbach, A. Hauser, M. F. Herbst, M. Hernández Vera, M. Hodecker, Z. C. Holden, S. Houck, X. Huang, K. Hui, B. C. Huynh, M. Ivanov, Á. Jász, H. Ji, H. Jiang, B. Kaduk, S. Kähler, K. Khistyaev, J. Kim, G. Kis, P. Klunzinger, Z. Koczor-Benda, J. H. Koh, D. Kosenkov, L. Koulias, T. Kowalczyk, C. M. Krauter, K. Kue, A. Kunitsa, T. Kus, I. Ladjánszki, A. Landau, K. V. Lawler, D. Lefrancois, S. Lehtola, R. R. Li, Y.-P. Li, J. Liang, M. Liebenthal, H.-H. Lin, Y.-S. Lin, F. Liu, K.-Y. Liu, M. Loipersberger, A. Luenser, A. Manjanath, P. Manohar, E. Mansoor, S. F. Manzer, S.-P. Mao, A. V. Marenich, T. Markovich, S. Mason, S. A. Maurer, P. F. McLaughlin, M. F. S. J. Menger, J.-M. Mewes, S. A. Mewes, P. Morgante, J. W. Mullinax, K. J. Oosterbaan, G. Paran, A. C. Paul, S. K. Paul, F. Pavošević, Z. Pei, S. Prager, E. I. Proynov, Á. Rák, E. Ramos-Cordoba, B. Rana, A. E. Rask, A. Rettig, R. M. Richard, F. Rob, E. Rossomme, T. Scheele, M. Scheurer, M. Schneider, N. Sergueev, S. M. Sharada, W. Skomorowski, D. W. Small, C. J. Stein, Y.-C. Su, E. J. Sundstrom, Z. Tao, J. Thirman, G. J. Tornai, T. Tsuchimochi, N. M. Tubman, S. P. Veccham, O. Vydrov, J. Wenzel, J. Witte, A. Yamada, K. Yao, S. Yeganeh, S. R. Yost, A. Zech, I. Y. Zhang, X. Zhang, Y. Zhang, D. Zuev, A. Aspuru-Guzik, A. T. Bell, N. A. Besley, K. B. Bravaya, B. R. Brooks, D. Casanova, J.-D. Chai, S. Coriani, C. J. Cramer, G. Cserey, A. E. DePrince III, R. A. DiStasio Jr., A. Dreuw, B. D. Dunietz, T. R. Furlani, W. A. Goddard III, S. Hammes-Schiffer, T. Head-Gordon, W. J. Hehre, C.-P. Hsu, T.-C. Jagau, Y. Jung, A. Klamt, J. Kong, D. S. Lambrecht, W. Liang, N. J. Mayhall, C. W. McCurdy, J. B. Neaton, C. Ochsenfeld, J. A. Parkhill, R. Peverati, V. A. Rassolov, Y. Shao, L. V. Slipchenko, T. Stauch, R. P. Steele, J. E. Subotnik, A. J. W. Thom, A. Tkatchenko, D. G. Truhlar, T. Van Voorhis, T. A. Wesolowski, K. B. Whaley, H. L. Woodcock III, P. M. Zimmerman, S. Faraji, P. M. W. Gill, M. Head-Gordon, J. M. Herbert and A. I. Krylov, *J. Chem. Phys.*, 2021, **155**, 084801.
